# A global dataset of land-atmosphere coupling metrics based on observations for evaluating Earth system models

**DOI:** 10.1038/s41597-026-07519-2

**Published:** 2026-06-12

**Authors:** Nazanin Tavakoli, Paul A. Dirmeyer

**Affiliations:** 1 Department of Atmospheric, Oceanic, and Earth Sciences, George Mason University, Fairfax, Virginia USA; 2 Center for Ocean-Land-Atmosphere Studies, George Mason University, Fairfax, Virginia USA

## Abstract

A challenge in validating land-atmosphere (L-A) coupling in Earth system models is the limited availability of global observation-based datasets for estimating coupling metrics. This study generates global L-A coupling indices of Pearson correlation coefficient, terrestrial coupling index between soil moisture (SM) and surface heat fluxes, as well as SM memory, at resolutions of 0.1° and 0.25° (with temporal coverage depending on input datasets), employing observationally-based data. The metrics are designed to account for random errors in satellite SM measurements and include quantification of those errors. Multiple SM and flux data products are combined to produce coupling metrics – their spread and error characteristics are used to quantify uncertainty. The global patterns of SM regimes and SM breakpoints of wilting point and critical SM are also quantified through segmented regression analysis. The dataset’s performance is validated through inter-product comparisons, documenting high consistency in spatial patterns across different combinations. These datasets are valuable for initialization and validation of weather forecasts, climate modeling, model calibration, and applications in ecology, hydrology, and soil sciences.

## Background & Summary

Land-Atmosphere (L-A) interactions are a set of physical processes and feedback mechanisms within the Earth system. The land-surface states serve as initial elements in the feedback mechanisms, and soil moisture (SM) is among its most influential components. Specifically, SM plays a critical role in shaping climate and weather conditions^1,2^. Most of the inferred impacts of SM on the climate system stem from its role in the evolution of surface heat fluxes (HF), thereby influencing the water and energy cycles. In particular, SM levels can act to partition the available energy at the land surface between latent (LE) and sensible (H) heat fluxes, which in turn affect near-surface atmospheric conditions, boundary-layer properties, and ultimately cloud formation and precipitation^3–5^. As a result, the impacts of SM on surface fluxes have a fundamental influence not only on atmospheric predictability at daily-to-seasonal time scales^6–10^, but also on extremes such as droughts and heatwaves^11–14^. This underscores the necessity for a framework that simplifies these coupled interactions into measurable quantities.

To address this need, coupling metrics have been developed that combine statistical techniques with physical process understanding^15–17^. One purpose of these metrics is to further improve weather predictability and climate forecasts by providing a means to validate Earth system models^18–20^. One type, bivariate metrics, quantifies the relationship between two components, such as SM and HF. The most widely used bivariate metrics are correlation-based, including the Pearson correlation coefficient and the related terrestrial coupling index^15,21^, which quantify the strength of SM-HF interactions.

Meanwhile, soil moisture memory (SMM) is a univariate metric relevant to the persistence of feedbacks^22,23^. SMM can be conceptualized as the time required for a SM perturbation to be dissipated from a soil column. Such perturbations can result from wet anomalies, like heavy precipitation, or dry anomalies, such as drought. Since this metric reflects a boundary condition to the atmosphere, connected to the exchange of fluxes between land and atmosphere^8,24–26^, it is one of the key sources of forecast skill^1,27–30^.

Beyond these metrics, the definition of SM regimes offers complementary insights into the functional link between SM and energy partitioning. Inspired by the seminal studies of Manabe^31^ and Sellers *et al*.^32^, papers by Koster *et al*.^33^ and Seneviratne *et al*.^5^ introduced a conceptual framework to describe the relationship between SM and evaporative fraction (EF). This framework divides SM into different regimes separated by breakpoints, each dominated by different controls on L-A coupling^33,34^. First is a dry regime, in which SM is very low, such that little or no evapotranspiration (ET) occurs. Second is a transitional regime, where EF becomes sensitive to SM. In the transitional regime, high sensitivity indicates strong SM control over energy partitioning; as a result, small shifts in SM can significantly change EF and alter near-surface air temperature. Lastly, the wet regime marks where SM is high and evaporation is governed by incoming radiation (available energy), making EF essentially independent of SM. Collectively, these regimes determine how strongly SM constrains land evaporation and, therefore, the feedback to the atmosphere (i.e., the strength of land–atmosphere coupling). Regime shifts can alter the causality of energy and water availability toward ET, which may dampen or amplify L-A interactions^5^.

In recent decades, significant efforts have been made to quantify L-A coupling metrics using climate and weather prediction models as well as stand-alone land-surface models. Despite these efforts, there is a large discrepancy across Earth system models, as well as between coupled and uncoupled (land model only) simulations, in quantifying the type and strength of L-A coupling^35–41^. For instance, Koster *et al*.^18^ calculated coupling metrics between SM and precipitation from an ensemble mean of 12 models for boreal summer. The area-averaged value of this metric, when calculated by each individual model, varies widely. The greatest spread appears over semi-arid to semi-humid regions, often characterized as L-A coupling “hotspots,” where SM frequently lies in the transitional regime and exhibits strong variability. These findings indicate substantial inter-model differences. These differences have been consistently observed between various models used to study SM–HF coupling strength^42–44^, identified SM–EF regime distributions^45–47^, analyzed SM breakpoints^48^, and assessed SMM^49–53^. These inter-model inconsistencies can be attributed to imperfect representations of land-surface processes, unrealistic model parameters^44,54^, and uncertain atmospheric model parameterizations, leaving open the question: *Which model, if any, is correct?*

In addition to inter-model discrepancies in the representation of L-A interactions, challenges remain due to inconsistencies between model simulations and observational data^23,27,55^. For instance, model-based correlations between evaporation and SM are often stronger than observational estimates in water-limited regime^56–59^. Such discrepancies can cause inaccuracies in surface energy closure^59^, which can then lead to incorrect L-A feedback^58^ and greater uncertainty in air temperature forecasts^58,60–62^. Previous model-based assessments of evaporative regimes also reveal significant uncertainties in estimating tipping-point locations and timing associated with regime changes^2,15,17,33,63^. Studies of surface flux sensitivity to SM^15^ and global patterns of the soil moisture–latent energy (SM–LE) regime^45^ have also identified discrepancies among reanalysis and observation-based datasets. Dirmeyer *et al*.^64^ explored the sub-seasonal to seasonal progression of heatwaves and droughts, along with the diurnal evolution of tropospheric profiles over Europe, underscoring the contribution of the land surface to anomalous atmospheric conditions. Their findings from field data suggest that reanalyses may not fully capture the impact of drying soils on heatwaves. Building upon these studies, there is a need to quantify “true” L-A coupling, which could help assess model performance and potentially improve the physical representation of L-A processes.

One observational data source that can address this need is flux towers, which provide co-located measurements of land-surface states, surface fluxes, and near-surface atmospheric conditions. Although these observational data are valuable for validation purposes, their limited spatial^65–67^ and temporal coverage restricts their utility^68^. Meanwhile, satellite observations have continuous globally distributed coverage that can overcome these limitations. However, satellite datasets are prone to random observational errors^69,70^, caused by instrument limitations, signal interference, fluctuations in viewing angle, spatial re-processing, and inaccuracies in the retrieval algorithms used to convert radiances to state variables. Such errors can negatively impact estimates of L-A coupling^71^ and thus provide a challenge for the use of satellite measurements for numerical model evaluations.

Researchers have developed various techniques to detect and remove random errors. These include multi-source covariance-based methods such as the triple collocation (TC)^72^ and the extended double instrumental variable technique (EIVD)^73^, which are effective for estimating random error variances and true data correlations^56,69,74–78^. However, these methods have some limitations. For instance, TC requires the availability of three independent datasets, each with an assumed linear relationship to the true values being measured. These datasets should also have significant spatial and temporal overlap to reliably estimate errors^79^. Besides TC, an alternative approach matches the temporal power spectra of satellite products with *in-situ* observations through Fourier-transform time filtering^80^. Nonetheless, this method faces limitations, as continuous time series are needed for the Fourier transformation, while satellite records contain frequent gaps and missing values.

Thus, we still have an incomplete picture of the reality of global L-A coupling. Ideally, a global dataset of L-A coupling indices based on observations would provide an ideal independent benchmark for assessing the fidelity of weather and climate models. To fill this gap, we have produced global fields of L-A coupling metrics using a variety of observationally-based gridded datasets, accounting for random observational errors in the satellite-derived measurements. The method section explains the observational and *in-situ* measurements used in the study. Additionally, we outline the L-A coupling metrics and quantify the magnitude of stochastic errors in satellite SM time series in this section. This allows for a mathematical estimate of the SMM and Pearson correlation coefficient between SM and surface fluxes adjusted to account for random observational errors, and from these, we compute the terrestrial coupling using heat-flux standard deviations. Additionally, we adopt a simple conceptual framework to describe the dependence of EF on SM and to divide the SM range into three regimes. The data records section describes the dataset contents, with an overview of the file formats and folder structure. The technical validation section reports the analysis in terms of spatiotemporal consistency and performance, followed by a summary of this study.

## Methods

In this section, we first introduce the datasets used in this study, then explain the basic concepts related to deriving random observational errors. A brief section then follows, outlining the metrics used in this study, such as correlation, terrestrial coupling index, SMM, and SM regime. Lastly, we describe the main workflow used to produce global metrics datasets and the statistical significance testing employed.

### Input data

This study uses multiple global, gridded, observation-based datasets of SM and land-surface fluxes at approximately daily temporal resolution. Multiple datasets are used to provide a view of uncertainty that arises due to the choice of products. We also employ daily-mean *in-situ* measurements of surface SM and HF to validate the accuracy of observation-based metrics locally. Table 1 summarizes the datasets used in this study.

**Table 1:** Datasets used in this study.

Dataset	Variable(s)	Resolution	Period	Surface Soil Layer
SMAP L3 (SPL3SMP_E.006)	Soil Moisture	9 km × 9 km	April 2015-March 2025	Top 5 cm
CCI (V09.1)		0.25° × 0.25°	November 1978–2023	Top 5 cm
SoMo.ml		0.25° × 0.25°	2000–2019	0–10 cm
				10–30 cm
				30–50 cm
GLEAM v4.1a	Evaporation	0.1° × 0.1°	1980–2023	—
	Sensible Heat Flux			
CAMELE	Evapotranspiration	0.25° × 0.25°	1980–2022	—
FLUXCOM X-BASE	Evapotranspiration	0.25° × 0.25°	2001–2021	—
AmeriFlux	Soil Moisture	—	variable	Top 5 cm
	Latent Heat Flux			
	Sensible Heat Flux			

### *In-situ* measurements

AmeriFlux is a network of more than 660 flux tower sites across North, Central, and South America, managed by individual Principal Investigators (PIs) under the umbrella of FLUXNET^81^. At these sites, eddy-covariance towers continuously measure ecosystem fluxes and supporting meteorological variables across diverse ecosystems at high temporal (half-hourly) resolutions. These high-frequency measurements can be aggregated to daily values. For instance, the daily mean SM or a specific daily reading (such as 6:00 AM local time) can be derived from the continuous records.

Although the network follows common standard guidelines, sensors and calibration methods vary among sites; therefore, the AmeriFlux Management Project (AMP) applies a uniform workflow, including gap-filling, quality checks, and standardization, to create the BASE data product^82^. A key uncertainty of AmeriFlux data is spatial representativeness, because AmeriFlux measurements are point-based; a single sensor’s reading may not represent an entire site^83–85^. To address this issue, many sites install multiple sensors or report a spatial average, if available.

**Fig. 1:**
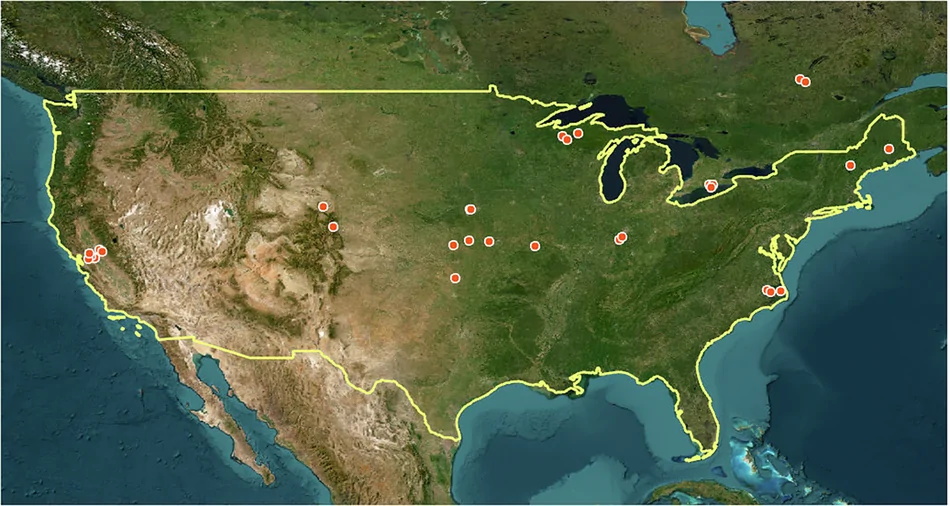
The location of AmeriFlux sites used in this study, with the contiguous United States outlined for reference.

Since AmeriFlux SM records provide a valuable benchmark for validation studies, as they capture real-world SM dynamics unaffected by satellite retrieval errors^81,86,87^, we compute L-A coupling metrics from the daily mean SM and HF from the AmeriFlux network to validate observation-based metrics. To ensure temporal consistency, we retain only those days from AmeriFlux that match the sampling interval of the satellite products. Figure 1 illustrates the locations of the sites employed for validation in this study; the 34 sites that qualify have sufficient temporal overlap with the period of the satellite products, an adequate sample size, completeness, and quality.

The AmeriFlux *in-situ* observations used in this study are obtained from the AmeriFlux data portal (https://ameriflux.lbl.gov) by identifying sites with the required variables (soil moisture/soil water content, latent heat flux, and sensible heat flux) and downloading the corresponding site-level AmeriFlux BASE products for the selected sites. General information for the AmeriFlux sites used in this study, including site names, abbreviations, latitudes, longitudes, record periods, and DOI identifiers, is provided in Supplementary Table 1.

### Soil moisture

#### SMAP

SMAP, the Soil Moisture Active Passive mission, launched in January 2015 and started collecting continuous data on March 31 of that year^88^. The SMAP satellite mission was initially planned to measure global surface SM (cm^3^/cm^3^) (approximately the top 5 cm of soil) on a ~9 km grid (EASE-Grid 2.0) for all land areas between roughly 85°S and 90°N with two distinct methods: active radar and passive radiometers^89,90^. However, in July 2015, the active radar component failed due to a power supply issue^89^. Despite this setback, the radiometer continued producing high-resolution data at ~9 km by leveraging the oversampling of the SMAP L-band radiometer (the native footprint is ~36 km) using the Backus-Gilbert optimal interpolation technique^90^, which converts the antenna measurements into gridded brightness temperatures at 9 km. Those L-band microwave brightness temperatures are then used to retrieve SM via a radiative-transfer (tau-omega) model^91^. The radiometer method for measuring SM is indirect and based on polarized microwave radiation at specific wavelengths, which have been found to correlate strongly with water content on Earth’s surface.

The orbit of SMAP repeats every eight days; in mid-latitudes, the data collection frequency is typically every 1–2 days because of the scanning patterns. SMAP’s orbit ensures global coverage every 2 – 3 days, but due to overlapping swaths and interpolation, nearly all land areas have higher frequency sampling. SMAP is on a Sun-synchronous orbit, scanning the Earth’s surface in ascending (roughly 6:00 PM overpass, local time) and descending (6:00 AM) modes. Although SMAP data have been calibrated and validated against *in-situ* measurements to ensure product quality^91^, uncertainties in the soil and vegetation temperature during the evening overpass can affect SM retrieval accuracy^92^. Therefore, we utilized SM retrieved from the SMAP L3 Version 006 (SPL3SMP_E, v6) morning overpass only. While these observations may not perfectly represent the daily mean SM, they provide a close approximation. In addition, since morning SM significantly impacts subsequent heat fluxes during the day, we create a stronger predictor for daytime heat fluxes by using SM measured at sunrise.

The SMAP L3 Version 006 (SPL3SMP_E, v6) is obtained from the NASA NSIDC DAAC/Earthdata portal: https://www.earthdata.nasa.gov/data/catalog/nsidc-cprd-spl3smp-e-006. The official dataset DOI is https://doi.org/10.5067/M20OXIZHY3RJ.

#### CCI

The Climate Change Initiative (CCI) soil moisture datasets are part of the European Space Agency (ESA) dataset production program, initiated in 2010 to provide continuous long-term global multi-satellite SM records^93–95^. There are three separate ESA CCI surface SM products: those derived from active (radar scatterometer) sensors, those derived from passive (radiometer) microwave sensors, and a COMBINED (active + passive) product. The COMBINED product is created by directly merging radiometer measurements in the L-, C-, X-, and K-bands from a wide range of satellite sources instruments, using the Land Parameter Retrieval Model (LPRM)^96–99^, together with C-band scatterometer observations processed with the TU Wien change detection method^100–102^. We use version 09.1 of the COMBINED product^93–95,103^, which represents daily volumetric moisture [m^3^ m^–3^] in the upper soil layer (~0–5 cm) spanning 45 years (November 1978–December 2023) at a 0.25° (~25 km) spatial resolution.

To create the CCI COMBINED soil moisture, all input data are first regridded to 0.25° and aligned in time (daily averages or instantaneous values at 00:00 UTC)^94^. Then, a series of bias-correction and scaling steps is performed. Each sensor’s data has its own climatology; therefore, a reference climatology has been used to normalize them. Specifically, in v09.1, the active-only product all scatterometer retrievals are rescaled into a common scatterometer climatology, while for the passive-only product, all radiometer retrievals are rescaled into a common radiometer climatology^104^. However, all sensors (active and passive) are rescaled to a common model-based climatology for the COMBINED product. This model reference is typically a land surface model or reanalysis, used to remove the systematic biases between sensors that can arise from different absolute calibration sensing depths.

This product applies a triple collocation analysis to estimate the random error variance of three independent datasets (active, passive, and model)^104^. These error estimates are then used in the merging step: CCI performs a weighted averaging of the active and passive contributions, where weights are inversely proportional to their error variances. In practice, this means that where the passive data is more reliable (lower error) than active, they receive higher weight, and vice versa. Finally, passive and active time series are merged into one by taking a weighted average on each day and pixel, producing the COMBINED product’s SM value. This merging methodology ensures that the strengths of active and passive sensors are utilized; specifically, active microwaves (C-band scatterometers) can be more accurate under moderate vegetation and have long-term stability, whereas passive microwaves (L-band radiometers, etc.) can measure absolute moisture more directly and penetrate deeper into arid soil. The CCI algorithm effectively balances these to provide an optimal estimate.

Despite the multi-platform nature of ESA CCI SM, the product still has multiple gaps across the world due to several limitations in the underlying retrieval models that it uses to convert raw satellite measurements (radiances, backscatter) into SM^105^. Data points corresponding to snow/frozen soils, dense vegetation such as tropical rainforests, associated with high uncertainties^106^, unsuccessful retrievals, and low signal-to-noise ratios, are excluded from the dataset^93^. However, barren ground is indicated in the dataset as a tag, meaning those observations are included but are marked rather than removed^105^.

ESA CCI Soil Moisture (COMBINED v09.1) is obtained from the official CEDA Archive / ESA Climate Change Initiative Soil Moisture repository: https://catalogue.ceda.ac.uk/uuid/0e346e1e1e164ac99c60098848537a29/. More information about this dataset can be found at https://climate.esa.int/en/. The official dataset DOI is https://doi.org/10.5285/0e346e1e1e164ac99c60098848537a29. The product guide is available at https://zenodo.org/records/13861012.

#### SoMo.ml

SoMo.ml (hereafter called simply SoMo) is a machine learning-based global surface and subsurface SM dataset covering the period 2000–2019, with daily resolution and a spatial resolution of 0.25°^107^. It provides SM in three layers: layer 1 (L1: 0–10 cm depth), layer 2 (L2: 10–30 cm), and layer 3 (L3: 30–50 cm). This dataset was produced by a Long Short-Term Memory (LSTM) neural network trained on *in-situ* SM measurements from over 1,000 stations worldwide. The LSTM model was fed with daily meteorological variables from ERA5 reanalysis and static features, including soil properties, land cover, elevation, etc. *In-situ* SM measurements, primarily from the International Soil Moisture Network (ISMN) and from Brazil’s Centro Nacional de Monitoramento e Alertas de Desastres Naturais (CEMADEN) observation network, to enhance tropical biome coverage, have been used as target variables for training the model. Since different sensors have different calibrations and represent different depths, scaling *in-situ* data to a reference model climatology was done to remove inconsistencies; therefore, the *in-situ* data’s mean and variance were adjusted to match ERA5 for the corresponding grid cell before training the model. The LSTM was trained separately for each layer, and the deeper-layer models use the moisture of the layer above as an input predictor. Finally, the SoMo dataset has been evaluated against observations, and cross-validation showed that the LSTM model reproduces SM dynamics at the stations with reasonable accuracy, especially for the temporal fluctuations, by capturing day-to-day and seasonal variations. Additionally, the comparison of LSTM’s performance with other products like ERA5, GLEAM, and ESA CCI shows that it performed well, especially in capturing temporal anomalies.

SoMo.ml is obtained from the Edmond repository of the Max Planck Institute for Biogeochemistry: https://doi.org/10.17871/BGI_SOMO.ML_V1_2020, and the DOI for this dataset is https://doi.org/10.17871/BGI_SOMO.ML_V1_2020.

### Land surface fluxes

#### FLUXCOM-X-BASE

FLUXCOM-X-BASE provides global fluxes, including CO₂ as GPP and NEE, and water vapor as transpiration and ET, covering the period from 2001 to 2021^108^. It is derived from FLUXCOM products, which use measurements from flux tower sites as ground truth for training machine learning models^109^ with a focus on the carbon cycle. Besides 294 flux tower sites, primarily from FLUXNET2015, ICOS (Europe), and AmeriFlux, FLUXCOM-X-BASE utilizes a set of predictor variables as inputs to the machine learning model^108^. These include ERA5 reanalysis data for meteorological variables and Moderate Resolution Imaging Spectroradiometer (MODIS) satellite data for vegetation indices and surface temperature. X-BASE relies on a single deterministic machine learning model called the extreme gradient-boosted tree (XGBoost), implemented as a gradient-boosted decision tree. The model was trained on half-hourly or hourly flux tower data paired with simultaneous predictor variables. In practice, the fluxes were aggregated to hourly resolution and matched with hourly ERA5 data and daily MODIS data interpolated to those hours.

Although the X-BASE products were generated based on the same principle as the original FLUXCOM ensemble, they demonstrate a much higher consistency with independent atmospheric carbon cycle constraints than the previous FLUXCOM versions^108^. This improvement probably results from two features of X-BASE. It ingests remote sensing and meteorological inputs in a single run rather than separate “RS-only” and “RS + meteo” runs, and it resolves sub-daily flux variations at hourly resolution. Therefore, X-BASE outputs hourly data on a 0.05° grid, which are then averaged into daily means on a 0.25° grid and monthly means on a 0.5° grid, while maintaining a monthly diurnal cycle at the native 0.05° resolution. This is a significant improvement, as previous FLUXCOM RS + METEO products were limited to monthly or, in some cases, 8-day resolutions^109^. X-BASE products rely solely on XGBoost rather than an ensemble of different regression algorithms, as used in the original FLUXCOM. Like CAMELE, sensible heat flux is not part of the FLUXCOM dataset. Hereafter, we will refer to the FLUXCOM-X-BASE product simply as FLUXCOM.

FLUXCOM X-BASE evapotranspiration is obtained from the ICOS data Portal: https://meta.icos-cp.eu/collections/_l85vWiIV81AifoxCkty50YI. Information about this dataset can be found on this website: https://www.icos-cp.eu/data-products/fluxcom-x-global-fluxes-collection, and the DOI for this dataset is https://doi.org/10.18160/5NZG-JMJE.

#### GLEAM

The GLEAM (Global Land Evaporation Amsterdam Model) dataset, developed initially at the University of Amsterdam in collaboration with multiple institutions, derives global evaporation and associated energy fluxes from multiple input sources^110^. To derive these fluxes, a range of key forcings is required. These include meteorological variables such as air temperature, precipitation, wind speed, vapor-pressure deficit; radiative inputs like incoming shortwave and net radiation; land surface attributes including land-cover fraction, soil properties, surface SM, and snow-water equivalent, carbon dioxide concentration, and vegetation characteristics such as leaf area index, vegetation optical depth, vegetation height, and the fraction of absorbed photosynthetically active radiation.

GLEAM products have two versions. Version (a) estimates evaporation from a combination of *in-situ* measurements, reanalysis products, and satellite observations. Version (b), by contrast, drives the Penman–Monteith equation exclusively with observation-based inputs, including *in-situ* and satellite measurements, to compute potential evaporation (Ep). Aside from the drivers for net radiation, incoming shortwave radiation, air temperature, precipitation, and vapor-pressure deficit, the two versions share the same input sources.

In this study, surface heat fluxes, including evaporation and sensible heat flux, are used from GLEAM v4.1a^111^, which provides daily data at a spatial resolution of 0.1°. Earlier versions of GLEAM relied on the Priestley–Taylor formulation to estimate potential evaporation^110^, while version 4 employs the Penman-Monteith^111^. In this version, actual evaporation (E) is obtained by multiplying potential evaporation by an evaporative-stress factor, E = S × Ep. The stress factor (0 ≤ S ≤ 1), which represents moisture constraints, is predicted by a hybrid machine-learning model, a deep neural network trained on global flux tower ET and sap flow data. The stress factor is computed separately for tall-vegetation (transpiration) and bare-soil components, resulting in transpiration and bare-soil evaporation estimates. In addition to all evaporation components, GLEAM v4 derives sensible heat flux from the surface-energy balance: after calculating latent-heat flux (λE), the remaining available energy (Rn; – G – λE) is assigned to sensible heat (H), ensuring energy conservation at the land surface, where Rn; is net radiation and G is ground heat flux. Thus, GLEAM data allow for the estimation of daily EF.

GLEAM v4.1a is obtained from the official GLEAM distribution site: https://www.gleam.eu. In addition, this dataset is available on Zenodo at https://zenodo.org/records/14056080.

#### CAMELE

The Collocation-Analyzed Multi-source Ensembled Land Evapotranspiration (CAMELE) dataset offers long-term global ET estimates at 0.1° (2000–2020) and 0.25° (1980–2022) resolution^112^. It uses a collocation-based method that considers non-zero error cross-correlation when combining multi-source data. For this study, we utilize the CAMELE product at 0.25° spatial resolution and daily temporal resolution. This product was generated from ERA5-Land, an older version of GLEAM (v3.7a), and Global Land Data Assimilation System (GLDAS) versions 2.0 and 2.1 using the extended double instrumental variable technique (EIVD). This advanced collocation technique estimates the random error variance of each input dataset and the cross-correlation among their errors^73^. By assigning inverse-variance weights to individual datasets, it minimizes overall errors while explicitly accounting for non-zero error cross-correlations. As a result, datasets with higher estimated random error or errors correlated with others receive lower weights, producing a more accurate long-term product. The collocation method also excludes periods or regions with insufficient data from the weighting process. In addition, extreme outliers (e.g., a day where one model produced an unreasonably high ET) are mitigated by the weighting. That model would have a significant error variance and thus a low weight. The CAMELE product has been rigorously validated against ground observations across various ecosystems^112^. However, it does not provide estimates of other surface heat flux variables.

The CAMELE products are available at https://zenodo.org/records/8047038, and the DOI for this dataset is https://doi.org/10.5281/zenodo.8047038.

### Random observational error

Hasselmann^113^ used first-order Markov processes to study the relationship between white noise, representing short-term variability, and the red noise spectra in sea surface temperatures. Later, Delworth and Manabe^114^, and subsequently, Vinnikov and Yeserkepova^115^, applied the method to SM anomalies. They demonstrated that SM time series behave like first-order Markov processes, indicating that the temporal variability of SM follows a red noise statistical model. In simpler terms, the concept of first-order Markov processes originates from the assumption that the surface water budget can be described by a basic differential equation in time:

1 $$\frac{{dS}\left(t\right)}{{dt}}=P\left(t\right)-{ET}\left(S\left(t\right)\right)-R\left(S\left(t\right)\right)$$

Where S represents soil moisture at time t, P(t) is the precipitation rate, and both evapotranspiration (ET) and runoff (R) are functions of SM. Then, Delworth and Manabe^114^ formulated Eq. (1) as a first-order ordinary differential equation, generalized as:

2 $$\frac{dW(t)}{dt}=-\lambda W(t)+\omega (t)$$

Where ω(t) represents a white noise time series acting as a random forcing on the soil moisture W(t), the resulting time series for W(t) will exhibit a red noise spectrum. The term λ is a constant and is defined as $\lambda ={E}_{P}/{W}_{{FC}}$, where ${E}_{p}$ and ${W}_{{FC}}$ represent potential evaporation and field capacity, respectively. The term λ represents frequency, and $\tau =1/\lambda$ denotes the decay timescale of the autocorrelation function (ACF) of SM. Therefore, the ACF can be represented as:

3 $$r\left(\tau \right)=\exp \left(-\lambda \tau \right)$$

This suggests that the ACF of SM decreases exponentially with increasing lag. Given this, it logically follows that at time lag zero (τ=0), the correlation is expected to be 1. Nonetheless, when the natural logarithm of this autocorrelation function (ACF) is graphed against time, the fitted line extrapolated to τ=0 typically intersects at a correlation value < 1^116^, indicating the presence of random error in the time series. This deviation is useful for quantifying the magnitude of random error in the measurements, as formulated by Vinnikov *et al*.^117^:

4 $$\frac{{\delta }^{2}}{{\sigma }^{2}}=\frac{a}{\left(1+a\right)}$$

Here, a represents the value of $(1-\mathrm{ln}(d))$ at τ=0 where d signifies the displacement below correlation 1 at τ=0. The term $\frac{{\delta }^{2}}{{\sigma }^{2}}$ denotes the ratio of SM observational error variance to the temporal variance of SM measurements. Using this definition, for discrete lags of τ (days), valid $\left({X}_{t},{X}_{t+\tau }\right)$ pairs across segments (for MJJAS, NDJFM, and all seasons) are collected, and any pair containing missing values is omitted. Then, ACF can be calculated:

5 $$ACF=r({X}_{t},{X}_{t+\tau })=\frac{\mathop{\sum }\limits_{t=0}^{M-\tau }({X}_{t+\tau }-\bar{{X}_{t+\tau }})({X}_{t}-\bar{{X}_{t}})}{\sqrt{\sum {({X}_{t}-\bar{{X}_{t}})}^{2}\ast \sum {({X}_{t+\tau }-\bar{{X}_{t+\tau }})}^{2}}}$$

Here, M represents the sample size for each segment. To obtain robust estimates for the displacement d, ordinary least squares regression is performed on the logarithm of the ACF for window lengths ranging from two to seven days (lags 1–2, 1–3, …, 1–7). Then, by taking the average of displacements $\bar{d}$ (preventing non-physical results by avoiding d < 0), and estimating a as ($\alpha =1-{e}^{\bar{d}}$), we quantify the variance of observational random error (Eq. (4)).

It should be noted that the missing values are not gap-filled or interpolated. Instead, for each lag, the autocorrelation is computed using only valid observation pairs in which both values are available. This pairwise approach is applied separately within each year, and grid cells with insufficient valid data for fitting are excluded. Because the lag calculation is based on index position, this implementation assumes that the input data are already harmonized to a common regular temporal sampling interval.

To assess the robustness of the estimated variance of random observational error, a sensitivity test is performed in which the log-linear regression of the ACF is repeated using multiple short-lag windows (e.g., lags 1–2, 1–3, …, 1–7) for the SMAP time series at the ARM site (97.52°W, 36.59°N) during June-August (JJA). For each fitting window, the fitted straight line is extrapolated to τ=0 to estimate the intercept and the corresponding displacement *d*, from which α is calculated and subsequently used to estimate the variance of observational random error according to Eq. (4). Supplementary Fig. 1a shows the fitted straight lines for different lags as well as the observed logarithm of the ACF values and the extrapolated intercepts at τ=0. Supplementary Fig. 1b shows the standard error of the fitted intercept for each lag-window choice. According to this figure, although the uncertainty for the shortest fitting windows is not the smallest, the resulting intercept estimates are broadly similar across the tested lag windows, indicating that the estimated displacement and the corresponding variance of random observational error are stable with respect to the choice of fitting window.

The Markovian error-adjustment framework relies on several assumptions regarding both the SM signal and the measurement error and therefore has important limitations. One key assumption is stationarity. Specifically, the framework assumes that the true SM anomaly, $\left({W}_{t}\right)$, follows a stationary first-order Markov process with exponential memory. Under weak stationarity, the mean and variance of $\left({W}_{t}\right)$ are constant over time, and the autocovariance depends only on lag rather than calendar time. After anomaly calculation, the mean of $\left({W}_{t}\right)$ is typically assumed to be approximately zero. Under these conditions, the ACF takes the exponential form given in Eq. (3). Accordingly, the application of this framework implicitly assumes that seasonality has been removed, long-term trends are absent or negligible, and the decay parameter $\left(\lambda \right)$ in Eq. (3) remains approximately constant over the analysis period. If the SM time series exhibits drift, trend, or substantial time-varying variance, the first-order Markov or red-noise assumption may no longer be valid.

In addition to the stationarity assumption, the framework relies on independence assumptions of the two noise terms. The first is the process-forcing term, $\omega \left(t\right)$, which is typically assumed to be white noise with zero mean and no temporal autocorrelation, and to be uncorrelated with past states. By contrast, the state variable ${W}_{t}$ is temporally correlated since it has memory (red noise); only the forcing increment is assumed to be white. The second noise term is the measurement error, $\left({\varepsilon }_{t}\right)$, which is typically assumed to have zero mean, constant variance $\left({\delta }^{2}\right)$, no temporal autocorrelation, and no dependence on the true signal. Consequently, both the forcing and measurement errors are not temporally correlated.

The Markovian framework used to estimate random error in this study has some limitations. In particular, the framework assumes that the process-forcing term is temporally uncorrelated (white noise) and therefore neglects persistence in meteorological forcing such as precipitation and radiation. This persistence in forcing, as well as seasonal variability and land–atmosphere feedback, may either extend or shorten SMM. Under such conditions, part of the observed autocorrelation may reflect persistence in the forcing rather than the intrinsic memory of the soil moisture state itself, which may in turn affect the estimated SMM and random-error magnitude.

### Metrics

In this study, we use global gridded observational datasets to calculate metrics. Because these datasets have different spatial resolutions and time spans, we perform all analyses over the aligned time periods. Additionally, if there is a comparison between metrics, the analysis is conducted at a common spatial resolution of 0.25° × 0.25°; otherwise, SMAP-GLEAM and SMAP-only combination metrics are at 0.1° × 0.1° resolution. Table 2 summarizes the observational data sources used in this study to generate the metrics, along with their corresponding time-aligned periods.

**Table 2:** Overview of the time alignment of data for metrics calculated in this study.

Metrics	Variables	Flux Data	Soil Moisture Data	Period
Coupling: Correlation & Terrestrial coupling index	SM–E	CAMELE	SMAP	April 2015-March 2022
			CCI	1980–2022
			SoMo-L1	2000–2019
		FLUXCOM	SMAP	April 2015- March 2021
			CCI	2001–2019
			SoMo-L1	2001–2019
		GLEAM	SMAP	April 2015- March 2023
			CCI	1980–2023
			SoMo-L1	2000–2019
	SM–H	GLEAM	SMAP	April 2015- March 2023
			CCI	1980–2023
			SoMo-L1	2000–2019
	SM–EF	GLEAM	SMAP	April 2015- March 2023
			CCI	1980–2023
			SoMo-L1	2000–2019
SMM	SM	—	SMAP	April 2015- March 2025
			CCI	1971–2023
			SoMo-L1	2000–2019
Regime	SM–EF	GLEAM	SMAP	April 2015-March 2023
			CCI	1980–2023
			SoMo-L1	2000–2019
			SoMo-L2	2000–2019
			SoMo-L3	2000–2019

### Adjusted correlation

One of the most widely used metrics in climate science for measuring the relationship between two variables is Pearson’s correlation. For a pair of random variables $(X,{Y})$, the correlation coefficient is calculated as the covariance of X and Y, divided by the product of their standard deviations:

6 $${\rho }_{X,Y}=\frac{cov\left(X,Y\right)}{{\sigma }_{X}{\sigma }_{Y}}$$

Where ${\rm{c}}{\rm{o}}{\rm{v}}(X,Y)$ is the covariance, while ${{\rm{\sigma }}}_{{\rm{X}}}$ and σ_Y_ are the standard deviations of X and Y, respectively. Pearson’s correlation is constrained to the range ± 1.

As discussed previously, since the random errors of SM follow a pattern similar to white noise and have a normal probability distribution function (PDF) characterized by ${\rm{N}}(0,\,{\delta }^{2})$, the correlation formula can directly incorporate the impact of such errors when they occur in satellite-based SM data:

7 $${R}_{{Adjusted}}=R\left[{{SM}}_{T},{HF}\right]=\frac{{cov}\left[{{SM}}_{T},{HF}\right]}{\sqrt{{var}\left[{{SM}}_{T}\right]\cdot {var}\left[{HF}\right]}}$$

Where ${{SM}}_{T}$ signifies a hypothetical “true” SM time series, i.e., with the effects of random error removed. Nonetheless, the original observed SM ($S{M}_{O}$) data are affected by random errors, represented as:

8 $${{SM}}_{O}={{SM}}_{T}+\varepsilon $$

9 $${{SM}}_{T}={{SM}}_{O}-\varepsilon $$

Where ${\rm{\varepsilon }}$ symbolizes random observational error, characterized by a normal distribution ${\rm{N}}(0,\,{\delta }^{2})$, where ${\delta }^{2}$ can be estimated from the relationship described in the random observational error section, consequently, the adjusted correlation is reformulated based on $S{M}_{O}$:

10 $${R}_{{Adjusted}}=\frac{{cov}\left[{{SM}}_{O}-\varepsilon ,{HF}\right]}{\sqrt{{var}\left[{{SM}}_{O}-\varepsilon \right]\cdot {var}\left[{HF}\right]}}$$

The variance of $S{M}_{T}$ can be expressed as:

11 $${var}\left[{{SM}}_{T}\right]={var}\left[{{SM}}_{O}-\varepsilon \right]$$

Assuming the observational errors ε are independent of the true signal, the variance of the observed SM is:

12 $${var}\left[{{SM}}_{O}\right]={var}\left[{{SM}}_{T}\right]+{var}\left[\varepsilon \right]={var}\left[{{SM}}_{T}\right]+{\delta }^{2}$$

Therefore,

13 $${var}\left[{{SM}}_{O}-\varepsilon \right]={var}\left[{{SM}}_{T}\right]={var}\left[{{SM}}_{O}\right]-{\delta }^{2}$$

Moreover, we ascertain that:

14 $${cov}\left[{{SM}}_{O}-\varepsilon ,{HF}\right]={cov}\left[{{SM}}_{O},{HF}\right]-{cov}\left[\varepsilon ,{HF}\right]={cov}\left[{{SM}}_{O},{HF}\right]$$

Thus, the correlation that would result from SM data without random error can be estimated:

15 $${R}_{{Adjusted}}=\frac{{cov}\left[{{SM}}_{O},{HF}\right]}{\sqrt{\left({var}\left[{{SM}}_{O}\right]-{\delta }^{2}\right)\cdot {var}\left[{HF}\right]}}$$

While,

16 $${R}_{{Original}}=\frac{{cov}\left[{{SM}}_{O},{HF}\right]}{\sqrt{\left({var}\left[{{SM}}_{O}\right]\cdot {var}\left[{HF}\right]\right)}}$$

Thus, $$|{R}_{Adjusted}|\ge |{R}_{Original}|$$.

### Adjusted terrestrial coupling index

The terrestrial coupling index combines measures of correlation and variability into a single metric^15^ and is defined as a correlation-based metric. Specifically, this metric indicates how strongly the response (HF) is coupled to the forcing (SM*)*. The terrestrial coupling index $I({SM},{HF})$ can be defined as:

17 $$I\left({SM},{HF}\right)=\frac{{cov}\left({SM},{HF}\right)}{{\sigma }_{{SM}}}={\rho }_{{SM},{HF}}{\sigma }_{{HF}}$$

Therefore, the adjusted terrestrial coupling index, accounting for the effect of random error, can be estimated by substituting the adjusted correlation from Eq. (15) into Eq. (17):

18 $${I}_{{Adjusted}}\left({SM},{HF}\right)={R}_{{Adjusted}}\ast {\sigma }_{{HF}}$$

While,

19 $${I}_{{Original}}\left({SM},{HF}\right)={R}_{{Original}}\ast {\sigma }_{{HF}}$$

### Adjusted soil moisture memory

Since random observational errors lead to an underestimation of SMM, we use the adopted Markovian framework to estimate SMM adjusted for the effect of these errors. In this framework, SMM is defined as the time lag at which the SM time series autocorrelation is reduced to 1/e, the e-folding time scale^114^. In other words, SMM is the lag at which a linear extrapolation through the log-autocorrelation drops to –1. After finding the displacement d associated with (Eq. (4)), we may shift this extrapolated line by d to start at zero and can identify the lag at which the adjusted log (ACF)= –1, ameliorating the effect of random observational error on estimated SMM^71,115–118^. To estimate the adjusted SMM, we first identify valid data pairs to avoid missing values in the calculations. After finding the displacement (d) of the best-fitted line to the log (ACF) through lags 1 to 7, with $y=\log ({ACF})$, the adjusted SMM can be calculated as:

20 $${SMM}_{Adjusted}=\{\begin{array}{cc}\frac{-1-d}{slope} & if\,d > 0\\ \frac{-1}{slope} & if\,d < 0\end{array}$$

### Soil moisture regime distribution

**Fig. 2:**
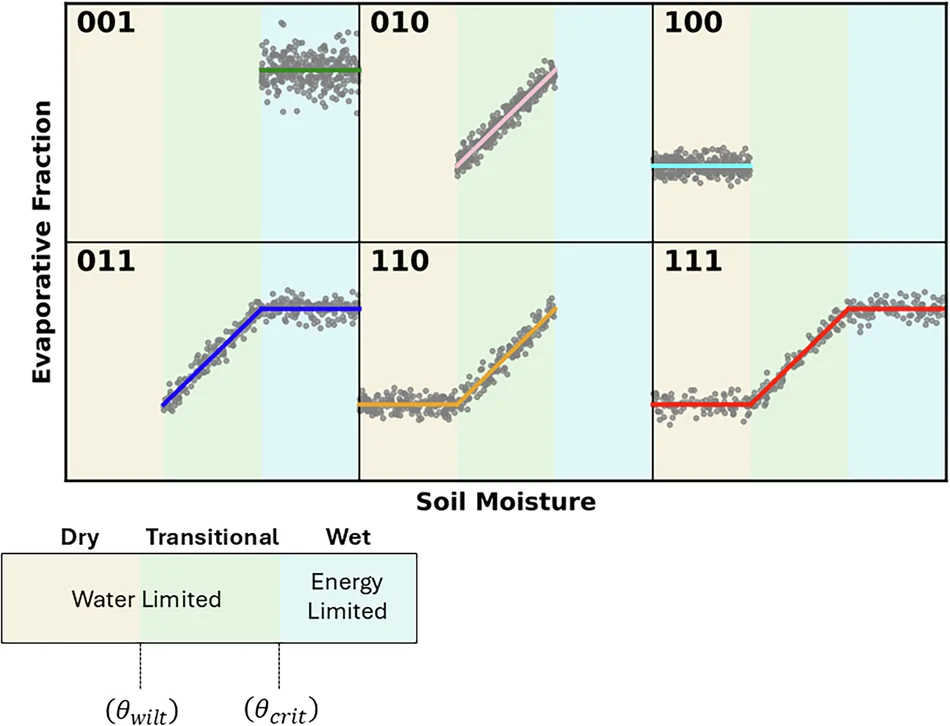
Six candidate segmented-regression models for the SM-EF relationship, identified by regime-inclusion codes (dry, transitional, wet): dry only (100), transitional only (010), wet only (001), dry + transitional (110), transitional + wet (011), and dry + transitional + wet (111). The wilting point and critical soil moisture are marked as ${\theta }_{{wilt}}$ and ${\theta }_{{crit}}$, representing regime transition points.

The approach for soil moisture regime identification follows the optimum piecewise linear regression approach proposed by Schwingshackl *et al*.^63^ and modified by Hsu and Dirmeyer^45^. It involves fitting six potential segmented regression models of EF as a function of SM at each grid cell. To simplify notation, we label each segment of these regression models with a three-digit binary number that indicates the absence (0) or presence (1) of each regime, respectively. The first digit corresponds to the presence or absence of the dry regime, the second digit to the presence or absence of the transitional regime, and the third digit to the presence or absence of the wet regime. Figure 2 displays schematic diagrams of the six segmented-regression models used in this study to fit the SM-EF datasets. Once these models are fitted, the SM range can be divided into as many as three different regimes: dry, transitional, or wet, separated by breakpoints indicating the intersection points between regression lines. A breakpoint found between the dry and transitional regimes is deemed the wilting point ${\theta }_{{wilt}}$, and the breakpoint between the transitional and wet regimes is the critical SM ${\theta }_{{crit}}$, representing the boundary between water and energy limitations on E.

According to Fig. 2, candidate 001 (wet only) and candidate 100 (dry only) are represented as horizontal plateaus of EF, whereas candidate 010 (transitional only) is represented as a positive-slope linear segment. For the candidate 111 (dry + transitional + wet), EF is constant for ${SM} < {\theta }_{{wilt}}$, increases linearly for ${\theta }_{{wilt}}\le {SM} < {\theta }_{{crit}}$, and reaches a constant wet-regime plateau for ${SM}\ge {\theta }_{{crit}}$. Candidates 110 (dry + transitional) and 011 (transitional + wet) omit the wet and dry regimes, respectively. If θ denotes soil moisture and ${EF}\left(\theta \right)$ the corresponding evaporative fraction, the fitted candidate models are:

$$001:{EF}\left(\theta \right)=c$$

$$010:{EF}\left(\theta \right)={\beta }_{0}+{\beta }_{1}\theta $$

$$110:{EF}(\theta )=\left\{\begin{array}{lc}0, & \theta  < {\theta }_{{wilt}}\\ {\alpha }_{1}(\theta -{\theta }_{{wilt}}), & \theta \ge {\theta }_{{wilt}}\end{array}\right.$$

$$011:{EF}\left(\theta \right)=\left\{\begin{array}{cc}{\alpha }_{1}\left(\theta -{\theta }_{{wilt}}\right),\, & \theta  < {\theta }_{{crit}}\\ {\alpha }_{1}\left({\theta }_{{crit}}-{\theta }_{{wilt}}\right), & \theta \ge {\theta }_{{crit}}\end{array}\right.$$

$$111:{EF}(\theta )=\left\{\begin{array}{ll}0, & \theta  < {\theta }_{{wilt}}\\ {\alpha }_{1}(\theta -{\theta }_{{wilt}}), & {\theta }_{{wilt}}\le \theta  < {\theta }_{{crit}}\\ {\alpha }_{1}({\theta }_{{crit}}-{\theta }_{{wilt}}), & \theta \ge {\theta }_{{crit}}\end{array}\right.$$

With

21 $${\theta }_{{crit}}={\theta }_{{wilt}}+\delta ,\,\delta  > 0$$

Where c is a constant EF value representing a horizontal plateau. In candidate 001, c is the mean EF across all samples at that grid cell, while in candidate 111, the wet-regime plateau is ${\alpha }_{1}\left({\theta }_{{crit}}-{\theta }_{{wilt}}\right)$. ${\beta }_{0}$ and ${\beta }_{1}$ are the intercept and positive slope of the candidate 010, ${\alpha }_{1}$ is the positive slope of the transitional segment in the piecewise candidates 110, 011, and 111; it represents the sensitivity of EF to soil moisture within the transitional regime. The positive distance between two breakpoints is δ guaranteed ${\theta }_{{wilt}} < {\theta }_{{crit}}$.

To ensure robust model selection, we impose several physical and statistical constraints:

- Data availability: At each grid cell, at least 10% of the time-series length (N_min_) must be available for fitting.
- Outlier handling: If 50% or more of the remaining records share the single maximum SM value (usually saturated, e.g., wetland), those maximum-value points are removed; the grid cell is excluded from further analysis if the reduced sample falls below N_min_.
- Breakpoint bounds: For candidates 010, 110, 011, and 111, the breakpoint ${\theta }_{{wilt}}$ must lie between 0.01 and 0.30 m^3^ m^–3^.
- Sample size: Each identified segment must contain at least 10 data points before being included for fitting.
- Note that the breakpoint values of SM are identified where two segments intersect. Wilting point may be extrapolated when the transitional regime is identified, but the dry regime is not. For candidates 100 and 001, neither breakpoint can be quantified.

Among all candidate models that meet the imposed criteria and constraints, we select the one with the lowest finite Bayesian Information Criterion (BIC)^119^ as the most likely SM-EF model candidate. Specifically, the classical least-squares form of the BIC is used:

22 $${BIC}=N\,ln\left(\frac{{RSS}}{N}\right)+k\,lnN$$

23 $${RSS}=\mathop{\sum }\limits_{i=1}^{N}{\left({y}_{i}-{\hat{y}}_{i}\right)}^{2}$$

Where N is the number of valid SM-EF pairs at a given grid cell, RSS is the residual sum of squares of the model, and k denotes the number of free parameters in the model (e.g., k = 1 for 001 and 100; *k* = 2 for models 010 and 110; and k = 3 for models 011 and 111). For simplicity, hereafter, the most likely SM-EF model will be referred to as the best model.

If the BIC selects the flat model (no transitional regime) as the best fit, an additional screening is conducted to determine whether the grid cell should be classified as the dry-only candidate 100 or wet-only candidate 001. If at least 50% of the valid values at a cell fall below product-specific thresholds for both SM and EF, the cell is labeled 100. The thresholds are listed in Table 3.

**Table 3:** Thresholds for reclassifying candidate 001 as candidate 100.

Product	EF threshold	SM threshold
SMAP-GLEAM SoMo(L1)-GLEAM	≤0.20	≤0.20
CCI-GLEAM	≤0.20	≤0.10
SoMo(L2)-GLEAM	≤0.10	≤0.25
SoMo(L3)-GLEAM	≤0.10	≤0.20

After identifying the best candidate model based on BIC, some of these models can provide us with breakpoints, while others cannot. This raises the question: how do we determine actual breakpoint values when the best model does not provide them? To answer this question, we should consider that ${\theta }_{{wilt}}$ may be estimated by extrapolation for both candidates 010 and 011 because there is no dry regime in these two models, whereas candidates 111 and 110 use a segmented regression model to determine ${\theta }_{{wilt}}$. Additionally, since the consistency between ${\theta }_{{wilt}}$ and ${\theta }_{{crit}}$ is very important for those initializing forecasts, we prioritize that both of these breakpoints ideally come from the same model. Breakpoint selection scenarios are illustrated in Supplementary Fig. 2 and are described below:

1. If the best model candidate is 111, both ${\theta }_{{wilt}}\_111$ and ${\theta }_{{crit}}\_111$ are implemented.
2. If the second-best model is 111 and the BIC difference between the first- and second-best models ($\left|\triangle {BIC}\right|)$ is within acceptable thresholds (≤50 for ${\theta }_{{wilt}}$, ≤10 for ${\theta }_{{crit}}$), then both ${\theta }_{{wilt}}\_111$ and ${\theta }_{{crit}}\_111$ are selected. The justification for these thresholds is explained in the breakpoint uncertainty and selection sensitivity section.
3. If the best model candidate is 011, both ${\theta }_{{wilt}}\_011$ and ${\theta }_{{crit}}\_011$ are used.
4. If candidate 011 ranks as the second-best model and meets the $\left|\triangle {BIC}\right|$ criteria (≤50 for ${\theta }_{{wilt}}$, ≤10 for ${\theta }_{{crit}}$), then both ${\theta }_{{wilt}}\_011$ and ${\theta }_{{crit}}\_011$ are selected.
5. If the best model candidate is 110, ${\theta }_{{wilt}}\_110$ is used.
6. If the second-best model is candidate 110 with an acceptable $\left|\triangle {BIC}\right|$ threshold (≤50 for ${\theta }_{{wilt}}$), ${\theta }_{{wilt}}\_110$ is applied.
7. If the best model is candidate 010, ${\theta }_{{wilt}}\_010$ is selected.
8. If the second-best model is candidate 010 with an acceptable $\left|\triangle {BIC}\right|$ threshold (≤50 for ${\theta }_{{wilt}}$), ${\theta }_{{wilt}}\_010$ is used.

Note that SM breakpoints (${\theta }_{{wilt}}$, ${\theta }_{{crit}}$) are diagnostic quantities inferred from the fitted SM–EF relationship. They should therefore be interpreted as empirical thresholds in ecosystem-scale evaporative behavior, rather than as direct estimates of intrinsic soil-hydraulic thresholds. In other words, (${\theta }_{{wilt}}$) is the SM value at which the observed EF response changes behavior in the segmented regression. That does not ensure it is identical to the classical soil-physics permanent wilting point, which is usually defined from plant/soil hydraulic properties. Likewise, (${\theta }_{{crit}}$) is a diagnostic threshold marking the transition from moisture-limited to energy-limited EF behavior, not a universal constant. This distinction matters because these breakpoints depend on the SM and EF products, time period, fitting assumptions, vegetation/climate controls, and model structure, not only on soil texture or plant physiology.

In summary, quality control (QC) for the selection of the most likely segmented-regression candidate and the associated SM breakpoints (${\theta }_{{wilt}}$, ${\theta }_{{crit}}$) is applied both before and after fitting, as follows:

- **Quality control before fitting:** These are the checks applied before any candidate model is accepted:

1. Remove missing or invalid paired data: At each grid cell, only paired SM–EF observations with finite values are kept.
2. Require a minimum sample size: A grid cell is analyzed only if the number of valid observations is at least 10% of the time-series length. If fewer observations exist, the cell is rejected.
3. Removing the outlier SM values: If the fraction of the observations equal to the maximum SM value is at least 50%, indicative of seasonal or permanent wetland, all observations at that maximum SM value are removed, and the sample size is checked again. If too few data remain afterward, the cell is rejected. This is a pre-fitting QC step to reduce distortion from excessive accumulation at the upper SM bound.

b. **Quality control after fitting:** These are the checks applied after a model has been fitted, to decide whether that fit is acceptable:

1. Select the best model candidate with valid statistics: Model selection is only based on the finite BIC values. For each candidate, RSS and BIC are computed only if RSS is positive and finite; otherwise, the candidate is rejected. If any candidate fails to converge or yields a non-finite BIC, the grid cell remains unclassified, and breakpoints (${\theta }_{{wilt}}$, ${\theta }_{{crit}}$) are reported as missing values. The candidate with the lowest finite BIC is selected as the most likely model. AIC and cross-validation are not used in this implementation.
2. Require enough points in each segment: Each identified segment must have at least 10 observations to be accepted.
3. Apply parameter constraints through bounded fitting: For candidates 110, 011, and 111, the fitting bounds enforce non-negative slope and ordered breakpoints through δ; therefore, candidates violating those constraints are not retained.
4. Reject fits with implausible ${\theta }_{{wilt}}$: For candidates 010, 110, 011, and 111, the fitted or inferred ${\theta }_{{wilt}}$ must satisfy $0.01\le {\theta }_{{wilt}}\le 0.30\left[{m}^{3}{m}^{-3}\right]$.
5. Dry-only reclassification (100): After BIC selection, if candidate 001 is best and at least 50% of the valid values at a cell fall below product-specific thresholds listed in Table 3, the cell is reassigned to class 100.
6. Breakpoint screening through hierarchical selection: Final breakpoint estimates are chosen using a hierarchical $\left|\triangle {BIC}\right|$ rule between the first- and second-best models rather than directly from the best candidate model. If candidate 111 is the best-BIC model, both ${\theta }_{{wilt}}$ and ${\theta }_{{crit}}$ are taken from 111. If candidate 111 is the second-best model, its ${\theta }_{{wilt}}$ and ${\theta }_{{crit}}$ are still accepted when $\left|\triangle {BIC}\right|\le 50$ and $\left|\triangle {BIC}\right|\le 10$, respectively. If no acceptable 111 estimate is available, the same logic is then applied to candidate 011. If no acceptable candidates 111 or 011 estimate are available for ${\theta }_{{wilt}}$, candidate 110 is used next, followed by 010, with second-best acceptance requiring $\left|\triangle {BIC}\right|\le 50$. Thus, ${\theta }_{{crit}}$ could be reported only from acceptable 111 or 011 candidates, whereas ${\theta }_{{wilt}}$ could also be inferred from 110 or 010.

### Workflow

To compute the adjusted correlation-based metrics, we obtain anomalies of the variables and remove their seasonal cycles so that no interannual variability or trends influence the calculations and statistics. To determine the seasonal cycle of daily time series, a sum of sine and cosine functions is fitted to the time series of each variable, incorporating three annual harmonics after detrending. Once the seasonal component is modeled, it is subtracted from the original time series. The annual climatological cycle was not removed for the SM-EF regime calculations.

To reduce the effects of frozen soil, snow cover, and spurious signals when downwelling radiation is weak and EF values are erratic, we analyze the daily time series during warm seasons for three regions: May through September (MJJAS) for 23°N-60°N, November through March (NDJFM) for 23°S-60°S, and all months for 23°S-23°N. Regions poleward of 60° latitude are not included in this analysis due to an abundance of quality concerns.

All calculations of adjusted metrics have been performed at a common spatial resolution of 0.25°, except for combinations derived from SMAP and GLEAM—such as ${R}_{{Adjusted}}$, ${I}_{{Adjusted}}$, SM-EF regime, and adjusted SMM—which have been processed at 0.1°. Nevertheless, uncertainty analyses between all combinations have been conducted at a common spatial resolution of 0.25°. The regridding is accomplished using bilinear interpolation.

To compare adjusted metrics with those calculated from AmeriFlux measurements, the nearest grid points of each observational dataset to each AmeriFlux site have been selected. Then, to account for frozen days in the *in-situ* SM time series, any days when the *in-situ* soil temperature fell below 0°C are excluded. All preprocessing steps are then repeated for the *in-situ* time series, including obtaining anomalies, removing the seasonal cycle, and selecting only warm-season months. However, we find *in-situ* measurements exhibit much smaller observational random error than remote sensing retrievals; we have not applied the random-error correction to AmeriFlux data.

A concise summary of key L-A metrics calculated in this study, including SM memory, SM regimes, SM breakpoints, correlation, and the terrestrial coupling index, with formulas, units, expected signs, and physical interpretations for the SM–H, SM–LE, and SM–EF relationships is listed in Table 4.

**Table 4:** Overview of land-atmosphere metrics calculated in this study.

Metric	Formula	Variable pair(s)	Units	Sign	Physical interpretation
**Correlation**	Eq. (15)	SM–E	none	−, 0, +	Direction and strength of linear co-variability between SM and E; positive values indicate that wetter soils are generally associated with greater evapotranspiration, consistent with stronger moisture influence on E in water-limited or transitional regimes. Negative values indicate that higher SM tends to coincide with lower E, often when atmospheric or radiative controls (e.g., cloudy or rainy conditions) dominate the covariance and mask or outweigh SM control.
		SM–EF			Direction and strength of linear co-variability between SM and EF; positive values indicate that wetter soils are associated with a larger EF, meaning a greater share of available energy is partitioned into latent heat rather than sensible heat. Negative values indicate that wetter soils are associated with lower EF, typically when radiative or atmospheric forcing dominates the covariance.
		SM–H			Direction and strength of linear co-variability between SM and H; negative values indicate that wetter soils are generally associated with lower sensible heat flux, consistent with stronger partitioning of available energy into latent heat rather than sensible heating. Positive values indicate that higher SM coincides with higher H, typically when atmospheric or radiative forcing dominates the covariance and masks or outweighs the expected SM control.
**Terrestrial Coupling Index**	Eq. (18)	SM–E	mm/ day	−, 0, +	Strength of terrestrial control of E/LE by soil-moisture variability; larger positive values indicate stronger moisture limitation of evapotranspiration
		SM–EF	none		Strength of soil-moisture control on surface energy partitioning; larger positive values indicate stronger control of EF by SM.
		SM–H	W/m^2^		Strength of soil-moisture control on sensible heating; more negative values indicate stronger suppression of H under wetter soils.
**SM Memory**	Eq. (20)	SM	days	+	Persistence timescale of SM anomalies; longer memory means land states persist longer and can sustain predictability.
**SM Regime**	—	SM–EF	—	—	Diagnostic of hydrologic regime from the SM–EF relation, indicating water-limited, or energy-limited conditions.
**SM Breakpoints**	—	SM–EF	cm^3^/cm^3^	+	SM threshold(s) where the SM–EF slope changes, marking transitions between coupling regimes (dry, transitional, wet)

### Statistical significance testing

To test for statistical significance, we must answer two questions. Firstly, do the adjusted L-A coupling metrics differ significantly from the unadjusted metrics? Since the random error variance is obtained by fitting a parametric line to the logarithm of the ACF and extrapolating it to lag 0, the underlying assumptions are parametric; consequently, a non-parametric significance test is inappropriate. Rather than asking whether the two indices are “statistically significant” in the classical sense, we instead examine whether the magnitude of the relative random error is large enough to alter the adjusted metric significantly. To achieve this goal, we calculated the empirical cumulative probability function (CDF) of the relative random error (δ/σ), defined as the ratio of the standard deviation of random error to the standard deviation of SM measurements, shown in the Supplementary material (Supplementary Text 1, Supplementary Fig. 3).

Secondly, to determine whether the adjusted correlation significantly differs from the *in-situ* correlation from flux tower data, we apply an appropriate significance test. The standard correlation test is commonly used for this purpose; however, it assumes that the samples are independent and identically distributed (IID). Since we use daily data, autocorrelation in measured fields persists over several days for both SM and HFs, even after removing the seasonal cycle. Therefore, we apply the Fisher Z transformation, which converts Pearson’s correlation coefficient into a normally distributed variable. This transformation is useful because the sampling distribution of Pearson’s correlation coefficient is not normally distributed, especially with small sample sizes, which can complicate hypothesis testing and the construction of confidence intervals. The Fisher Z transformation formula, introduced by Fisher^120^, is as follows:

24 $$Z=\frac{1}{2}{\log }\left(\frac{1+\hat{\rho }}{1-\hat{\rho }}\right)$$

That has an approximately normal distribution with mean:

25 $${Z}_{o}=\frac{1}{2}{\log }\left(\frac{1+{\rho }_{o}}{1-{\rho }_{o}}\right)$$

And variance:

26 $${\sigma }_{Z}^{2}=\frac{1}{N-3}$$

Where Z is the Fisher Z value, $\hat{\rho }$ and ${\rho }_{o}$ represent sample and population correlations, respectively. *N* denotes the length of the time series. Therefore, the decision rule for the null hypothesis (H_o_) is that ${R}_{Adjusted}-{R}_{in-situ}=0$. Thus:

27 $$\mathrm{Under}{H}_{o}:{Z}_{Adjusted}-{Z}_{in-situ}\sim N\left(0,\frac{2}{N-3}\right)$$

If we standardize Eq. (27) by subtracting the mean and dividing by the standard deviation, we obtain:

28 $$({Z}_{Adjusted}-{Z}_{in-situ})\ast \left(\frac{\sqrt{N-3}}{\sqrt{2}}\right)\sim N(0,1)$$

and by assuming $\alpha =1 \%$ and ${Z}_{\frac{\alpha }{2}}=2.576$, the decision rule becomes:

29 $$Accept\,{H}_{o}\,if|({Z}_{Adjusted}-{Z}_{in-situ})\ast \left(\frac{\sqrt{N-3}}{\sqrt{2}}\right)| < 2.576$$

30 $$Reject\,{H}_{o}\,if|({Z}_{Adjusted}-{Z}_{in-situ})\ast \left(\frac{\sqrt{N-3}}{\sqrt{2}}\right)| > 2.576$$

Because the variance formula for Fisher’s Z presumes IID samples, in our case, N should be corrected to $N/{N}_{2}$. ${N}_{2}$ can be calculated as a cross-lag correlation, which was introduced by Davis^121^:

31 $${N}_{2}=\mathop{\sum }\limits_{\tau =-\infty }^{\infty }{{Cor}}^{2}\left({X}_{t},{Y}_{t+\tau }\right)$$

## Data Records

The Global Observationally-Based Land-Atmosphere Coupling Metrics (GOLAM)^122^ dataset has been deposited in Zenodo and is publicly available. In this section, we first describe the dataset contents, including variable names and metadata, and then explain the dataset’s file organization and structure.

### Data archive

Datasets are generally classified into three archives: coupling, SMM, and regime. The archive “coupling” includes the random error-adjusted Pearson correlation (R) between soil moisture (SM) and surface fluxes (HF), such as evaporation (E), sensible heat flux (H), and evaporation fraction (EF). The “coupling” archive also provides the two-sided p-value for the adjusted correlation, the sample size (N), the random error-adjusted terrestrial coupling index (I) between SM and HF, and the standard deviation of HF. The “SMM” archive contains random error-adjusted soil moisture memory, the variance of the random error, the variance of SM, and the relative measurement error $\delta /\sigma$, which is the random error in SM observational measurements relative to the total SM signal. The third archive is “regime,” which contains SM-EF regime identification. This archive includes 31 data variables:

1. N: Number of valid soil moisture data points after cleaning.
2. fraction_max_SM: Percentage of data points at maximum SM value.
3. BIC and RMSE for each segmented-regression model are shown as BIC_model_code and RMSE_model_code: BIC_001 or BIC_100, BIC_010, BIC_110, BIC_011, and BIC_111. Model codes are: 100: dry only, 010: transitional only, 001: wet only, 110: dry+transitional, 011: transitional+wet, and 111: dry+transitional+wet.
4. Best model code selected by BIC, Best_Model_BIC. Model codes in the datasets are 1: Model_001, 2: Model_011, 3: Model_111, 4: Model_010, 5: Model_110, 6: Model_100.
5. Breakpoints including wilting point (wp) and critical soil moisture (csm): wp_010, wp_110, wp_011, wp_111, csm_011, csm_111.
6. Slope (sensitivity): alpha_010, alpha_110, alpha_011, alpha_111.
7. The maximum constant EF for models 001, 100, and 111 is shown as EF_max__001, EF_max__100, and EF_max__111.
8. wp_selected and csm_selected: Selected wilting point/ critical SM using breakpoint selection criteria described in the soil moisture regime distribution section.
9. wp_selection_flag and csm_selection_flag, which indicate the selection step shown in Supplementary Fig. 2.

Each archive has distinct data combinations based on input observational data and their temporal coverage. The source of these input observational datasets, with their spatial resolution and time span, is available in Table 1. For consistency, combinations are provided at spatial resolutions of 0.1° and 0.25°. SMAP-GLEAM and SMAP-only combinations are available at both 0.1° × 0.1° and 0.25° × 0.25° resolutions, while other combinations are only available at 0.25° × 0.25°. “Coupling” and “SMM” archives are provided for four different seasons: June-August (JJA), September-November (SON), December-February (DJF), and March-May (MAM). However, all three archives are available for warm seasons in each region: May through September (MJJAS) for 23°N-60°N, November through March (NDJFM) for 23°S-60°S, and all months for 23°S-23°N. All combinations of each data archive with their observational input data source, period, and spatial resolution are listed in Table 2.

### Data structure

All data files are provided in the netCDF4 format, containing 2D arrays with longitude and latitude dimensions (there is no time dimension in these data files). Both archives “coupling” and “SMM” are stored in the following directory structure: <seasons/warm seasons_latitudes>: JJA, SON, DJF, MAM, MJJAS_North_Latitudes, NDJFM_South_Latitudes, All_Months_Tropics. “Regime” archive is stored in a directory called “Regime”. Data files follow this naming pattern: <METRICS>_<VARIABLES>_<PRODUCTCOMBINATIONS>_<SPATIALRESOLUTION>.nc, where <METRICS> includes: “coupling,” “SMM,” and “Regime.” <VARIABLES> correspond to “SM_E,” “SM_H,” and “SM_EF,” <PRODUCT COMBINATIONS> and <SPATIAL RESOLUTION> are listed in Table 2. For example, a file containing the adjusted correlation between SM and E, derived from CCI and GLEAM, would be named: Coupling_SM_E_CCI_GLEAM_0.25.nc.

## Technical Validation

### Consistency among coupling metrics

**Fig. 3:**
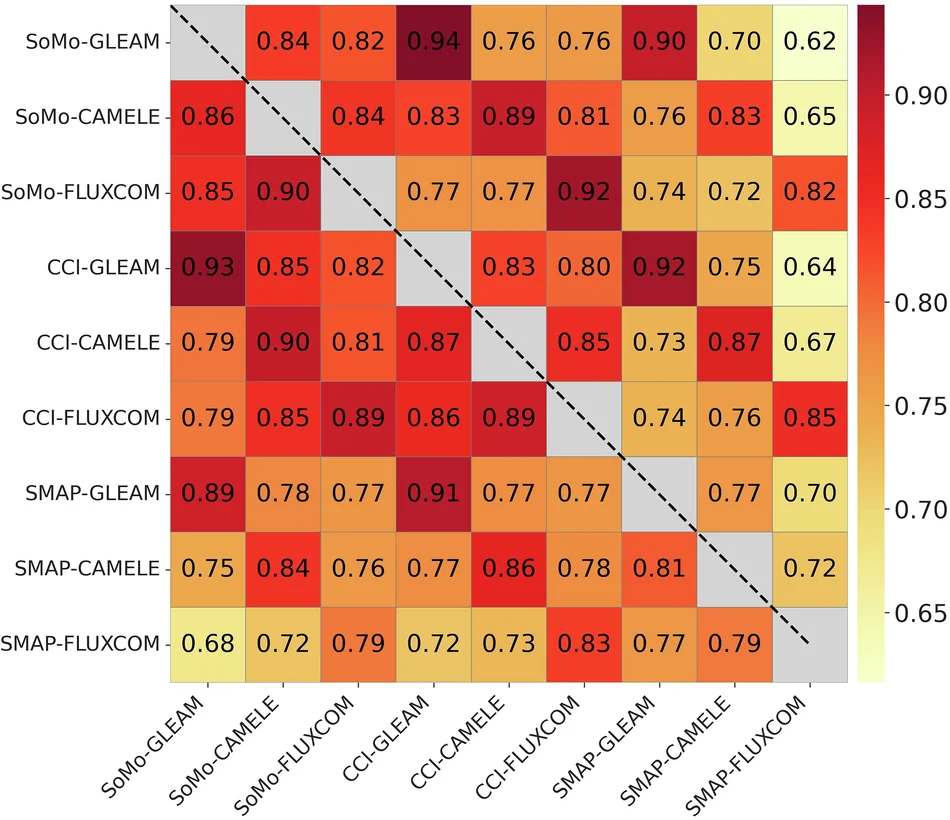
Pairwise Spearman rank correlations among (upper triangle) the adjusted terrestrial coupling index ${I}_{Adjusted}(SM,E)$ combinations and (lower triangle) the adjusted correlation ${R}_{Adjusted}(SM,E)$. Colors depict the Spearman rank correlation scale shown on the right; the dashed diagonal separates the two sets.

To quantify the degree of agreement or disagreement among the adjusted correlation and terrestrial coupling index combinations, or in other words, how similarly each pair of maps ranks land pixels, we calculated the spatial correlation using Spearman’s rank correlation between each pair of combinations. Figure 3 shows the Spearman rank-correlation coefficients for every pair of the nine ${I}_{Adjusted}(SM,E)$ maps (upper triangle) and the nine ${R}_{Adjusted}(SM,E)$ maps (lower triangle).

Spatial pattern similarity quantified using Spearman rank correlations and hierarchical clustering dendrograms based on 1-minus Spearman rank correlations ($D=1-{\rm{Spearman}}\,R)$ among the nine ${R}_{{Adjusted}}({SM},E)$ maps (Fig. 3, lower triangle; Supplementary Fig. 4a) further quantifies inter-product correspondence. The lower triangle in Fig. 3 shows that nearly all Spearman-rank correlations among ${R}_{{Adjusted}}({SM},{E})$ combinations exceed 0.70, and many are above 0.80; therefore, each product combination generally displays the same overall correlation pattern. The strongest agreement occurs between SoMo-GLEAM and CCI-GLEAM (Spearman r =0.93, D=0.07), SMAP-GLEAM then joins this sub-cluster (D ≈ 0.10; Spearman r ≳ 0.89 relative to each). A parallel tight branch is formed by two sub-clusters: (CCI-FLUXCOM, SoMo-FLUXCOM) and (CCI-CAMELE, SoMo-CAMELE), each with D ≈ 0.10 and Spearman r ≳ 0.89. The near-identical pixel rankings within this four-combination branch provide multi-product corroboration of the underlying ${R}_{{Adjusted}}({SM},E)$ spatial pattern. They could serve as a high-confidence subset for model benchmarking. Especially within SM product consistency, SoMo-based pairs show internal Spearman correlations ranging from 0.85 to 0.90, and CCI-based pairs demonstrate a similar range (Spearman r = 0.86–0.89). This coherence indicates that each SM product maintains its characteristic correlation signature regardless of the E product used, highlighting the robustness of the underlying SM–E relationships.

In contrast, SMAP-based combinations exhibit lower Spearman correlations with both SoMo- and CCI-based maps, with the weakest relationship observed between SMAP-FLUXCOM and SoMo-GLEAM (Spearman r = 0.68). Overall, all SMAP combinations sit outside this first branch, especially for SMAP-CAMELE and SMAP-FLUXCOM, which remain most distant (D ≈ 0.20–0.25). Despite this, the adjusted correlation between SM and E, ${R}_{Adjusted}(SM,E)$, is highly robust across all data choices since the distance ($D=1-{\rm{Spearman}}\,R$) ranges only from 0 to 0.25. This means that 75% of the pixel ranking is still shared among maps, even in the worst case.

Consistent with the correlation analysis, the highest spatial Spearman correlations among the nine ${I}_{{Adjusted}}({SM},E)$ combinations occur between SoMo-GLEAM and CCI-GLEAM (Spearman r = 0.94; Fig. 3; upper triangle), followed by SoMo-FLUXCOM and CCI-FLUXCOM (Spearman r = 0.92), with SMAP-GLEAM and CCI-GLEAM (Spearman r = 0.92). Overall, within-product consistency further reflects this E-dominant behavior. Pairs that share the same E products display higher internal correlations: GLEAM-based combinations range from 0.90 to 0.94, while FLUXCOM-based combinations range from 0.82 to 0.92. In contrast, combinations that mix E methodologies exhibit significantly lower correlations, with the weakest relationship observed between SoMo-GLEAM and SMAP-FLUXCOM (Spearman r =0.62). As a result, the impact of E on the spatial patterns of the coupling index is considerable.

The spatial Spearman correlations of the SM–E coupling index (Supplementary Fig. 4b) reveal a distinctly different grouping pattern and sensitivities compared to the correlation ${R}_{{Adjusted}}({SM},E)$ analysis. The dendrogram (Supplementary Fig. 4b) shows that all three CAMELE combinations first merge with the three GLEAM combinations, and that combined branch subsequently joins the FLUXCOM branch. In other words, CAMELE looks more like GLEAM than FLUXCOM, consistent with the weighting of low-error members in the CAMELE blend. Because GLEAM generally carries lower random error in many regions, CAMELE inherits some of GLEAM’s spatial structure, especially across water-limited regions, since GLEAM estimates ET using an explicit dependence of SM.

While the correlation analysis suggests that the SM product is the main source of variation, the choice of E product is the primary driver of the variability in the coupling index. This structural shift suggests that the coupling index is more sensitive to the flux variability term embedded in its definition (correlation × σ_E_). In practical terms, ${I}_{{Adjusted}}$ reflects the efficiency with which SM anomalies translate into turbulent flux anomalies under the local energy balance and atmospheric demand. Thus, differences in how E products partition available energy, represent canopy processes, and encode stress scalars emerge as the leading source of variation.

Similar to the spatial Spearman rank correlations between ${R}_{Adjusted}(SM,E)$ combinations, SoMo-GLEAM and CCI-GLEAM pairs show the strongest overall agreements for both adjusted correlation and coupling index between SM and EF anomalies (Supplementary Figs. 5a, 6) and between SM and sensible heat flux anomalies (Supplementary Figs. 5b, 7). While the lowest agreement is observed between the SoMo-GLEAM and SMAP-GLEAM pairs for ${R}_{{Adjusted}}({SM},H)$, with a Spearman’s r of 0.77. Details of pairwise Spearman rank correlations among ${I}_{{Adjusted}}$ and ${R}_{{Adjusted}}$ for SM-EF and SM-H, and the grouping pattern across all metrics are explained in the Supplement (Supplementary Text 2, 3, Supplementary Figs. 4–7).

Generally, these results highlight the dominant influence of the SM product choice over surface flux product choice in determining spatial Spearman’s rank correlation patterns between adjusted correlation combinations. This suggests that observational uncertainty in SM is a chief limitation for global land-atmosphere coupling studies. The pronounced similarity between SoMo and CCI products, despite their different retrieval methodologies, suggests that both capture comparable underlying SM anomalies relevant for ET variability across scales. Each is a multi-sensor microwave product, bias-harmonized, and then smoothed via machine-learning approaches that reduce random retrieval noise. Conversely, SMAP relies primarily on a single-sensor L-band radiometer with deeper penetration; thus, its retrieval physics responds to soil texture and vegetation optical depth differently, leading SMAP to remain an outlier from the sub-cluster formed by SoMo and CCI. The shorter length of data record for SMAP is likely also a factor, as discussed before.

### Geographical factors

**Fig. 4:**
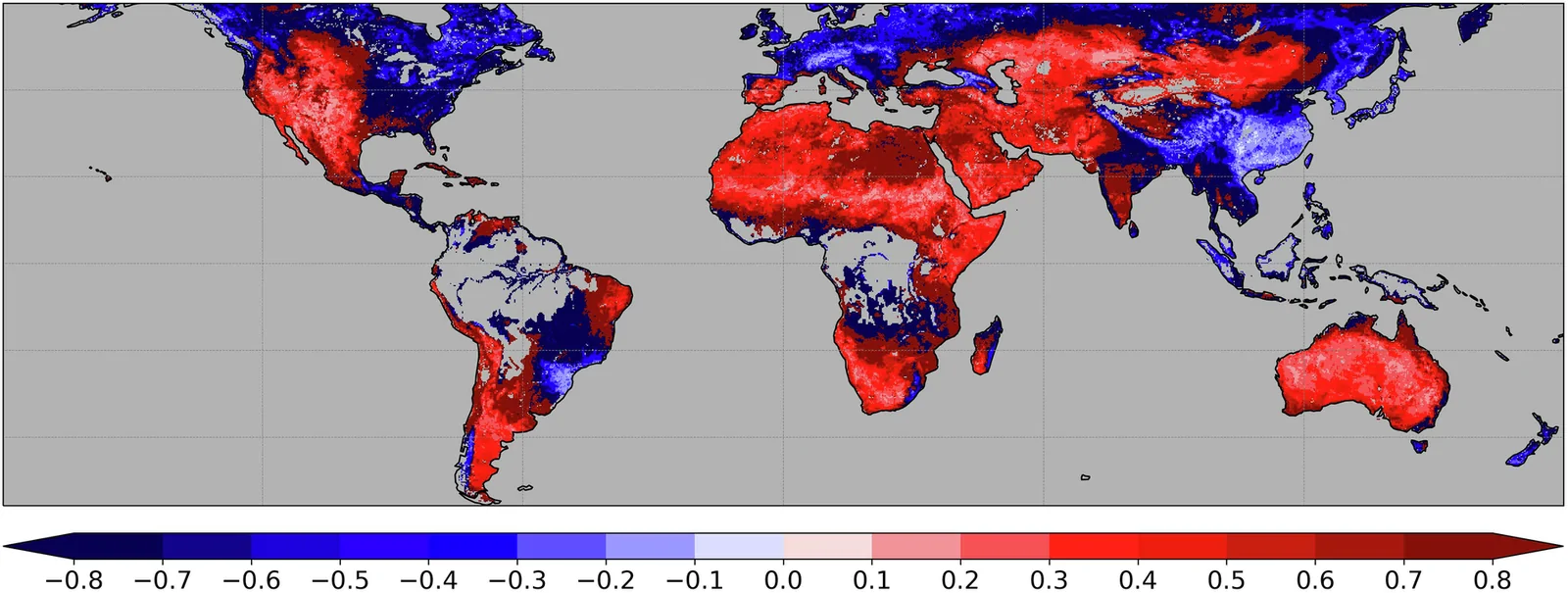
Global distribution of the coefficient of variation (CV) in Fisher z-space among nine ${R}_{{Adjusted}}({SM},E)$ pairs. Gray areas indicate incomplete data.

To identify where the nine ${R}_{{Adjusted}}({SM},{E})$ combinations agree or disagree, we applied the Fisher z-transform (*z* = tanh^−1^ *R*) to stabilize variance, then calculated the ensemble mean (μ) and standard deviation (σ). The spatial pattern of inter-product dispersion was summarized with the coefficient of variation (${\rm{CV}}=\sigma /\mu$) in z-space. The results are shown in Fig. 4. In this figure, the darker shades—red for positive values and blue for negative—represent regions with lower agreement (higher dispersion). Conversely, lighter shades indicate areas of higher agreement (lower dispersion) across all correlation combinations. Gray areas indicate grid cells for which at least one combination of data is missing.

Figure 4 reveals strong inter-product consensus in several regions. Light red areas, which indicate that various combinations of ${R}_{{Adjusted}}({SM},{E})$ consistently agree on both the strength and positive sign of correlation, appear throughout the central and western U.S, interior Australia, parts of South America (e.g., the Pampas, the Brazilian Cerrado, and Patagonia), southern Africa, parts of the Sahel, the Horn of Africa, parts of the Mediterranean (Spain, southern France, and Italy), Central Asia, and large parts of Eurasia. Conversely, light blue areas, which suggest strong agreement in negative dispersion among combinations, are mostly found in temperate Europe, eastern China, and parts of temperate South America.

However, the spatial uncertainty in inter-product comparisons of ${R}_{{Adjusted}}({SM},E)$ can be amplified by factors such as hydroclimatic control (chronic aridity or persistent humidity) and mixed land use. Based on Fig. 4, the extensive dark blue areas, which reveal pronounced disagreement in negative correlations, are found across the eastern U.S., southeastern Canada, Central America, northern Argentina, eastern Bolivia, Siberia, South and East Asia (e.g., northern India, southern China, Thailand), Equatorial Africa, and parts of Eastern Europe.

Thailand exhibits low agreement, perhaps due to mixed land uses (upland forests vs. flooded rice paddies), complex topography, and persistent cloud cover that complicates both SM retrievals and E estimates. In the eastern United States, synoptic variability, seasonal swings in radiation and humidity, and heterogeneous land cover modulate coupling in ways that appear to make ${R}_{{Adjusted}}({SM},E)$ differ between combinations. Additionally, when ${R}_{{Adjusted}}({SM},E)$ values stay near zero, insignificant correlations, the coefficient of variation increases because the ensemble mean is very small. This occurs in regions such as Egypt, Southeast Asia (e.g., northern India and Thailand), and the Eastern US. Insignificant correlations at the 5% significance level (two-sided Student’s t-test; p≥0.05) are shown as white areas in (Supplementary Figs. 8–10).

Conversely, the dark red areas, which indicate high variability or divergence in the positive correlations, occur mainly near the boundaries between water- and energy-limited regimes. These areas are mainly located in the central U.S., Eurasia, the Sahel, and northern and southwestern Australia, as well as in southern India, Tibet, parts of the Middle East, eastern Brazil, and northern Argentina. For instance, the pronounced monsoonal wet season followed by a long, hot dry season in southern India can drive each ${R}_{{Adjusted}}({SM},E)$ pair to diverge. Generally, dark red areas are largely in the transition zones between positive and negative correlations.

With this in mind, comparison of the coefficient of variation (Fig. 4) reinforces that ${R}_{{Adjusted}}({SM},E)$ is robust over most land areas (light-shaded colors); the sign and approximate magnitude of ${R}_{{Adjusted}}({SM},E)$ remain consistent among different combinations and across much of the globe. These regions, therefore, mark where ${R}_{{Adjusted}}({SM},E)$ fields are most trustworthy for model validation. Even some red areas, which indicate high variability or divergence in the positive correlations, are informative for model evaluation since they are mainly located in the vicinity of boundaries between water- and energy-limited regimes. As a result, the spatial pattern of inter-product dispersion in ${R}_{{Adjusted}}({SM},E)$ highlights not only where uncertainty is greatest, but also where the expected hydroclimatic partition of land-atmosphere coupling regimes lies.

Supplementary Figs. 11-12 takes this analysis further, by illustrating the spatial distribution of CV in Fisher z-space for ${R}_{{Adjusted}}({SM},{H})$ and ${R}_{{Adjusted}}({SM},{EF})$ across three combinations using GLEAM (Details in Supplementary Text 4).

**Fig. 5:**
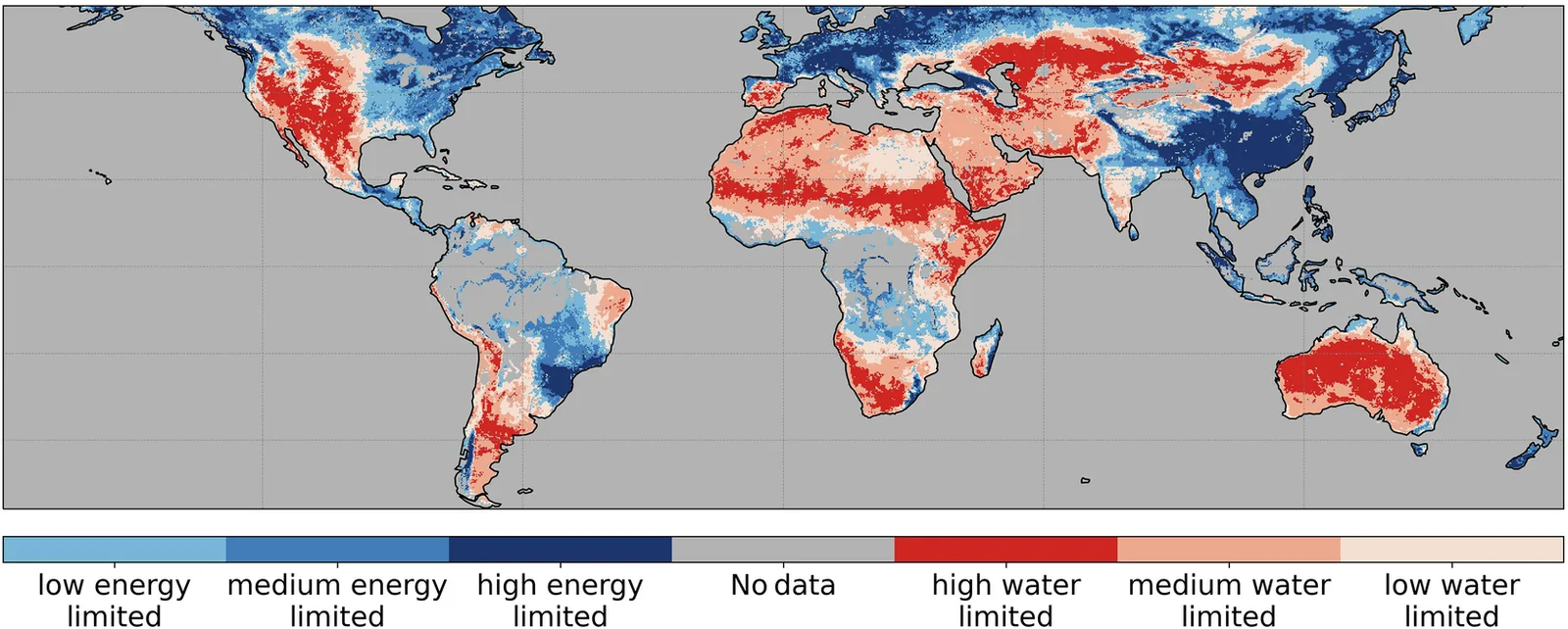
Global classification of energy- versus water-limited regimes, along with regions of low, medium, or high inter-product agreement, based on the coefficient of variation (CV) in Fisher-z space across nine ${R}_{{Adjusted}}({SM},E)$ combinations. Gray areas indicate incomplete data.

Figure 5 presents a global classification of land areas into energy- and water-limited coupling regimes, based on CV in Fisher-z space, as described in Supplementary Text 5 and illustrated in Supplementary Fig. 13, across nine ${R}_{{Adjusted}}({SM},{E})$ pairs. The colors indicate both the dominant regime type (energy-limited in blue, water-limited in red shades) and the degree of inter-product agreement (from high to low, darker to lighter shades). Gray areas denote regions where at least one of the nine combinations has missing data.

Energy-limited regimes, represented in blue in Fig. 5, dominate high-latitude and humid temperate regions. Most of northern Europe, eastern Canada, the northeastern U.S., Siberia, and parts of East Asia show energy-limited behavior with high or medium agreement among ${R}_{{Adjusted}}({SM},{E})$ combinations. However, energy-limited regimes are also found in parts of tropical South America, central Africa, maritime Southeast Asia, and northern India, with lower agreement indicated by lighter shades. Additionally, there is a light blue stripe in northern Europe from France to Poland, which is nested within an otherwise low-agreement energy-limited regime.

Water-limited regimes (shades of red) are widespread across semi-arid and subtropical regions, such as North Africa, the Sahel, the Middle East, southern Africa, eastern Brazil, central and western U.S., and most of Australia. However, areas in light red, indicating lower agreement, are mostly found in the transitional zones, including the central U.S. plains, Eurasia, southern India, Tibet, northern Australia, and parts of the Sahel.

The locations of boundaries between regimes are well defined as low agreement water- and energy-limited in Fig. 5. Although these boundaries between water- and energy-limited regimes in places like the U.S., the edges of Congo in Africa, Eurasia, and South America, may reflect sensitivity to seasonal shifts, vegetation heterogeneity, or methodological differences across datasets, all ${R}_{{Adjusted}}({SM},E)$ combinations agree within a narrow band of a few hundred kilometers. The boundaries between water- and energy-limited regimes correspond to ${\theta }_{{crit}}$, which is not a stationary point in SM as it fluctuates with atmospheric and land conditions^123^. A band of a few hundred kilometers may be as narrow as could be defined. Within such a margin of error, the accurate placement of these boundaries is critical for weather and climate model performance and development, because if they are not well positioned, a model’s land-atmosphere feedbacks and hydroclimatology will be inaccurate^7^. Alongside correct partitioning of hydroclimate boundaries in models, the degree of consensus in SM regimes among observational SM-EF combinations can act as a confidence score to validate model SM regimes.

### Consistency of SM memory

To compare the spatial patterns of the disparate adjusted SMM fields, we analyze the percentiles within their global distributions. CDF-matching is used to map the cumulative distribution values from one SMM field to that of another, which enables us to compare different SMM fields without over- or under-emphasizing differences at either the low or high ends of the spectrum, while also attempting to ameliorate the apparent biases between SoMo and the other products. In other words, absolute changes are essential at the shorter time scales, while relative changes are more informative in the long tail.

**Fig. 6:**
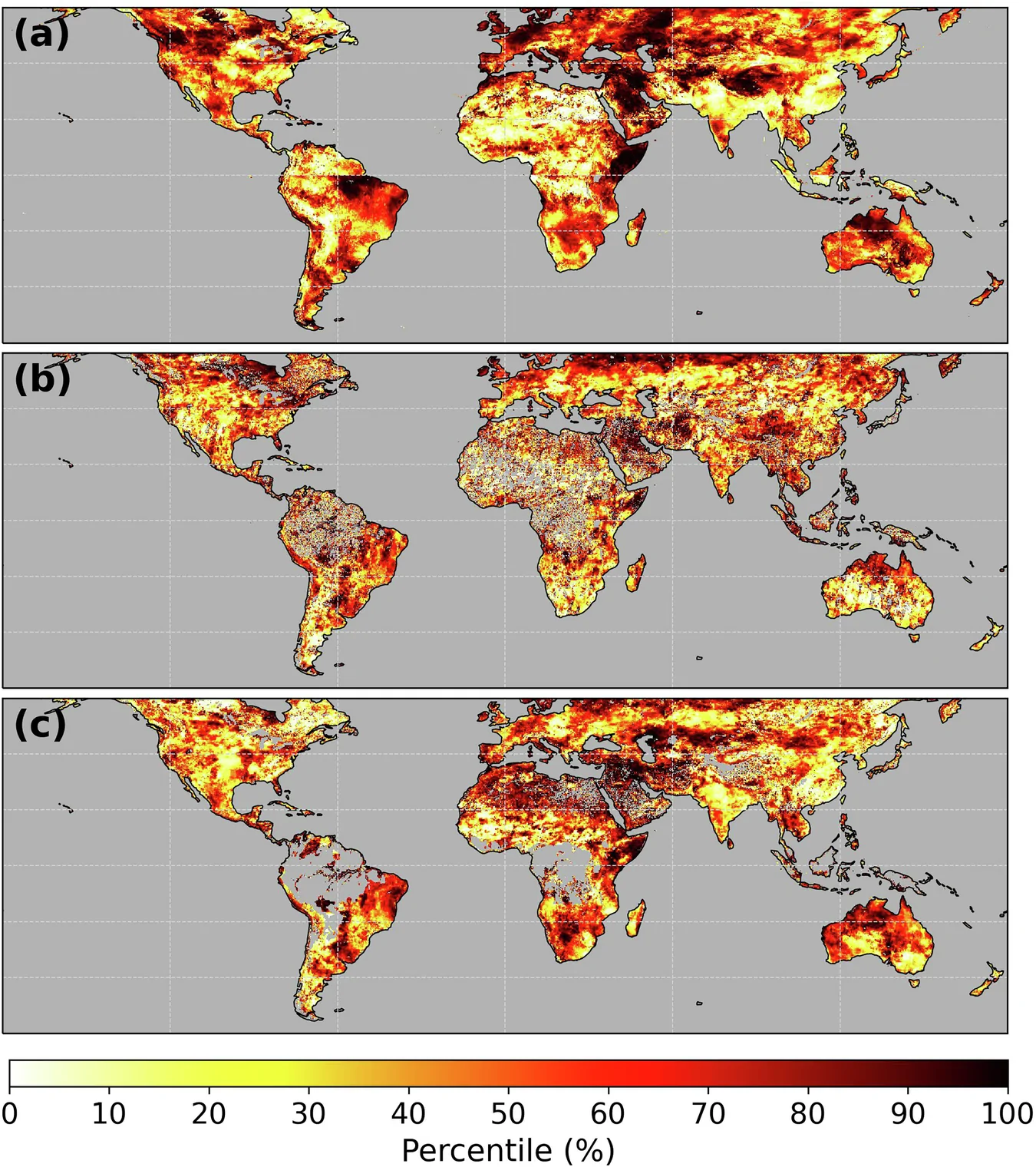
Global percentile maps of adjusted soil moisture memory for (**a**) SoMo-L1, (**b**) SMAP, and (**c**) CCI at 0.25° for periods of 2015-2019. Gray areas indicate missing values.

All three SM datasets exhibit quite different SMM when calculated over their entire periods of record: SMAP (March 2015-April 2025), CCI (1979-2023), and SoMo (2000-2019). These results are not shown here but can be found in the final datasets. However, when SMM is calculated over only their 4-year overlapping period, they are in better agreement, but there is still a fair amount of disparity. Thus, we convert each SMM field to its global percentile and produce global maps in percentile space (Fig. 6) to show where each grid point falls within the product’s own probability distribution.

Although all three SM datasets show similar spatial patterns of short- and long-memory in some regions (Fig. 6), there are notable regional differences in percentile magnitudes of SMM, even though the same 4-year period (April 2015–May 2019) is used for all. Figure 6a shows the global percentile maps of adjusted SMM for SoMo-L1 over 2015-2019 at a spatial resolution of 0.25°. SoMo-L1 displays the prominent high SMM regions over the globe, with large areas in the upper tail of the SoMo-L1 distribution. This may be because SoMo-L1 is representative of water in slightly deeper soil (0-10) cm, which can lead to lower day-to-day variance (longer SMM) and a smoother time series in which random errors and high-frequency noise are damped. In addition, SoMo is trained with meteorological inputs, such as precipitation, which can implicitly account for subsurface moisture recharge and losses.

Another notable difference appears at high latitudes, where SoMo shows extended SMM across the Eurasian steppe, while SMAP and CCI (Fig. 6b,c), detect long memory only in the far north. Limited precipitation and low evaporative demand in cold climates lead to higher persistence in SM, resulting in longer SMM at high latitudes^114^. This highlights the impact of seasonal cycles on SMM, showing that long dry or cold seasons extend anomalies^124,125^. Thus, temperature seasonality dominates SMM at higher latitudes, while hydrological variability has more influence at lower latitudes.

In addition to high-latitude Eurasia, the highest SMM percentile in SoMo-L1 is found across Canada, the Horn of Africa, the Arabian Peninsula, Tibet, parts of northern and interior Australia, northern Brazil, and the southeastern Amazon Basin (Fig. 6a). Conversely, locations such as the western US, China, northern India, North America, and the northern Amazon Basin show values in the lower SoMo-L1 percentile range (<45%).

Compared to SoMo-L1, SMM patterns derived from SMAP (Fig. 6b) show a significant decrease in both the extent and intensity of SMM percentiles. Peak memory percentiles are mostly found across North America, northern Eurasia, Tibet, northern Australia, and some areas in the Middle East and northern Brazil. Generally, regional contrasts become more noticeable in seasonally wet–dry climates, especially in monsoon and agricultural areas. In parts of China and India, SMAP shows higher SMM percentiles compared to SoMo and CCI (Fig. 6a,c). A likely reason is that SMAP’s direct L-band measurements can detect sustained SM from irrigation during the dry season or after rain. Recent work confirms that SMAP Level 3 captures irrigation impacts, while model-assimilated products may not^126^. Secondly, soil properties such as porosity and soil field capacity^39,51^ are also important factors for SMM. Higher clay contents and organic soils can result in higher SM spectra^127^ and extend memory by holding water and drying more slowly after precipitation events. SMAP’s retrievals may be more sensitive to soil hydraulic properties^128^, whereas the generalized SoMo framework cannot physically account for them.

Conversely, SMAP (Fig. 6b) consistently shows lower memory percentiles in North Africa, Patagonia, much of Australia (except northern parts), the eastern and western US, and tropical regions. In regions with frequent rainfall, like tropical regions, SM anomalies are hard to sustain due to frequent soil saturation, resulting in shorter SMM in humid and wet areas. This could explain why, across the rainforest belt, such as the Amazon and Congo Basins, SMM percentiles are consistently low in both SMAP and SoMo (Fig. 6a,b), while SMAP exhibits slightly higher SMM percentiles than SoMo. Frequent, intense rainfall and rapid drainage cause SM anomalies to have low persistence in soil; therefore, a wet anomaly is quickly reset by drainage or ongoing precipitation over these regions. Higher transpiration rates, which boost precipitation due to vegetation activities, often buffer SM variations and display weaker memory^125^.

Figure 6c also shows that the highest CCI memory percentiles are found in Eurasia, the Middle East, the Horn of Africa, as well as northern and southern Africa. In arid and semi-arid regions like the Sahara, the Arabian Peninsula, central Australia, and the southwestern US, CCI shows high SMM percentiles, while SMAP and SoMo rank in the lower percentiles over the Sahara (Fig. 6a,b). Overall, although arid regions experience longer SMM, in desert areas where SM variability is very low and precipitation is nearly absent, the signal may be overwhelmed by noise, despite our efforts to adjust for the random error component and not reveal meaningful memory. This challenge is evident in the Sahara, where CCI detects longer SMM. Additionally, the northern and interior regions of Australia display high CCI memory percentiles; however, the southeastern regions and parts of southwestern Australia show lower CCI memory percentiles. Despite overall high CCI SMM percentile values, China, India, the eastern and central US, the Sahel, North Eurasia, and Patagonia exhibit significantly lower CCI SMM percentile values.

**Fig. 7:**
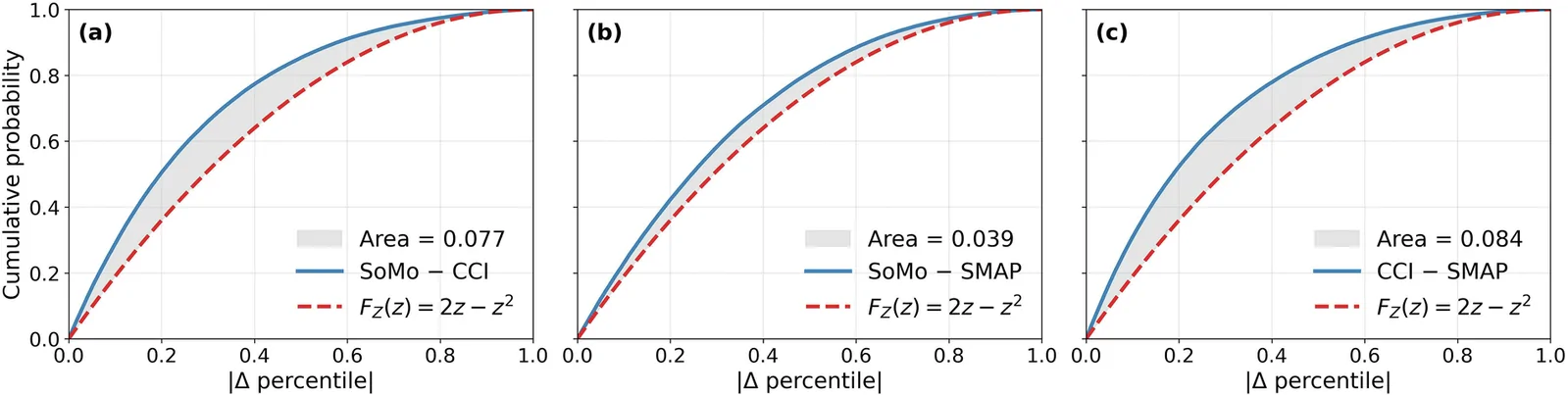
CDFs of absolute differences in SMM percentiles for three SM product pairs (blue): (**a**) SoMo−CCI, (**b**) SoMo−SMAP, and (**c**) CCI−SMAP. The red dashed line corresponds to the CDF of absolute differences in SMM for two random time series. The shaded grey areas indicate the divergence between the observed and random distributions, and the area of each shaded region is reported within its corresponding panel.

To identify areas of agreement and disagreement between each pair of adjusted SMM fields, we create three pairwise percentile-difference maps. Details of the pairwise percentile comparison are provided in the Supplementary Materials (Supplementary Text 6, Supplementary Fig. 14). Subsequently, to evaluate the statistical significance of the adjusted SMM agreements or disagreements in pairwise percentile comparisons, we use a shuffled surrogates method with a null hypothesis that there is no systematic difference between the observed comparison and a random comparison. First, we randomly shuffle the CCI time series at each grid cell in time to create a new sequence of time series. We repeat this process to generate additional independent, randomly shuffled time series, and we then compute the adjusted SMM from each shuffled time series at every grid cell. Next, we calculate the pairwise percentile-difference map between these two random SMM fields. Finally, we derive the CDF of absolute percentile differences between the two random fields and, separately, the CDF of absolute percentile differences between the adjusted SMM values from the three observational datasets (CCI, SoMo-L1, and SMAP). For two independent random time series (white noise), the CDFs of SMM follow a normal distribution $\left(\mu =0,\,\sigma =1\right)$, and the absolute difference between their CDFs have a theoretical equation ${F}_{Z}(z)=2z-{z}^{2}$ (see Supplementary Text 7 for the derivation of this equation). If we consider |Δ-percentile|=0 as perfect agreement, the ratio of the area between the random and observed |Δ-percentile| curves to the area between the random and perfect-model curves indicates how far we are from a perfect match. The area between the random and perfect model is a constant value of 0.33 (see Supplementary Text 8 for details on how this value is derived). Figure 7 shows the absolute percentile differences between the observed SMM products (blue lines), while the red dashed lines indicate the |Δ-percentile| of SMM derived from the random time series. The shaded grey areas illustrate the deviation between the observed and null distributions.

Figure 7 shows that the areas indicating the deviation between the observed and null distributions are 0.077 for SoMo – CCI, 0.039 for SoMo – SMAP, and 0.084 for CCI – SMAP. In other words, the observed curves are 24%, 12%, and 26% of the way from pure randomness toward perfect agreement for SoMo–CCI, SoMo–SMAP, and CCI–SMAP, respectively. Consequently, the statistical significance of the adjusted SMM indicates that, among SMMs derived from CCI and SMAP, which show the highest similarity, only 26% of perfect matches are recovered (Fig. 7c), while the SoMo–SMAP pair has the least similarity. Various factors, including atmospheric forcing, anthropogenic activities, soil hydrological forcing, soil properties, groundwater dynamics, vegetation properties, soil-atmosphere coupling, root structure, topography, and subsurface geology^129^, can all affect SMM dynamics. Any discrepancies among SM datasets in how they handle or measure these factors can impact uncertainties in the estimation of SMM.

Generally, the results of adjusted SMM suggest that relying on only one SM product to estimate SMM, even if that product has high accuracy in measuring SM, may not be reliable. Although all three SM datasets broadly reflect the expected climate-driven SMM pattern, there is less agreement among datasets on this metric compared to what our results have shown for adjusted correlations, coupling index, and SM regime.

### Consistency of SM regimes

To assess spatial discrepancies in detected SM regimes, we performed a grid cell level comparison of the best-fitting six-segment regression models selected by each SM-EF product pair. Following Hsu and Dirmeyer^45^, we categorized these six candidate models into three distinct groups based on their binary structures. For example, models that include a “dry” regime, which is indicated by the first binary digit (e.g., 111, 110, 100), were grouped as “dry-inclusive.” For each SM-EF product pair, we then calculated the absolute difference in the “dry” binary digit, defined as ${\triangle }_{{dry}}$. A ${\triangle }_{{dry}}$ value of 1 signifies disagreement in identifying the dry-inclusive regime at a specific grid point, while a value of 0 indicates agreement. Similarly, candidate models with a transitional regime are indicated by the second binary digit (e.g., 011, 111, 010, 110), and a wet regime by the third binary digit (e.g., 001, 011, 111). The absolute differences in their corresponding binary masks were computed and labeled ${\triangle }_{{trans}}$ and ${\triangle }_{{wet}}$, respectively. Finally, summing these three absolute differences yields an overall disagreement index, Σ∆, which ranges from 0 (complete agreement across all regime classifications) to 3 (complete disagreement).

**Fig. 8:**
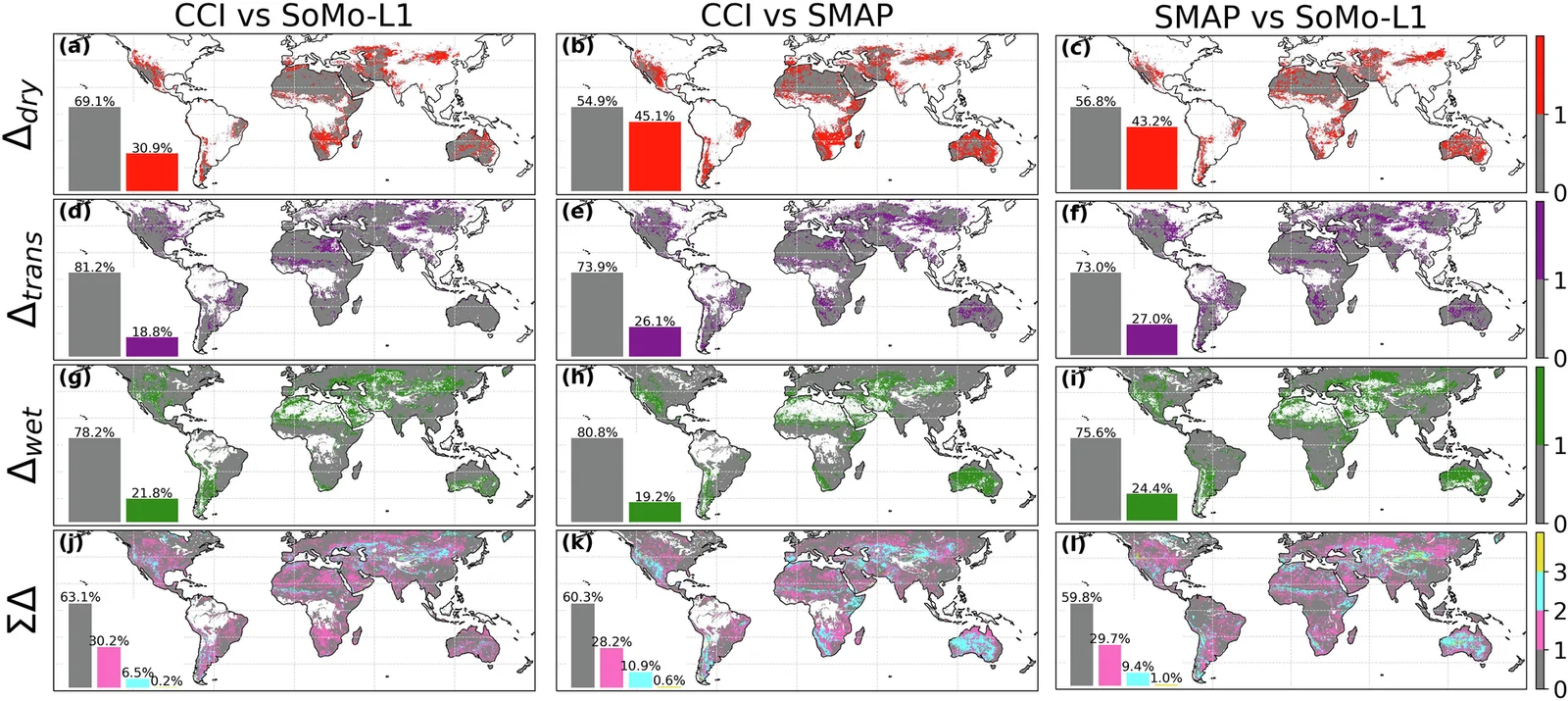
Spatial consistency of SM regime classifications between product pairs shown by columns (left to right): CCI vs. SoMo-L1, CCI vs. SMAP, and SMAP vs. SoMo-L1. Rows show absolute differences for dry-inclusive digit (${\triangle }_{{dry}}$), transitional-inclusive digit (${\triangle }_{{trans}}$), wet-inclusive digit $({\triangle }_{{wet}}$), and the total sum of disagreement index (Σ∆). Grey cells indicate complete agreement between products (∆ = 0). Colored pixels denote disagreement: red for ${\triangle }_{{dry}}$, purple for ${\triangle }_{{trans}}$, green for ${\triangle }_{{wet}}$, and grey, pink, cyan, or gold for Σ∆ values of 0, 1, 2, or 3, respectively. The inserted bar charts display the fraction of land area (in %) occupied by each category for the corresponding map.

Figure 8 presents spatial comparisons for the dry-inclusive (${\triangle }_{{dry}}$), transitional-inclusive (${\triangle }_{{trans}}$), and wet-inclusive (${\triangle }_{{wet}}$) regimes in the first three rows, respectively, while the bottom row displays the total summed disagreement index (Σ∆). Columns (from left to right) correspond to the product pairs CCI vs. SoMo-L1, CCI vs. SMAP, and SMAP vs. SoMo-L1. GLEAM is omitted from the labels because it is the shared EF dataset. Gray pixels denote full agreement between the two products, while colored pixels highlight grid cells where their regime assignments differ. Inset bar charts quantify the fraction of land area falling into each category. Grid cells lacking valid data in both products are masked in white.

The pairwise comparison shows that CCI vs. SoMo-L1 exhibits the strongest agreement in the locations of ${\triangle }_{{dry}}$ (Fig. 8a–c), with approximately 69% of all dry pixels. CCI vs. SMAP and SMAP vs. SoMo-L1 show lower agreement rates with about 55% and 57% of dry pixels, respectively. Figure 8a indicates that CCI vs. SoMo-L1 has a high level of agreement in detecting of ${\triangle }_{{dry}}$ across arid and semi-arid regions, including the Sahara and Arabian deserts, the Atacama, interior Australia, the Horn of Africa, southern African drylands, the Iranian Plateau deserts, parts of Central Asia, the southwestern U.S., and northern Brazil. In contrast, disagreement in detection of ${\triangle }_{{dry}}$ between CCI and SoMo-L1 is concentrated over the Great Plains, southern Africa, northern Australia, and parts of the Eurasian steppe. The pattern of disagreement in ${\triangle }_{{dry}}$ for CCI vs. SMAP is similar to that for CCI vs. SoMo-L1, but the mismatch between CCI and SMAP is more extensive across southern Africa, interior and northern Australia, and the Great Plains. Meanwhile, both SMAP and SoMo-L1 appear to detect fewer dry-inclusive regions than CCI, as evidenced by the smaller grey and red pixels in the SMAP vs. SoMo-L1 panel (Fig. 8c).

Similar to dry-inclusive analysis, CCI vs. SoMo-L1 agrees most closely (81.2%) in detection of transitional-inclusive (Fig. 8d), while agreement declines for CCI vs. SMAP (73.9%) and SMAP vs. SoMo-L1 (73%) (Fig. 8e,f). Spatially, all three ${\triangle }_{{trans}}$ panels (Fig. 8d–f) display a similar pattern of agreement or disagreement. The disagreement in detection of ${\triangle }_{{trans}}$ is mostly scattered across the Eurasian steppe, parts of the Sahel, and Egypt. By contrast, the panels that include SMAP (Fig. 8e,f) exhibit broader, more continuous swaths of disagreement that extend into the Eurasian steppe, southern Africa, South America, and interior Australia.

According to Fig. 8h, the highest level of agreement in detecting the wet-inclusive regime occurs in the CCI vs. SMAP (80.8%), followed by CCI vs. SoMo-L1 (78.2%; Fig. 8g), while the lowest match agreement is between SMAP and SoMo-L1 (75.6%; Fig. 8i). Generally, all three ${\triangle }_{{wet}}$ panels (Fig. 8g–i) display a common spatial pattern of mismatch pixels where only one product detects the wet regime. These mismatches mainly appear over western and central North America, South America, small patches in southern Africa, the Eurasian forest-steppe, the Middle East, and Australia. However, CCI vs. SMAP (Fig. 8h) shows slightly higher mismatches in interior Australia, southwest Africa, and the Horn of Africa. Additionally, Fig. 8g indicates that SoMo-L1 diverges more extensively from CCI in North America, the Eurasian forest-steppe, and the Middle East. The SMAP vs. SoMo-L1 reflects these SoMo-specific differences and demonstrates additional discrepancies in northern Eurasia and parts of central to western Australia (Fig. 8i).

Generally, the strongest agreement occurs in the transitional and wet regimes (gray areas in Fig. 8d–i), covering about 73%-81.2% of the land area, indicating that the three SM regime combinations agree well on the location of the sensitive regime, which is most critical to physical processes linking L-A feedback. Within this regime, SM–EF coupling is dominant; thus, the transitional regime is where nearly all of the sensitivity of EF to SM occurs, widely recognized as “hotspots” of land–atmosphere interactions and a key for extremes and predictability.

However, agreement in the detection of dry regimes is lower among combinations (Fig. 8a–c), with around 55%-70% of land area. Although detecting the dry regime is itself crucial for diagnosing the strong sensitivity of sensible heat flux to SM^64,130^, which implicates positive feedbacks during heatwaves, the greatest uncertainty is in an otherwise insensitive part of the spectrum, minimizing the consequences of the uncertainties.

Several factors, such as SM data characteristics and SM-EF records, can contribute to the discrepancies seen in the results. For instance, the length of SM-EF records can impact the capture of many extreme events, such as drought and floods, through which both wilting and critical SM thresholds can emerge in the data. The SMAP time series spans about a decade, which is insufficient to detect long periods of extreme dry downs and wet spells. Short records yield smaller samples and narrower variability, making breakpoints harder to detect statistically. In addition to the SM-EF record length, the sampling density and missing data influence the likelihood of observing both wilting points and critical SM in a pixel, and thus the ability to select the candidate 111. Although SoMo and CCI have missing values in their time series, SMAP’s 2–3-day revisit leads to gaps that can more easily under-sample short-lived extremes and further narrow the SM range.

Despite these contrasts in SM data characteristics and record lengths, there is robust product-to-product agreement in regime detection in many regions. Across the three Σ∆ panels (Fig. 8j–l), the products agree on the full set of dry-, transitional-, and wet-inclusive regimes (Σ∆= 0) across most land areas, the grey areas in Fig. 8j–l, which cover about 60% of land areas between each pairwise combination, highlight a high level of spatial similarity in the detected regimes. Therefore, these observational SM-EF combinations provide a practical benchmark for validating regimes in models.

However, the degree of pairwise agreement varies between pairs. For example, CCI and SoMo-L1 align most closely, assigning identical regimes to 63.1% of land pixels, followed by CCI vs. SMAP and SMAP vs. SoMo-L1 (≈60% each). However, the single-digit disagreements (Σ∆ = 1) are about 30% of the land area in all three comparisons, while conflicts involving two or three digits rarely exceed one-eighth of the global land surface.

Overall, Fig. 8j–l also show single-digit disagreements (Σ∆ = 1) are mostly dominant in the Sahara, the Middle East, southern Africa, interior and northern Australia, northern Eurasia, India, Southeast Asia, and both North and South America. In the CCI vs. SoMo-L1 panel (Fig. 8j), two-digit conflicts (Σ∆ = 2) appear mainly across the Sahel, the Eurasian Forest–Steppe, and patches in Patagonia. However, the two-digit conflict expands in the CCI vs. SMAP and SMAP vs. SoMo-L1 panels (Fig. 8k–l), extending into interior Australia, the Sahel, Patagonia, the Horn of Africa, and the Great Plains. Three-digit disagreements (Σ∆ = 3) occur most often in the SMAP vs. SoMo-L1 comparison (1% of land) and are concentrated in interior Australia, the Great Plains, and parts of northern Tibet.

To assess the consistency of the SM regime at different depths, we quantified spatial comparison in the subsurface SM regime across SoMo layers using dry-inclusive (${\triangle }_{{dry}}$), transitional-inclusive (${\triangle }_{{trans}}$), wet-inclusive (${\triangle }_{{wet}}$), and cumulative mismatch index (Σ∆). Full layer-pair maps and analyses of these comparisons are provided in the Supplementary (Supplementary Text 9, Supplementary Fig. 15).

### Performance of best-model fit to observed SM–EF

**Fig. 9:**
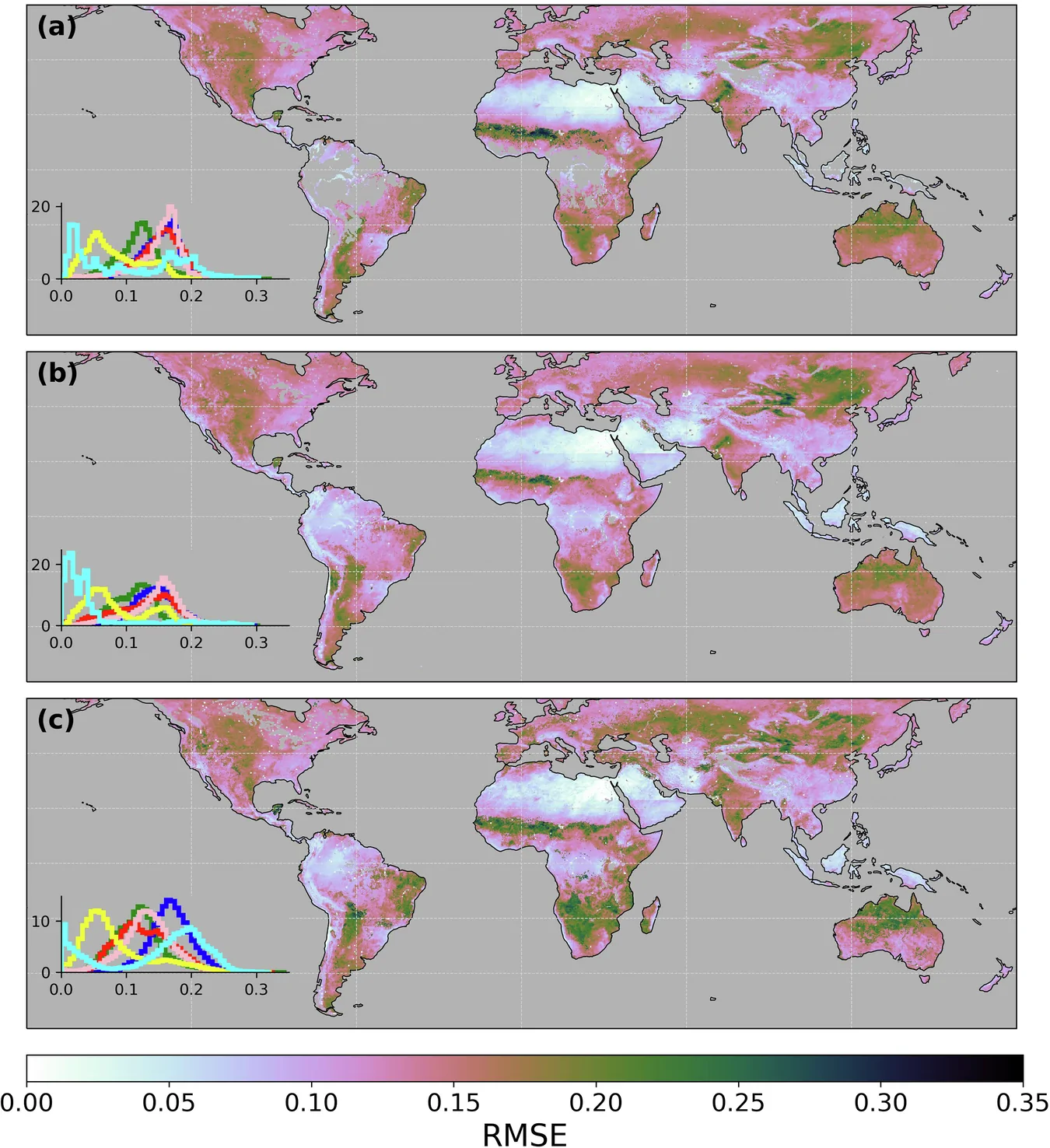
Global distribution of the goodness of fit between the most likely SM-EF candidate model and the actual SM: EF values at each grid cell, quantified as root mean square error (RMSE): (**a**) CCI-GLEAM, (**b**) SoMo-L1-GLEAM, and (**c**) SMAP-GLEAM. Each panel includes the PDFs (%) of RMSE across all grid cells identified for each segmented-linear regression model: green (001), blue (011), red (111), pink (010), yellow (110), and cyan (100). Gray areas indicate missing data.

The spatial pattern of fit quality, or how well segmented-linear regression models fit the data, is shown as the root mean square error (RMSE) between the best candidate model (selected based on the lowest BIC) and the actual EF values. Figure 9 displays the global distribution of RMSE between the most likely SM-EF candidate model and the actual SM: EF values for (a) CCI-GLEAM, (b) SoMo-L1-GLEAM, and (c) SMAP-GLEAM. Each panel contains the probability density function (PDF) (%) of RMSE across all grid cells identified for each segmented-linear regression model: green (001), blue (011), red (111), pink (010), yellow (110), and cyan (100).

Figure 9 demonstrates that there is a consistently large-scale error structure among the three SM regime combinations. For example, lower RMSE values (<0.1) are mostly observed in deserts (e.g., the Middle East and the Sahara), Southeast Asia, China, and tropical rainforests (only in SMAP and SoMo-L1 combinations). Conversely, high RMSE values (>0.2) are mostly found in the agricultural belts, particularly in the US, Eastern Europe, and India, across all three regime combinations. One reason that agricultural zones show high RMSE values is that GLEAM does not include irrigation water^131^; as a result, it cannot capture the irrigation effect on ET. Higher RMSE is also observed over other classical hotspot regions like the Sahel, north Australia, south Africa, Nordeste Brazil, Patagonia, and the Eurasian steppe in all regime combinations; however, SMAP-GLEAM exhibits higher RMSE values over these regions compared to CCI-GLEAM and SoMo-L1-GLEAM.

Additionally, the probability density of each segmented-linear regression model indicates how well the model fits across different regimes. Candidates 100 and 110 show peaks at lower RMSE in all three SM combinations; however, candidate 100 also displays a second peak at higher RMSE values in SMAP-GLEAM. Candidates 011, 111, and 010 consistently show higher RMSE and negatively skewed PDFs, demonstrating the difficulty of fitting transitional and agricultural regimes as described above. Generally, SMAP-GLEAM shows broader and right-tailed PDFs compared to CCI- and SoMo-L1-GLEAM. On the other hand, the RMSE between the best model candidate and the actual EF values across different SM layers of SoMo (subsurface SM regime) shows a similar spatial pattern, but more extensive high RMSE areas and sharper gradients appear as depth increases (Supplementary Fig. 16).

### Breakpoint uncertainty and selection sensitivity

**Fig. 10:**
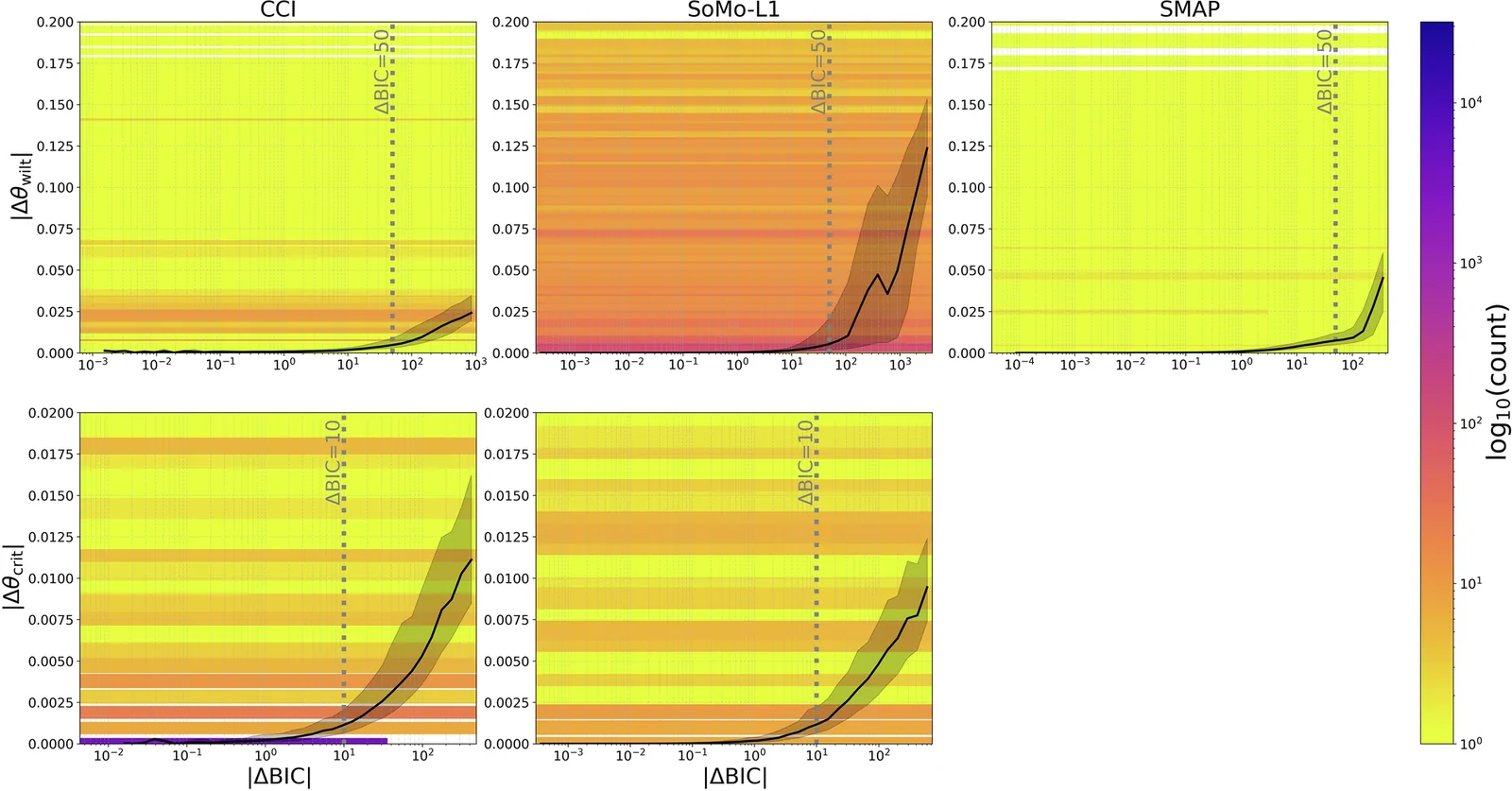
Hexbin plots showing the distribution of absolute differences in first- and second-best model breakpoints ($|\triangle {{\rm{\theta }}}_{{\rm{wilt}}}|$: first row, $|\triangle {{\rm{\theta }}}_{{\rm{crit}}}|$: second row) as a function of absolute differences in their BICs $(|\triangle {\rm{BIC}}|)$ for three SM products (CCI, SoMo-L1, SMAP; arranged from left to right). Black lines represent median values of $|\triangle {{\rm{\theta }}}_{{\rm{wilt}}}|$ and $|\triangle {{\rm{\theta }}}_{{\rm{crit}}}|$ calculated in logarithmically spaced bins of $|\triangle {\rm{BIC}}|$ with shaded areas indicating interquartile ranges (25th-75th percentiles). Vertical dotted lines indicate $|\triangle {\rm{BIC}}|$ thresholds ($|\triangle {\rm{BIC}}|$ = 50 for ${{\rm{\theta }}}_{{\rm{wilt}}}$, $|\triangle {\rm{BIC}}|$ = 10 for ${{\rm{\theta }}}_{{\rm{crit}}}$) used in breakpoint selection scenarios. The color bar represents the logarithm of point density (log₁₀ count). The $|\triangle {{\rm{\theta }}}_{{\rm{crit}}}|$ panel for SMAP is omitted due to insufficient data availability.

The regime analysis also provides estimates of ${\theta }_{{wilt}}$ and ${\theta }_{{crit}}$ across the globe based on observational data – a very valuable result for a range of applications from land surface model development to drought monitoring, forecast initialization, sub-seasonal predictions, and land-atmosphere feedback studies. However, often the regime models that include estimates of breakpoints do not provide the best fit to the available data. To determine actual breakpoint values when the best model does not provide them, we first need to understand how breakpoint fidelity relates to differences in BIC. In locations where both the first- and second-best models identify breakpoints, the absolute differences in their BICs ($\left|\triangle {BIC}\right|)$ and breakpoint values ($\left|\triangle {\theta }_{{wilt}}\right|\& \left|\triangle {\theta }_{{crit}}\right|)$ are calculated. The result is shown in Fig. 10, which displays the distribution of absolute differences in first- and second-best model breakpoints ($\left|\triangle {\theta }_{{wilt}}\right|$: first row, $\left|\triangle {\theta }_{{crit}}\right|$: second row) as a function of absolute differences in their BICs ($\left|\triangle {BIC}\right|)$ for three SM products (CCI, SoMo-L1, SMAP; arranged from left to right). GLEAM is excluded because it is a common EF product across all three combinations. The color bar represents the logarithm of point density (log₁₀ count), while the black lines represent median values of $|\triangle {\theta }_{{wilt}}|$ and $|\triangle {\theta }_{{crit}}|$ calculated in logarithmically spaced bins of $\left|\triangle {BIC}\right|$ with shaded areas indicating interquartile ranges (25th-75th percentiles). The $\left|\triangle {\theta }_{{crit}}\right|$ panel for SMAP is omitted due to insufficient data availability.

Figure 10 shows a strong relationship between differences in BIC scores and differences in breakpoints. Specifically, when the difference in information criteria is small, the breakpoints identified by the first- and second-best models are very close. This indicates that not only how reliable the breakpoint parameters are, but it also enables us to do quality and robustness checks by demonstrating that both ${\theta }_{{wilt}}$ and ${\theta }_{{crit}}$ are stable in all three combinations. This gives us the confidence that when the $\left|\triangle {BIC}\right|$ value is below a threshold, we may trust the breakpoint estimate from another model even when the first-best model breakpoint is undetermined. Therefore, we define the $\left|\triangle {BIC}\right|$ thresholds for ${\theta }_{{wilt}}$ and ${\theta }_{{crit}}$ to be 50 and 10, respectively. These thresholds are also valid for breakpoints from different SM layers of SoMo.ml (Supplementary Fig. 17). Using these thresholds in breakpoint selection scenarios (Supplementary Fig. 2), a more complete global distribution of ${\theta }_{{wilt}}$ and ${\theta }_{{crit}}$ is attainable.

### Cross-validation against *in-situ* measurements

To compare adjusted correlations with AmeriFlux *in-situ* correlations, we apply the Fisher Z transformation to each Pearson correlation coefficient and then compute the absolute Z score differences at each flux-tower site ($\left|\triangle Z\right|$). To determine whether the adjusted and *in-situ* correlations are significantly different, we compare $\triangle {\rm{Z}}$ with the two-tailed 1% p-value threshold of $\left|{\rm{Z}}\right|$= 2.576. If $\left|\triangle {\rm{Z}}\right|$ > 2.576, we reject the null hypothesis that the two correlations are not significantly different.

Since one of the main challenges when comparing gridded data with *in-situ* measurements is the spatial extent to which *in-situ* sites represent satellite-based grid cells^84,85^, we have additionally examined representativeness indices for AmeriFlux sites from supplementary data provided by Chu *et al*.^83^ available at (https://doi.org/10.5281/zenodo.4015350). Specifically, we used (1) the footprint-to-area representativeness based on daytime daily footprint climatology for the specified target area (LSP_ < target_area > _day_representativeness), and (2) the percentage of the dominant land cover type within the target area (LSP_ < target_area > _percentage). Given our grid resolution, we employed indices where “target area” corresponds to a 3,000 m radius around each flux tower.

**Fig. 11:**
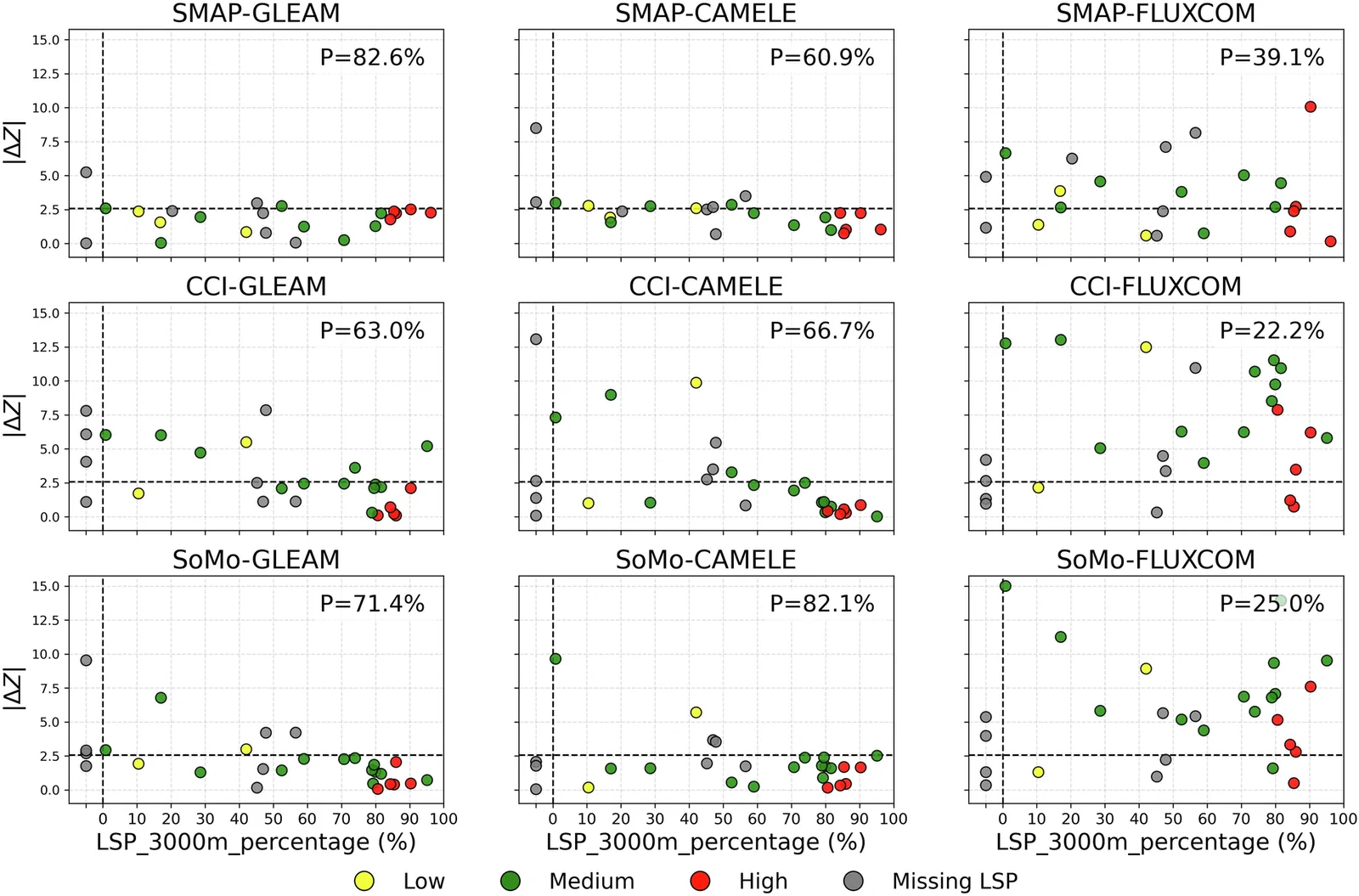
Scatter plots of absolute Z-score differences between the adjusted and AmeriFlux *in-situ* correlations of daily evaporation and SM anomalies, plotted against the percentage of the dominant land cover type within a 3000 m radius (LSP_3000m_percentage) on the x-axis. Each marker represents a flux-tower site; colors denote the footprint-to-area representativeness index based on daytime daily footprint climatology within a 3000 m radius (yellow = Low, green = Medium, red = High, grey = No reporting for area representativeness index). The dashed horizontal line indicates the two-tailed 1% p-value threshold |ΔZ| = 2.576; markers below this line indicate that the adjusted and *in-situ* correlations are not significantly different at α = 0.01. The markers on the left of the vertical line are sites where their LSP_3000m_percentage is not reported. The P in each panel shows the percentage of tower sites with correlations do not significantly differ from adjusted correlations at a p-value of 1%. Panels are organized by SM products (rows: SMAP, CCI, SoMo-L1) and evaporation products (columns: GLEAM, CAMELE, FLUXCOM).

Figure 11 shows the absolute Fisher-Z score differences (|ΔZ|) for the correlation between daily evaporation and SM anomalies at each flux-tower site. Across all combinations, correlations containing FLUXCOM as the ET product show the lowest percentage of sites where their adjusted correlation closely matches AmeriFlux correlations compared to other combinations of GLEAM and CAMELE (SMAP-FLUXCOM: 39.1%, CCI-FLUXCOM: 22.2%, SoMo-FLUXCOM: 25%). The poor performance of FLUXCOM combinations appears independent of area and land cover representativeness indices, as there is no pattern between the level of agreement and the high representativeness index.

In contrast, a high fraction of sites where adjusted correlations closely match AmeriFlux correlations occur in combinations of SMAP-GLEAM (82.6%), SoMo-CAMELE (82.1%), and SoMo-GLEAM (71.4%). For these combinations, nearly all sites with high land cover representativeness (red) are below the threshold line. Conversely, only a few sites with area representativeness index around 40%-60% have correlations that differ significantly from the adjusted correlations for SoMo-CAMELE and SoMo-GLEAM combinations.

Other combinations, such as SMAP-CAMELE, CCI-CAMELE, and CCI-GLEAM, demonstrate strong performance, with over 60% of sites showing adjusted correlation close to AmeriFlux. These three combinations show high agreement with AmeriFlux correlations when the area representativeness index is high. In other words, low agreement tends to occur at sites that less accurately reflect the aggregated land cover over the area represented by gridded data. In CCI-CAMELE and CCI-GLEAM combinations, disagreement sites are mainly those with low (yellow) or medium (green) land cover representativeness index.

Additionally, the analysis of the absolute Z-score differences between the adjusted and AmeriFlux *in-situ* correlations for daily sensible heat flux and SM anomalies (Supplementary Fig. 18, top row) shows good agreement, especially for SMAP-GLEAM and CCI-GLEAM, with 88.9% and 86.7% of sites, respectively, exhibiting adjusted correlations close to AmeriFlux. In contrast, SoMo-GLEAM demonstrates lower agreement, with only 57.1% of sites showing adjusted correlations near those of AmeriFlux. Meanwhile, all ${R}_{{Adjusted}}({SM},{EF})$ combinations exhibit strong agreement with AmeriFlux correlations at the 1% significance level (Supplementary Fig. 18, bottom row), with 88.9% of sites for SMAP–GLEAM, 86.7% for CCI–GLEAM, and 92.9% for SoMo–GLEAM. Supplementary Figs. 19-21 show the locations of the AmeriFlux sites where adjusted correlations are not significantly different from *in-situ* correlations, with a p-value of 1%, marked with a cross.

## Usage Notes

The absence of globally distributed *in-situ* flux tower observations results in an incomplete and uncertain understanding of land-atmosphere (L-A) coupling strength, which limits the credibility of global model assessments. To fill this gap, we have developed a global dataset of L-A coupling metrics to serve as benchmarks for diagnosing and correcting model errors as well as for model validation purposes. Specifically, we have produced a comprehensive global representation of the L-A coupling metrics based on observationally-based (largely from satellite data) gridded datasets, accounting for the effect of random observational errors present in satellite soil moisture (SM) measurements by employing a Markovian framework. These estimates enable us to derive mathematical formulations of the random error adjusted SM memory (SMM) and adjusted Pearson correlation coefficient (${R}_{{Adjusted}}$) between SM and surface fluxes (HF), including sensible heat flux (H), latent heat flux (LE), and evaporative fraction (EF). We have derived the adjusted terrestrial coupling index (${I}_{{Adjusted}}$) from ${R}_{{Adjusted}}$.

Since SM regimes are also important to identify when studying L-A coupling, we have fitted six candidate segmented regressions based on the SM-HF conceptual model proposed by Schwingshackl *et al*.^63^. Breakpoints such as wilting point ${\theta }_{{wilt}}$ and critical SM ${\theta }_{{crit}}$ have been estimated from these segmented regressions. ${\theta }_{{crit}}$ is important not only for those studying L-A interaction but also for a broader community concerned with weather forecasts, sub-seasonal predictions, climate projections, and model calibration. Knowledge of the distribution of ${\theta }_{{wilt}}$ is also of interest for forecast initialization as well as for the ecology, hydrology, and soil science communities, especially when studying extreme events like drought.

The datasets that we have used for the development of LA-coupling metrics include: two SM satellite datasets: SMAP and ESA CCI, a machine learning SM dataset: SoMo, and three surface heat flux datasets: GLEAM, CAMELE, and FluxCom-X-BASE. It should be noted that we also calculate these metrics across four different seasons: June-August (JJA), September-November (SON), December-February (DJF), and March-May (MAM), but their results are not shown here; they are part of the final datasets. Finally, we have assessed the accuracy of these adjusted metrics by comparing them with metrics calculated from AmeriFlux *in-situ* data. We also performed inter-product comparisons of the adjusted L-A coupling metric to determine the spatial discrepancies and whether they agree on L-A coupling characteristics.

Although this study offers valuable benchmarks for model validation, it faces several limitations. The primary limitation is in validating adjusted metrics against AmeriFlux *in-situ* measurements. Due to the sparse distribution of stations worldwide, the lack of FLUXNET2015 data after 2015 for SMAP validation, and issues with representativeness, only a subset of metrics and locations could be evaluated with *in-situ* data. It is important to note that the main motivation of this study was the limited *in-situ* data coverage for model validation of land-atmosphere interactions. Nevertheless, a high fraction of AmeriFlux stations with high representativeness of area and land cover type within a 3,000 m radius show adjusted correlations that are not significantly different from most gridded combinations (Fig. 11, Supplementary Fig. 18), especially for GLEAM- and CAMELE-based pairs. Beyond *in-situ* validation, the strong agreement in global patterns of adjusted metrics further enhances our confidence in gridded observational benchmarks.

Furthermore, even though this study only addresses the impact of random observational errors, which are often significant, other sources of noise and error in satellite products exist, such as systematic biases, scale mismatches, and spatial inconsistencies^70,132^. Additionally, the Markovian framework used to estimate random errors in this study has its own limitations. This framework assumes stationary meteorological forcing, such as precipitation and radiation, and ignores persistence in the meteorological forcing^51^. In reality, seasonal variations and land–atmosphere feedback can extend or truncate SMM. For instance, wet soils can enhance evaporation, induce additional precipitation, and thereby prolong anomalies. As a result, there are uncertainties in accurately capturing random error with this linear approach.

The other limitation of this study is the lack of uncertainty estimates or quality indicators for SM breakpoints, which could enhance their transparency and usability, especially because these diagnostic breakpoints might be used in subsequent analyses and forecasting applications. Optimally, the uncertainty in these breakpoints can be quantified using a bootstrap framework applied independently at each grid cell. In this method, the paired SM and EF observations could be resampled to maintain temporal dependence. The full set of candidate segmented-regression models could be refitted for each replicate, model selection could be repeated using the same BIC-based criteria as in the main analysis, and finally, SM breakpoints (${\theta }_{{wilt}}$ and ${\theta }_{{crit}}$) could be recalculated from the selected model. Repeating this process across many replicates would yield empirical distributions for the breakpoints, from which confidence intervals and model-selection stability metrics could be derived.

However, implementing this procedure globally for the current dataset is computationally very demanding. Our analysis uses daily SM and EF data from 1980-2023 for CCI-GLEAM, March 2015-April 2023 for SMAP-GLEAM, and 2000-2019 for SoMo-GLEAM at a global scale. Breakpoint estimation at each grid cell requires fitting multiple candidate models, evaluating BIC and RMSE for each candidate, and applying hierarchical breakpoint-selection logic. In the current workflow, each valid grid cell is screened using minimum sample-size thresholds, exclusion of cells dominated by repeated maximum SM values, physically constrained breakpoint ranges, and minimum regime-support requirements before a candidate fit is accepted. The final breakpoint fields are then selected through a multi-step hierarchy based on the best and second-best BIC-ranked models. A bootstrap analysis would need to repeat this entire pipeline hundreds or thousands of times for every grid cell, which becomes prohibitively expensive at 0.25° and especially 0.1° spatial resolution. As a result, although a comprehensive global bootstrap-based breakpoint uncertainty analysis is not feasible in this study, quality flags for breakpoints have been added in the dataset that indicate the selection step in the hierarchical breakpoint-selection framework described in the Methods section.

Future research should incorporate these nonlinear feedback and systematic uncertainties to refine error estimates and improve the accuracy of L-A coupling metrics. Information theory provides an alternative approach to capture nonlinear error characteristics without requiring ancillary or independent data. This method uses metrics such as entropy and complexity to quantify the randomness and patterns in time series instead of relying on linear correlations. In other words, Shannon entropy^133^ measures the level of unpredictability (randomness) in the time series, while high complexity indicates that the data contain rich, structured variations beyond simple linear dynamics. Recent studies have applied such metrics to satellite SM to analyze patterns and uncertainties inherent in the satellite observations^134^. Consequently, information-theoretic metrics can be useful for improving error quantification beyond linear assumptions and for capturing linear and nonlinear relationships between variables, as well as analyzing causal relationships in nonlinear systems^135,136^.

This study provides the first global observationally-based gridded land–atmosphere coupling metrics that adjust for random retrieval errors in SM satellite measurements. The analysis shows that (1) large-scale SM–E correlations are stable across data products and align with established hydroclimatic theory; (2) the choice of SM product explains most of the spatial variance in the correlation fields, highlighting SM observational uncertainty as the main constraint on L–A coupling diagnostics; (3) when flux variability is incorporated to form the coupling index, flux product characteristics predominantly influence the inter-product spread; (4) inter-product consensus in transitional and wet regimes identifies high-confidence regions for benchmarking and sharp transition zones, whose locations can serve as stern tests for land-surface and coupled models; and (5) although cross-product inconsistency in SM memory raises uncertainty in using these products for SMM metrics, the percentile maps are useful as indicators of short- or long-term memory for model validation.

Therefore, these improved metrics will enhance the development of more accurate climate models and reanalysis products. By analyzing the adjusted correlations and identifying various global regimes and hotspots for land-atmosphere interactions, these observational metrics not only help to clarify the dynamics of SM and energy availability but also set the stage for exploring broader climatic questions. For instance, understanding the spatial relationships with vegetation could reveal how different types of vegetation influence, or are affected by, SM and energy distribution patterns. These insights could significantly improve our understanding of ecosystem dynamics, climate variability, and the potential implications for global climate model simulations, targeting areas for improvement in land surface physics, canopy hydraulics, and soil parameterizations. Future research could combine these regime maps with emergent constraint techniques to refine parameterizations in climate models and reanalysis products. There is also great potential to use information-theoretic metrics or machine learning error decomposition to identify the remaining nonlinear retrieval errors.

## Supplementary information

Supplementary Information

## Data Availability

The datasets generated during the current study, along with a README file, are freely available in the Zenodo repository under a CC BY 4.0 license (https://doi.org/10.5281/zenodo.17603859). A detailed description of the dataset archives (“coupling”, “SMM”, and “regime”) and their variables is provided in both the Data Records section of the manuscript and the README file in the Zenodo repository.

## Code availability

The Python code used to generate the datasets in this article is available in public repositories at Zenodo (https://doi.org/10.5281/zenodo.17702299) and GitHub (https://github.com/nTavakoli/Global-Observationally-Based-Land-Atmosphere-Coupling-Metrics-Code.git).
