# Supplementary material for: A global dataset of land-atmosphere coupling metrics based on observations for evaluating Earth system models
Source: Sci Data. 2026 Jun 12;13:1354. doi: 10.1038/s41597-026-07519-2 (PMC13616915; doi:10.1038/s41597-026-07519-2)
Supplement: Supplementary file 1 [file 41597_2026_7519_MOESM1_ESM.pdf]

Supplementary Materials for

A global dataset of land–atmosphere coupling metrics based on observations for evaluating Earth system models

- Text 1. Cumulative distribution function (CDF) of the relative random error (δ/σ) in observed SM.
- Text 2. Hierarchical clustering dendrograms based on 1- Spearman rank correlations.
- Text 3. Pairwise Spearman rank correlations among $I_{Adjusted}$ and $R_{Adjusted}$ for SM-EF and SM-H.
- Text 4. Spatial distribution of CV in Fisher z-space for $R_{Adjusted}(SM, H)$ and $R_{Adjusted}(SM, EF)$.
- Text 5. Cumulative distribution of the coefficient of variation (CV) in Fisher z-space among nine $R_{Adjusted}(SM, E)$ pairs.
- Text 6. Global maps of percentile differences in adjusted soil moisture memory.
- Text 7. Theoretical equation for |ΔCDF| of SMM in two random time series.
- Text 8. Derivation of the area between the random and perfect-model CDF curves.
- Text 9. Spatial consistency of subsurface SM regimes.

- Figure 1. Sensitivity test for estimating random observational error variance from the SMAP time series at the ARM site (97.52 °W, 36.59 °N) during JJA.
- Figure 2. Schematic overview of breakpoint selection scenarios.
- Figure 3. Cumulative distribution function (CDF) of the relative random error (δ/σ) in observed SM.
- Figure 4. Hierarchical clustering dendrograms based on 1- Spearman rank correlations for $R_{Adjusted}(SM, E)$ and $I_{Adjusted}(SM, E)$.
- Figure 5. Pairwise Spearman rank correlations among $I_{Adjusted}$ and $R_{Adjusted}$ for SM-EF and SM-H.
- Figure 6. Hierarchical clustering dendrograms based on 1- Spearman rank correlations for $R_{Adjusted}(SM, EF)$ and $I_{Adjusted}(SM, EF)$.
- Figure 7. Hierarchical clustering dendrograms based on 1- Spearman rank correlations for $R_{Adjusted}(SM, H)$ and $I_{Adjusted}(SM, H)$.
- Figure 8. Global distribution of the adjusted correlation between daily evaporation and soil moisture anomalies, $R_{Adjusted}(SM, E)$, derived from three soil moisture products paired with three evaporation products. White areas indicate grid cells where $R_{Adjusted}(SM, E)$ is not significant at the 5% significance level (two-sided Student's t-test; $p \geq 0.05$).
- Figure 9. Same as in Figure 8, but white indicates grid cells where $R_{Adjusted}(SM, EF)$ is not significant at the 5% significance level (two-sided Student's t-test; $p \geq 0.05$).

- Figure 10. Same as in Figure 8, but white indicates grid cells where $R_{Adjusted}(SM,H)$ is not significant at the 5% significance level (two-sided Student's t-test; $p \geq 0.05$).
- Figure 11. Global distribution of CV in Fisher z-space among $R_{Adjusted}(SM,H)$ pairs.
- Figure 12. Global distribution of CV in Fisher z-space among $R_{Adjusted}(SM,EF)$ pairs.
- Figure 13. Cumulative distribution of the coefficient of variation (CV) in Fisher z-space among nine $R_{Adjusted}(SM,E)$ pairs.
- Figure 14. Global maps of percentile differences in adjusted soil moisture memory.
- Figure 15. Spatial consistency of subsurface SM regime classifications between SoMo product pairs: SoMo-L1 vs. SoMo-L2, SoMo-L2 vs. SoMo-L3, SoMo-L1 vs. SoMo-L3.
- Figure 16. Global distribution of the root mean square error (RMSE) between the most likely SM-EF candidate model and the actual EF values across different SM layers of SoMo.ml: (a) SoMo-L1-GLEAM, (b) SoMo-L2-GLEAM, and (c) SoMo-L3-GLEAM.
- Figure 17. Hexbin plots showing the distribution of absolute differences in first- and second-best model breakpoints ($|\Delta\theta_{wilt}|$: first row, $|\Delta\theta_{crit}|$: second row) as a function of absolute differences in their BICs ($|\Delta BIC|$) for different SM layers of SoMo.ml (SoMo-L1, SoMo-L2, SoMo-L3; arranged from left to right).
- Figure 18. Scatter plots of absolute Z-score differences between the adjusted and AmeriFlux in-situ correlations of daily sensible heat flux–soil moisture anomalies (first row) and daily evaporative fraction–soil moisture anomalies (second row).
- Figure 19. Spatial distribution of the adjusted correlation between daily evaporation and soil moisture anomalies, $R_{Adjusted}(SM,E)$, at AmeriFlux sites. In each panel, a cross (+) mark indicates AmeriFlux sites where adjusted correlations are not significantly different from in-situ correlations, with a p-value of 1%.
- Figure 20. Spatial distribution of the adjusted correlation between daily evaporative fraction and soil moisture anomalies, $R_{Adjusted}(SM,EF)$, at AmeriFlux sites. In each panel, a cross (+) mark indicates AmeriFlux sites where adjusted correlations are not significantly different from in-situ correlations, with a p-value of 1%.
- Figure 21. Spatial distribution of the adjusted correlation between daily sensible heat flux and soil moisture anomalies, $R_{Adjusted}(SM,H)$, at AmeriFlux sites. In each panel, a cross (+) mark indicates AmeriFlux sites where adjusted correlations are not significantly different from in-situ correlations, with a p-value of 1%

- Table 1. Site general information for the AmeriFlux sites used in the study.

Text 1. Cumulative distribution function (CDF) of the relative random error (δ/σ) in observed SM

Figure 3 shows the CDF of the relative random error (δ/σ) for SoMo-L1 (blue), SMAP (green), and CCI (red). According to this figure, 90% of grid cells in SoMo-L1 have a random error fraction close to zero compared to the total SM standard deviation; only about 10% have a random error larger than 5% of the total standard deviation. Conversely, 12% of SMAP grid cells have a zero relative random error, while 88% have a significant relative error, with 20% of grid cells showing an error ratio above 0.24. For CCI, 5% of the points have a random error ratio exceeding 0.38572.

Text 2. Hierarchical clustering dendrograms based on 1- Spearman rank correlations

To reveal how the adjusted correlation or terrestrial coupling combinations group together based on their spatial correlation patterns, we generated hierarchical dendrograms using the distance metric: $D = 1 - \text{Spearman } R$. Hierarchical clustering for the adjusted correlation coefficient $R_{Adjusted}(SM, E)$ and the adjusted terrestrial coupling index $I_{Adjusted}(SM, E)$ are shown in Figure 4.

The nine $R_{Adjusted}(SM, E)$ combinations (Figure 4a) form three main clusters. First, CCI-based and SoMo-based pairs create tight groups, with inter-cluster distances of only ≈ 0.05–0.10. Notably, CCI-GLEAM and SoMo-GLEAM form the tightest subgroup. Next, tight clusters of CCI-FLUXCOM with SoMo-FLUXCOM and CCI-CAMELE with SoMo-CAMELE merge at a slightly larger distance (D ≈ 0.15). Meanwhile, SMAP-GLEAM joins the CCI-GLEAM and SoMo-GLEAM sub-cluster at D ~ 0.10; then the combined GLEAM branch connects with the cluster containing CCI-FLUXCOM, SoMo-FLUXCOM, CCI-CAMELE, and SoMo-CAMELE at D ≈ 0.15–0.20. Finally, the two outliers, SMAP-FLUXCOM and SMAP-CAMELE, only connect at D ~ 0.25, highlighting their distinct spatial behavior.

Figure 4b demonstrates that the clustering pattern among nine $I_{Adjusted}(SM, E)$ combinations is influenced by ET products in a way that each of these ET products forms its own cluster. Notably, CCI-ET and SoMo-ET form the closest cluster, and SMAP-ET joins them as the next cluster. As a result, GLEAM- and CAMELE combinations create the same cluster (D <0.25), while FLUXCOM-based combinations form the most distinct cluster, separated by the largest distances (D ≈0.25).

Further analysis of $R_{Adjusted}(SM, EF)$ and $I_{Adjusted}(SM, EF)$ in Figure 6 and $R_{Adjusted}(SM, H)$ and $I_{Adjusted}(SM, H)$ in Figure 7 shows that CCI-GLEAM and SoMo-GLEAM pairs have shorter horizontal distances in their diagrams, indicating stronger similarity between them. SMAP-GLEAM then joins the cluster of CCI-GLEAM and SoMo-GLEAM. This distance is the shortest among $I_{Adjusted}(SM, EF)$ clusters (D ~0.04- 0.06) and is then longest in $R_{Adjusted}(SM, H)$ clusters (D ~0.1-0.2).

Text 3. Pairwise Spearman rank correlations among $I_{Adjusted}$ and $R_{Adjusted}$ for SM-EF and SM-H

Pairwise Spearman rank correlations among the $I_{Adjusted}$ combinations are robust, exceeding 0.92 for the SM–EF combinations (Figure 5a: upper triangle) and ≥ 0.85 for the SM–H combinations (Figure 5b: upper triangle). Pairwise correlations among the $R_{Adjusted}$ combinations are also strong, with ≥ 0.88 for SM–EF (Figure 5a: lower triangle) and ≥ 0.77 for SM–H (Figure 5b: lower triangle).

Text 4. Spatial distribution of CV in Fisher z-space for $R_{Adjusted}(SM,H)$ and $R_{Adjusted}(SM,EF)$

According to Figure 11, nearly the entire globe appears in light blue (near-zero CV), which indicates regions with strong agreement in $R_{Adjusted}(SM,H)$. However, high uncertainty exists over the western U.S., the Middle East, North Africa, Central Asia, and parts of Europe. In these regions, blue shading corresponds to negative CV values, suggesting that most products consistently report negative correlations, although they differ significantly in magnitude. Conversely, regions shaded in red display positive CV values, indicating that the majority of products detect positive correlations, but their magnitude differs substantially.

Figure 12 also displays the CV distribution in Fisher z-space for $R_{Adjusted}(SM,EF)$ across the same combinations. This figure generally shows strong agreement between products in both sign and magnitude; however, northeastern Africa stands out with strongly positive CV values. This may indicate either significant uncertainty in this region or a very low mean correlation that causes the CV to appear higher. Conversely, the blue regions, primarily located in South and Southeast Asia, Western Europe, and North America, show greater dispersion in negative CV values.

Text 5. Cumulative distribution of the coefficient of variation (CV) in Fisher z-space among nine $R_{Adjusted}(SM,E)$ pairs

To distinguish regions dominated by either energy- or water-limited coupling regimes, along with three levels of agreement across nine $R_{Adjusted}(SM,E)$ combinations, a cumulative distribution function (CDF) of CV in Fisher-z space is computed (Figure 13). Empirical thresholds based on the 15th, 30th, 60th, and 85th percentiles of this CDF are then used to classify different coupling regime types, such as energy- and water-limited. Regions where the CV is less than or equal to the 15th percentile are classified as low-energy-limited, indicating low agreement among products with predominantly negative coupling values. CV values between the 15th and 30th percentiles are classified as medium-energy-limited, while those between the 30th percentile and zero are categorized as high-energy-limited, still within the negative CV range but exhibiting less variability. For positive CV values, those between 0 and the 60th percentile are considered high-water-limited, indicating strong agreement in positive coupling. Areas with CV values between the 60th and 85th percentiles are classified as medium-water-limited, and regions exceeding the 85th percentile are labeled as low-water-limited, representing the highest variability among products in the positive coupling regime. The CV thresholds for these percentiles are –1.26, –0.57, 0.36, and 0.93, respectively.

Text 6. Global maps of percentile differences in adjusted soil moisture memory

To identify areas of agreement and disagreement between each pair of SMM fields, we generated three pairwise percentile-difference maps as shown in Figure 14. This figure displays the grid-by-grid percentile differences between the adjusted SMM derived from three SM products for 2015-2019 at 0.25° spatial resolution.

In Figure 14a, percentile differences between SoMo-L1 and CCI are small, showing that in most areas both products place each grid cell at approximately the same position in their own global SMM distributions. However, the largest positive discrepancies, where SoMo-L1 assigns longer SMM than CCI, occur in northern Eurasia, large parts of Europe, Tibet, Canada, and Alaska. On the other hand, North Africa and central to eastern Iran show pronounced negative differences, suggesting that CCI tends to overestimate SMM relative to SoMo-L1 in these regions.

Figure 14b shows the differences in percentiles between the adjusted SMM from SoMo-L1 and SMAP. SoMo-L1 identifies higher SMM values in Eurasia, certain areas of Europe and southern Africa, Tibet, Patagonia, western Canada, and much of inland and southern Australia. In contrast, SMAP tends to indicate longer memory in densely vegetated regions such as the Congo Basin, Amazon Basin, China, Southeast Asia, and the Maritime Continent. Additionally, blue areas, indicating that SMAP detects longer memory than SoMo-L1, are prominent in central to northern Africa and central to eastern Iran.

Finally, Figure 14c compares the percentile ranks of adjusted SMM from CCI with those from SMAP (CCI–SMAP). Overall, CCI and SMAP exhibit the highest similarity in ranking SMM within their own distributions, compared to their respective comparisons to SoMo-L1. Despite this consistency, CCI assigns longer SMM in the Eurasian steppe, Sahara, Mediterranean rim, southern Africa, and interior Australia, while SMAP perceives longer memory than CCI across China, northeastern Canada, and Siberia.

Text 7. Theoretical equation for |ΔCDF| of SMM in two random time series

If we have two independent white noise time series, their CDF will have a continuous uniform distribution:

$$U_1, U_2 \sim Uniform(0,1) \tag{1}$$

Then, the absolute CDF difference (|ΔCDF|) will be,

$$Z \equiv |U_1 - U_2| \in [0,1] \tag{2}$$

Since (U_1, U_2) is uniform on the unit square, $\{|U_1 - U_2| \leq z\}$ corresponds to the unit square with two corner right triangles of side length $1 - z$ removed. Each triangle has an area of $\frac{1}{2}(1 - z)^2$. Thus, for $0 \leq z \leq 1$,

$$F_Z(z) \equiv Pr(Z \leq z) = 1 - \left(2 * \frac{1}{2}(1 - z)^2\right) = 1 - (1 - z)^2 = 2z - z^2 \tag{3}$$

Differentiating gives the PDF:

$$f_Z(z) = \frac{d}{dz} F_Z(z) = 2 - 2z,\ \ 0 \leq z \leq 1 \tag{4}$$

Therefore, under the white-noise independence null, the absolute difference between the SMM-based CDF values from two series has:

$$F_Z(z) = 2z - z^2, \quad f_Z(z) = 2 - 2z, \qquad 0 \leq z \leq 1 \tag{5}$$

Text 8. Derivation of the area between the random and perfect-model CDF curves

A perfect model concentrates all mass at $Z = 0$, so its CDF is $F_{perfect}(z) = 1$ for any $z > 0$. Therefore, the area between the random and perfect-model CDF curve on $[0,1]$ is:

$$A_{max} = \int_0^1 \left(F_{perfect}(z) - F_Z(z)\right) = \int_0^1 \left(1 - (2z - z^2)\right)dz = \int_0^1 (1 - 2z + z^2)dz = \left[z - z^2 + \frac{z^3}{3}\right] = \frac{1}{3} = 0.333 \quad (6)$$

Text 9. Spatial consistency of subsurface SM regimes

To identify spatial disagreement among detected subsurface SM regimes, Figure 15 presents comparisons of dry-inclusive (Δ_{dry}), transitional-inclusive (Δ_{trans}), and wet-inclusive (Δ_{wet}) regimes across different layers of the SoMo SM product: SoMo-L1 vs. SoMo-L2, SoMo-L2 vs. SoMo-L3, and SoMo-L1 vs. SoMo-L3. GLEAM is excluded from the labels as it is the shared EF dataset. Rows 1–3 in Figure 15 correspond to Δ_{dry}, Δ_{trans}, and Δ_{wet}, respectively, while the bottom row shows the cumulative mismatch index ($\Sigma\Delta$). Columns, from left to right, correspond to the layer pairs L1–L2, L2–L3, and L1–L3. Grey pixels indicate perfect agreement, while colored pixels highlight grid cells where only one product assigns the cell to the respective regime. Inset histograms show the land-area fraction of each agreement or disagreement category. Cells without valid data in both products are masked in white.

According to Δ_{dry} panels (Figure 15a-c), SoMo-L1 vs. SoMo-L2 shows the strongest agreement in detecting the locations of the dry-inclusive regime (≈ 71 % of all dry pixels), whereas SoMo-L2 vs. SoMo-L3 and SoMo-L1 vs. SoMo-L3 show lower agreements (64.9% and 60.1%, respectively). Based on these panels, strong agreements in identifying Δ_{dry} are mainly observed in the Sahara, the Middle East, interior and western Australia, the Horn of Africa, the southwestern U.S., some patches in northern Brazil, and Central Asia. The area of disagreement in detection of Δ_{dry} is also very similar across Δ_{dry} panels (Figure 15a-c); however, SoMo-L2 vs. SoMo-L3 (Figure 15b) and SoMo-L1 vs. SoMo-L3 (Figure 15c) show more red cells across southern Australia and the Sahel.

Consistent with the dry-inclusive analysis, the highest agreement in detecting the transitional-inclusive regime occurs in the SoMo-L1 vs. SoMo-L2 comparison (84.7%; Figure 15d), followed by SoMo-L2 vs. SoMo-L3 (79.7%; Figure 15e), while the lowest agreement appears for SoMo-L1 vs. SoMo-L3(72%; Figure 15f). Spatially, all three Δ_{trans} panels (Figure 15d-f) show similar regions of agreement in detecting Δ_{trans} (gray cells); however, SoMo-L1 vs. SoMo-L2 shows more agreement in detecting Δ_{trans} in much of the eastern Eurasian steppe and the western Sahara compared to SoMo-L2 vs. SoMo-L3 and SoMo-L1 vs. SoMo-L3 comparisons.

The pattern of disagreement in Δ_{trans} for SoMo-L1 vs. SoMo-L2 is mostly observed in scattered patches across the western Sahara and northeastern Eurasia steppe (Figure 15d). By contrast, SoMo-L3 appears to detect fewer Δ_{trans} in the Arabian deserts compared to SoMo-L1 and SoMo-L2, as evidenced by the more purple cells in the SoMo-L2 vs. SoMo-L3 and SoMo-L1 vs. SoMo-L3 panels (Figure 15e and f). Additionally, Figure 15f shows more disagreements in detecting Δ_{trans} across the western Sahara, eastern Eurasian steppe, and some parts of Central Asia.

The pairwise comparison of Δ_{wet} (Figure 15g-i) reveals that SoMo-L1 vs. SoMo-L2 shows the highest agreement in identifying Δ_{wet}, with approximately 83% of all wet pixels. In contrast SoMo-L2 vs. SoMo-L3 and SoMo-L1 vs. SoMo-L3 show lower agreement rates of 81.7% and

73.5%, respectively. Similar to Figure 15g-i, all three Δ_{wet} panels display a consistent spatial pattern of mismatch pixels, where only one product identifies the water-limited regime. These mismatches are scattered but mainly found over western and central North America, the Sahel, Patagonia, the Eurasian forest-steppe, the Middle East, and southern Australia.

According to $\Sigma\Delta$ panels (Figure 15j-l), SoMo-L1 vs. SoMo-L2 shows the highest agreement in detecting the full set of dry-, transitional-, and wet-inclusive regimes, with 74.4% of land area having $\Sigma\Delta = 0$. This is followed by SoMo-L2 vs. SoMo-L3 and SoMo-L1 vs. SoMo-L3, with agreement rates of 73.7% and 61.3%, respectively. On the other hand, the area fraction of single-digit disagreements ($\Sigma\Delta = 1$) is about 20.4% for SoMo-L1 vs. SoMo-L2, 19.5% for SoMo-L2 vs. SoMo-L3, and 28.2% for SoMo-L1 vs. SoMo-L3.

Figure 15j-l also show that the pattern of single-digit disagreements ($\Sigma\Delta = 1$) is scattered but mostly dominant in the Sahara, the Middle East, southern Africa, Australia, northern Eurasia, India, Southeast Asia, and both North and South America. In contrast, two-digit conflicts ($\Sigma\Delta = 2$) appear as scattered patches mainly across the Eurasian steppe, North Africa, the Middle East, western U.S., Patagonia, Central Asia, and interior Australia. The area fraction of two-digit disagreements is 5.1% for SoMo-L1 vs. SoMo-L2, 6.6% for SoMo-L2 vs. SoMo-L3, and 10.1% for SoMo-L1 vs. SoMo-L3, while three-digit conflicts ($\Sigma\Delta = 3$) rarely exceed one-eighth of the global land surface in all three comparisons.

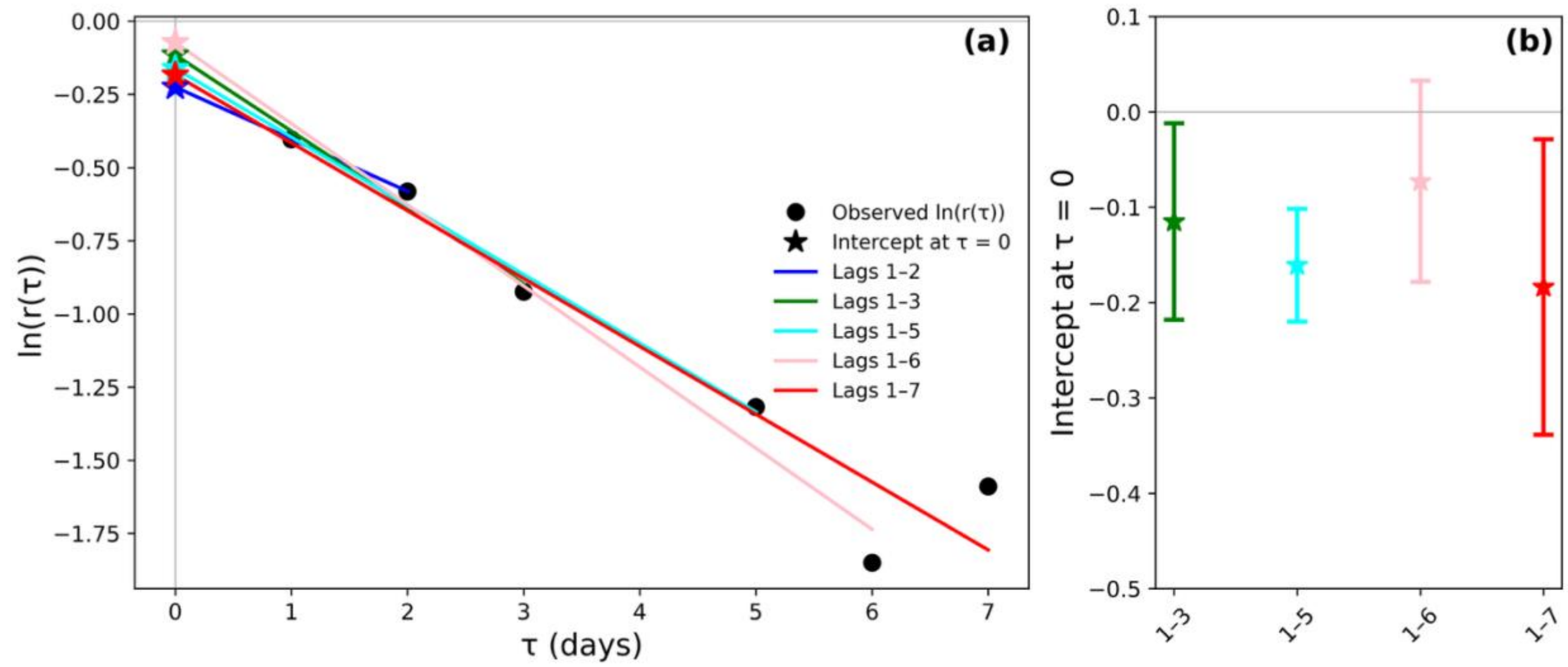

Figure 1. Sensitivity test for estimating random observational error variance from the SMAP time series at the ARM site (97.52 °W, 36.59 °N) during JJA. (a) Observed logarithm of the ACF values, shown as $\left(ln\left(r(\tau)\right)\right)$, are plotted as black circles for lags 1-7 days. Colored straight lines show fits obtained using different lag windows (lags 1–2, 1–3, …, 1–7), and colored stars at $\tau = 0$ indicate the extrapolated intercept from each fit. (b) Corresponding intercept estimates at $\tau = 0$ for each lag-window choice, with error bars showing the standard error of the fitted intercept. The intercept at $\tau = 0$ provides an estimate of the displacement d, from which α is calculated and subsequently used to estimate the variance of observational random error according to equation (4). Note that lag 4 is missing due to SMAP values being missing every 4 days.

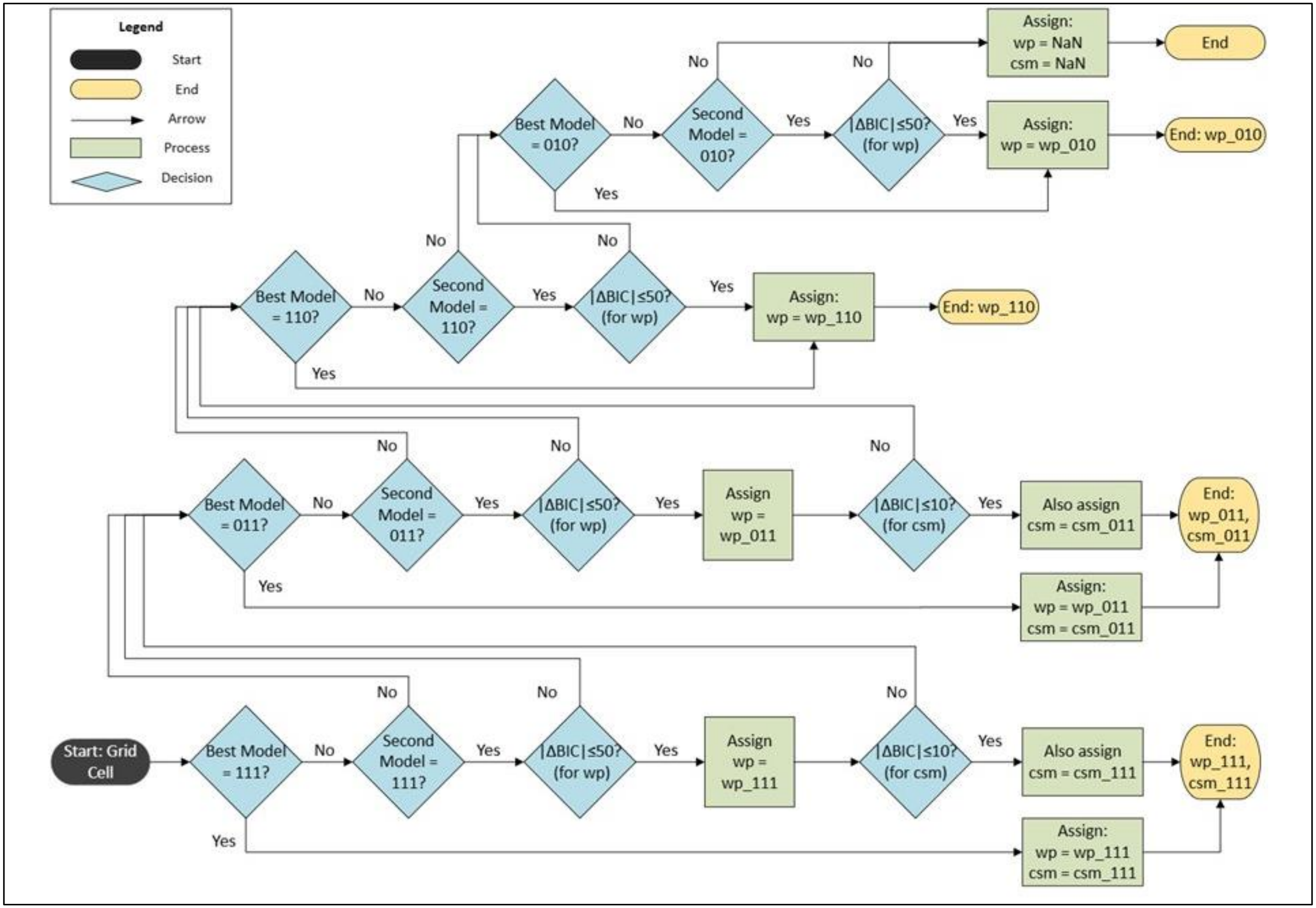

Figure 2. Schematic overview of breakpoint selection scenarios.

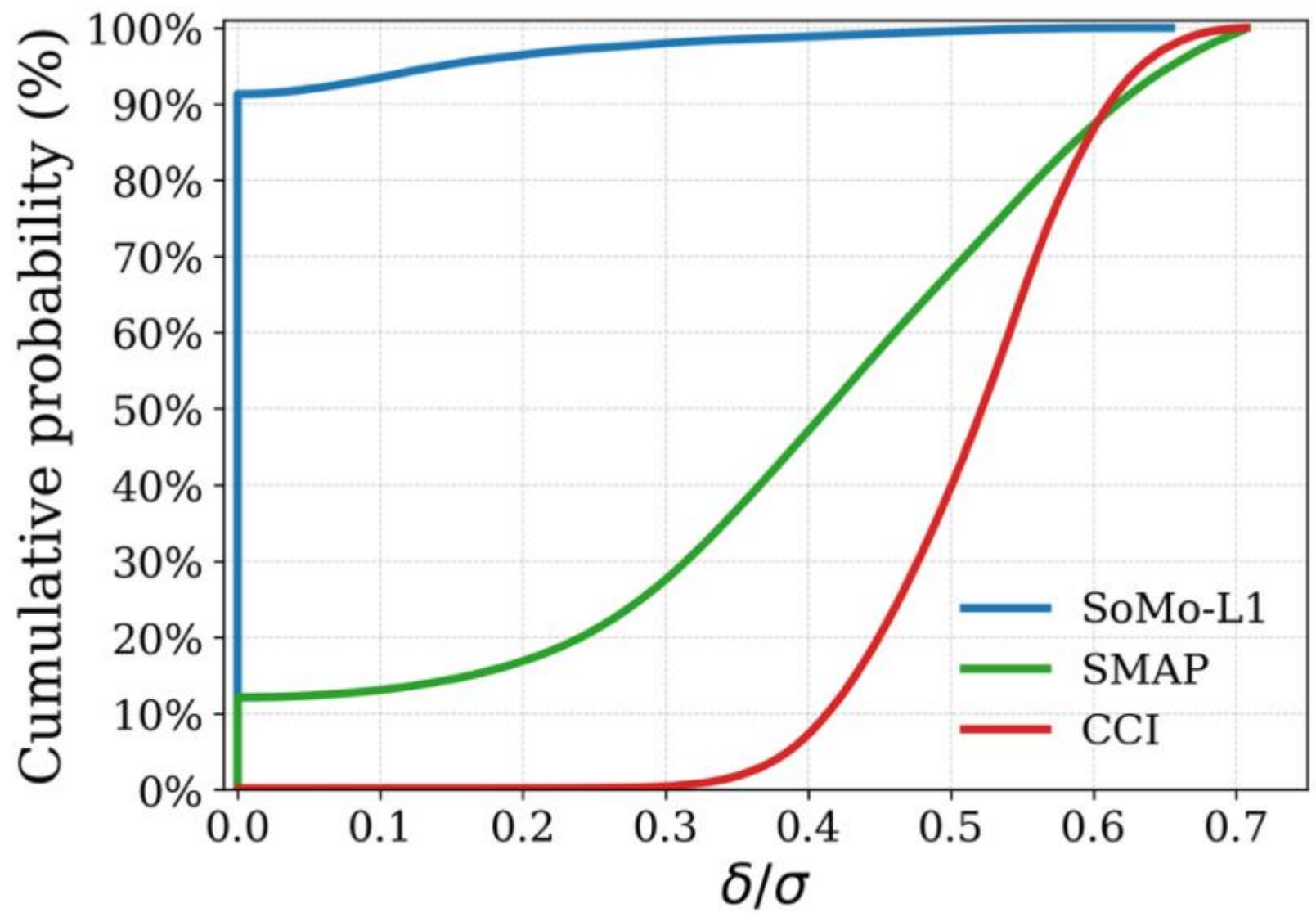

Figure 3. Cumulative distribution function (CDF) of the relative random error (δ/σ) in observed SM for SoMo-L1 (blue), SMAP (green), and CCI (red).

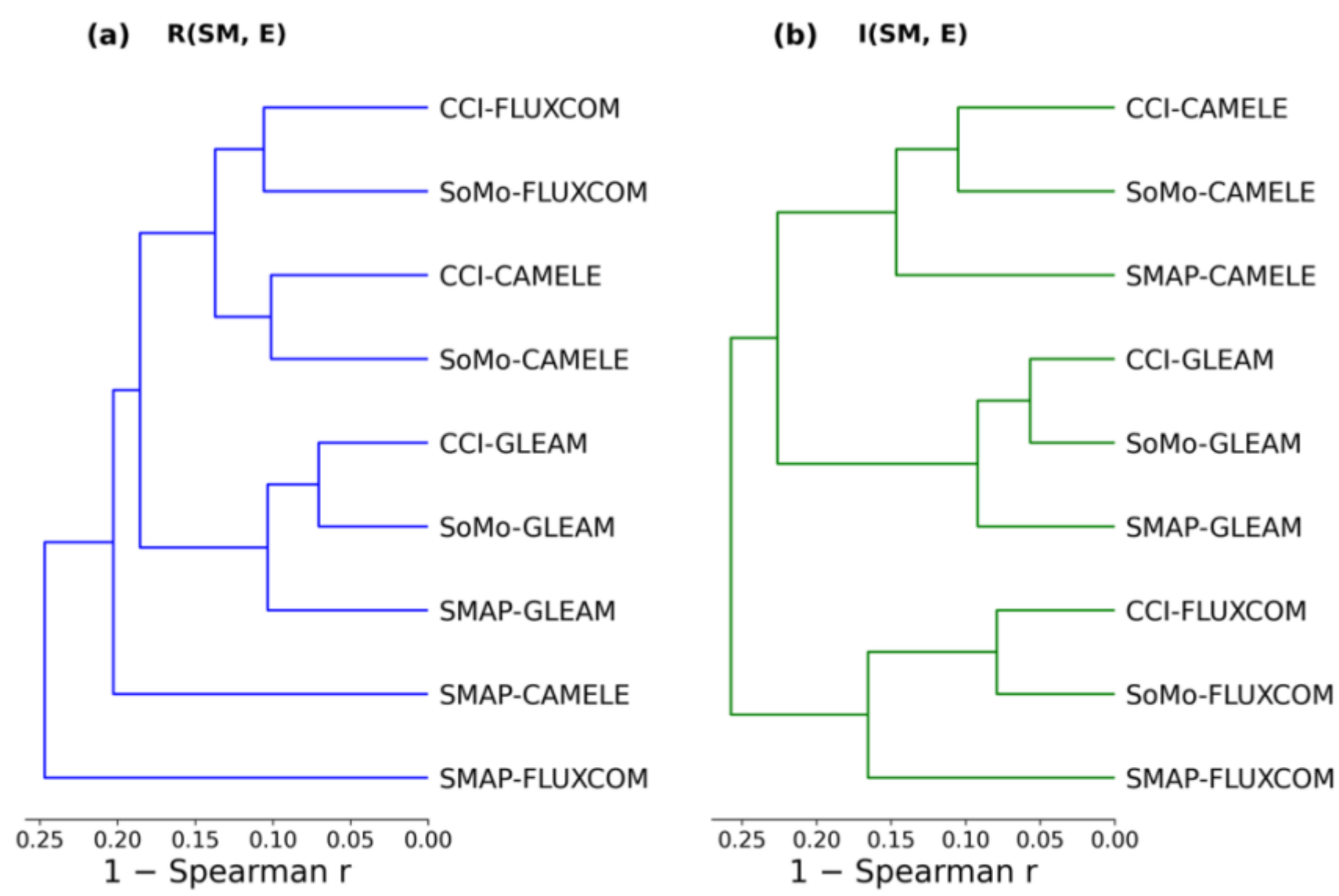

Figure 4. Hierarchical clustering dendrograms based on 1- Spearman rank correlations for (a) $R_{Adjusted}(SM, E)$ and (b) $I_{Adjusted}(SM, E)$, using combinations of soil moisture products (SMAP, SoMo, CCI) and evaporation datasets (GLEAM, CAMELE, FLUXCOM).

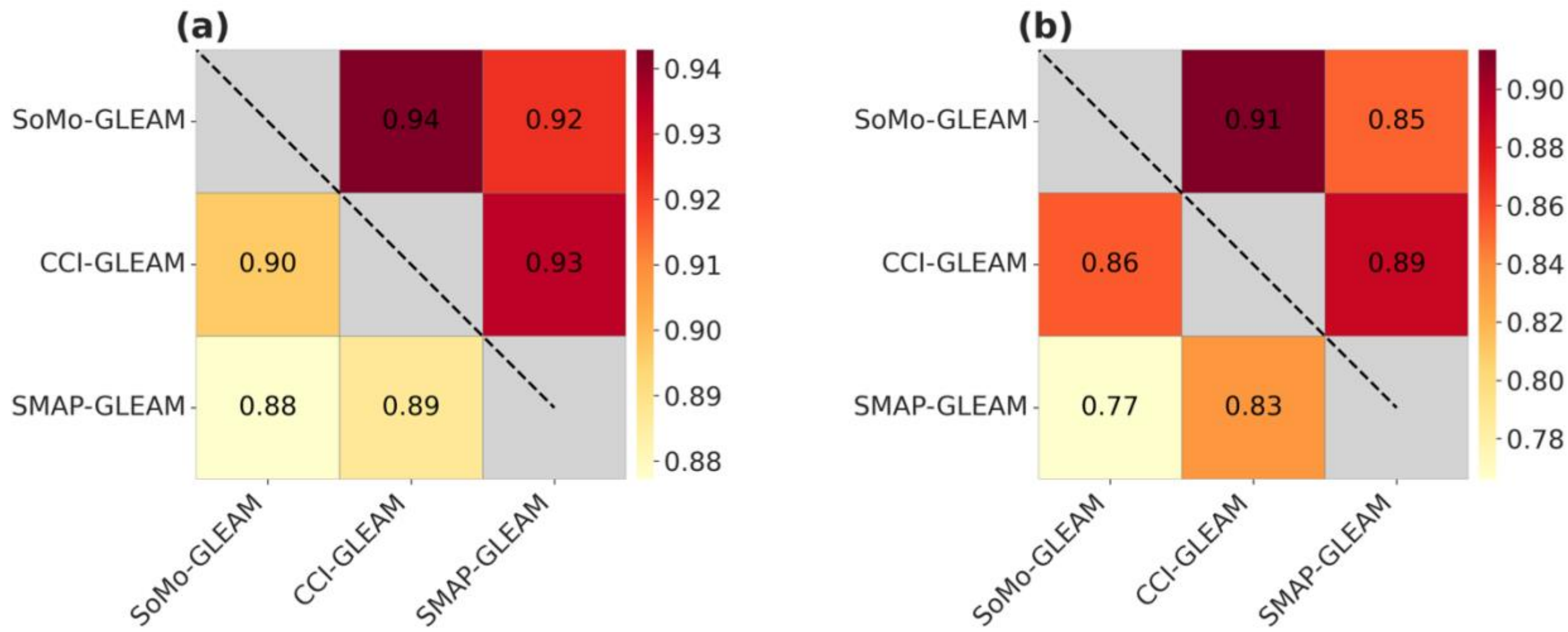

Figure 5. Pairwise Spearman rank correlations among (upper triangle) the adjusted terrestrial coupling index combinations and (lower triangle) the adjusted correlation, but showing (a) SM and EF anomalies, and (b) SM and H anomalies.

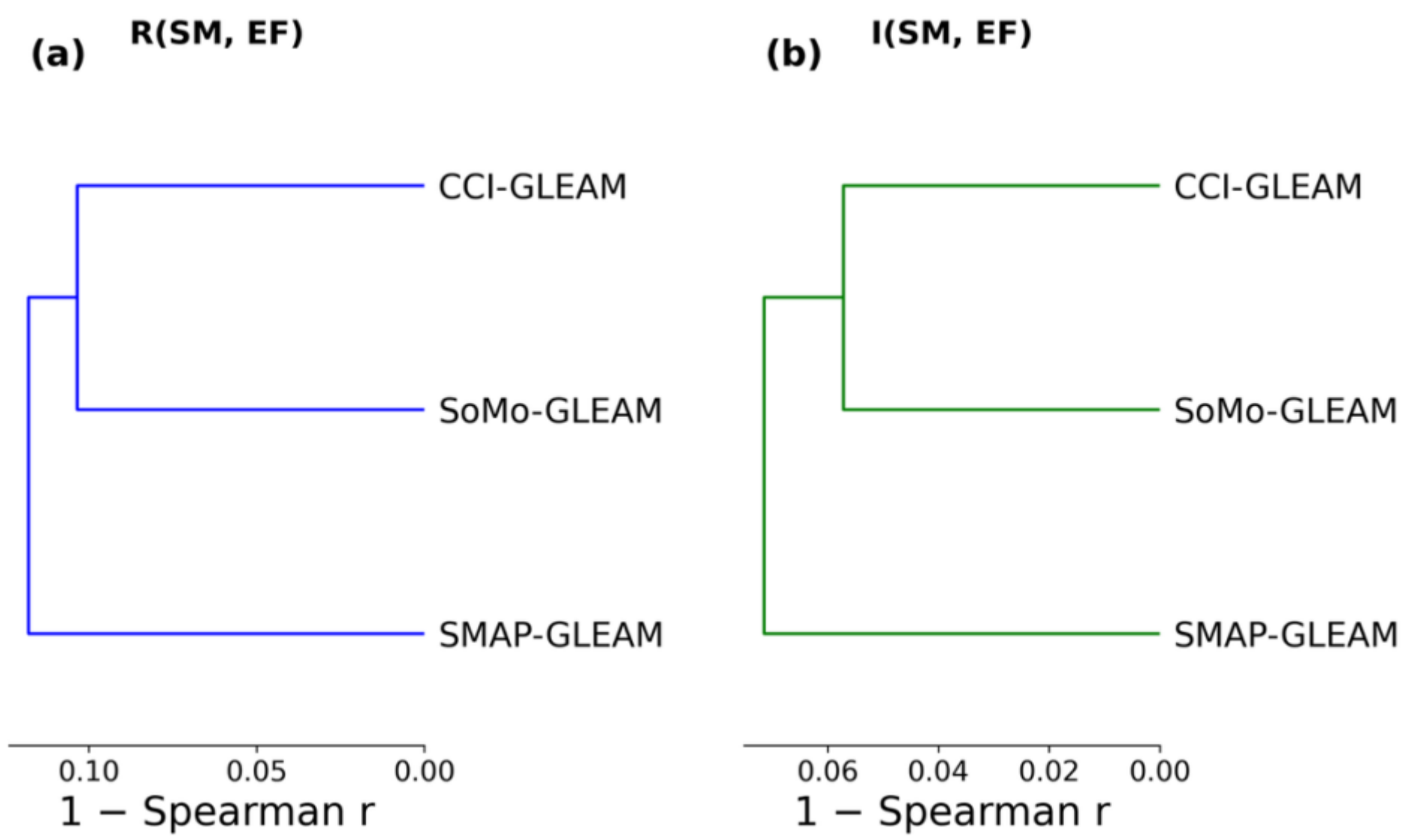

Figure 6. Same as in Figure 4, but for (a) $R_{Adjusted}(SM, EF)$, and (b) $I_{Adjusted}(SM, EF)$, using combinations of soil moisture products (SMAP, SoMo, CCI) and evaporative fraction datasets (GLEAM).

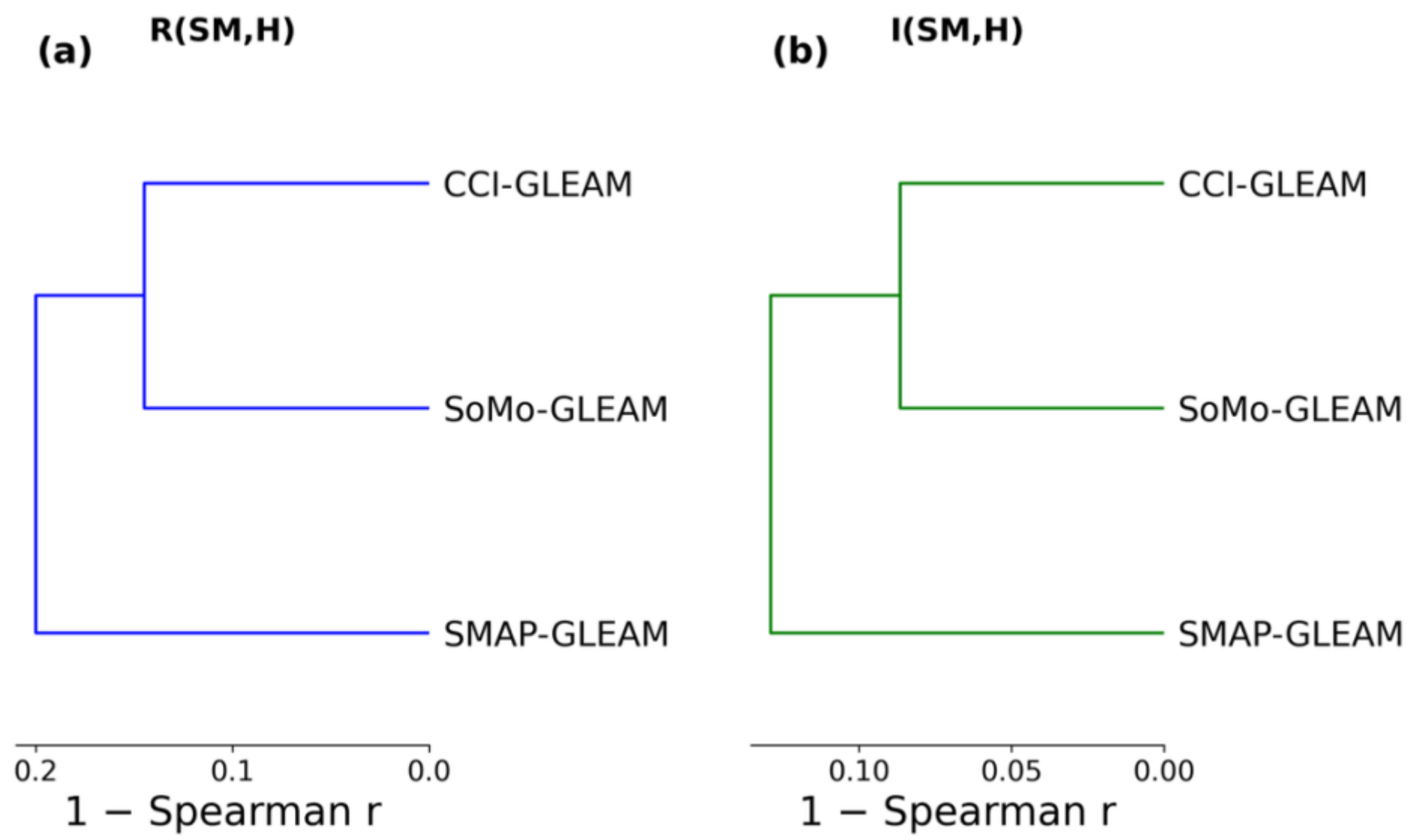

Figure 7. Same as in Figure 4, but for (a) $R_{Adjusted}(SM, H)$, and (b) $I_{Adjusted}(SM, H)$, using combinations of soil moisture products (SMAP, SoMo, CCI) and sensible heat flux datasets (GLEAM).

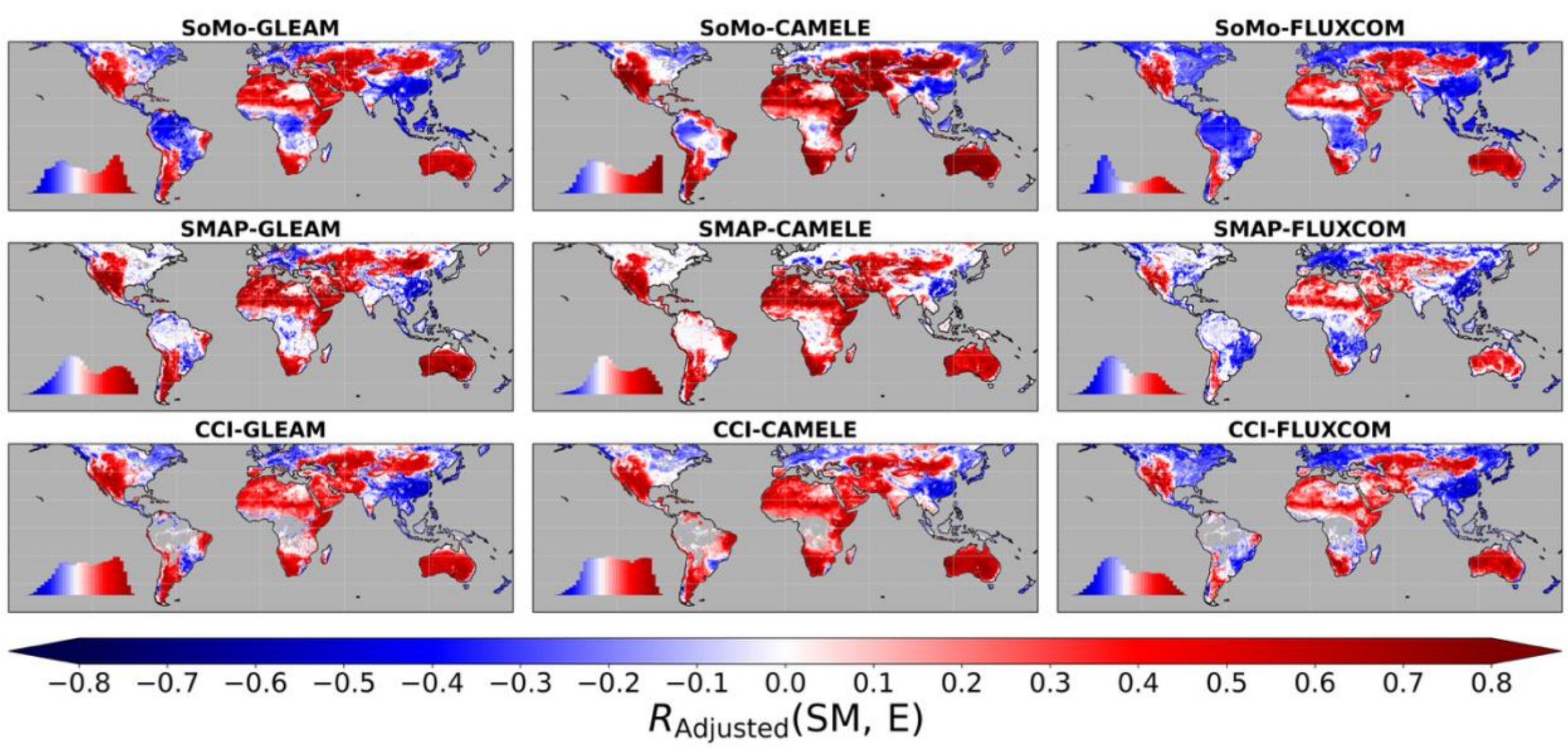

Figure 8. Global distribution of the adjusted correlation between daily evaporation and soil moisture anomalies derived from three soil moisture products (SoMo-L1, SMAP, CCI; arranged top to bottom) paired with three evaporation products (GLEAM, CAMELE, FLUXCOM; arranged left to right). Each map includes a histogram that summarizes the distribution of $R_{Adjusted}(SM, E)$ for that product pair, using the same color coding as the color bar. Gray areas indicate missing values, and white indicates grid cells where $R_{Adjusted}(SM, E)$ is not significant at the 5% significance level (two-sided Student's t-test; $p \geq 0.05$).

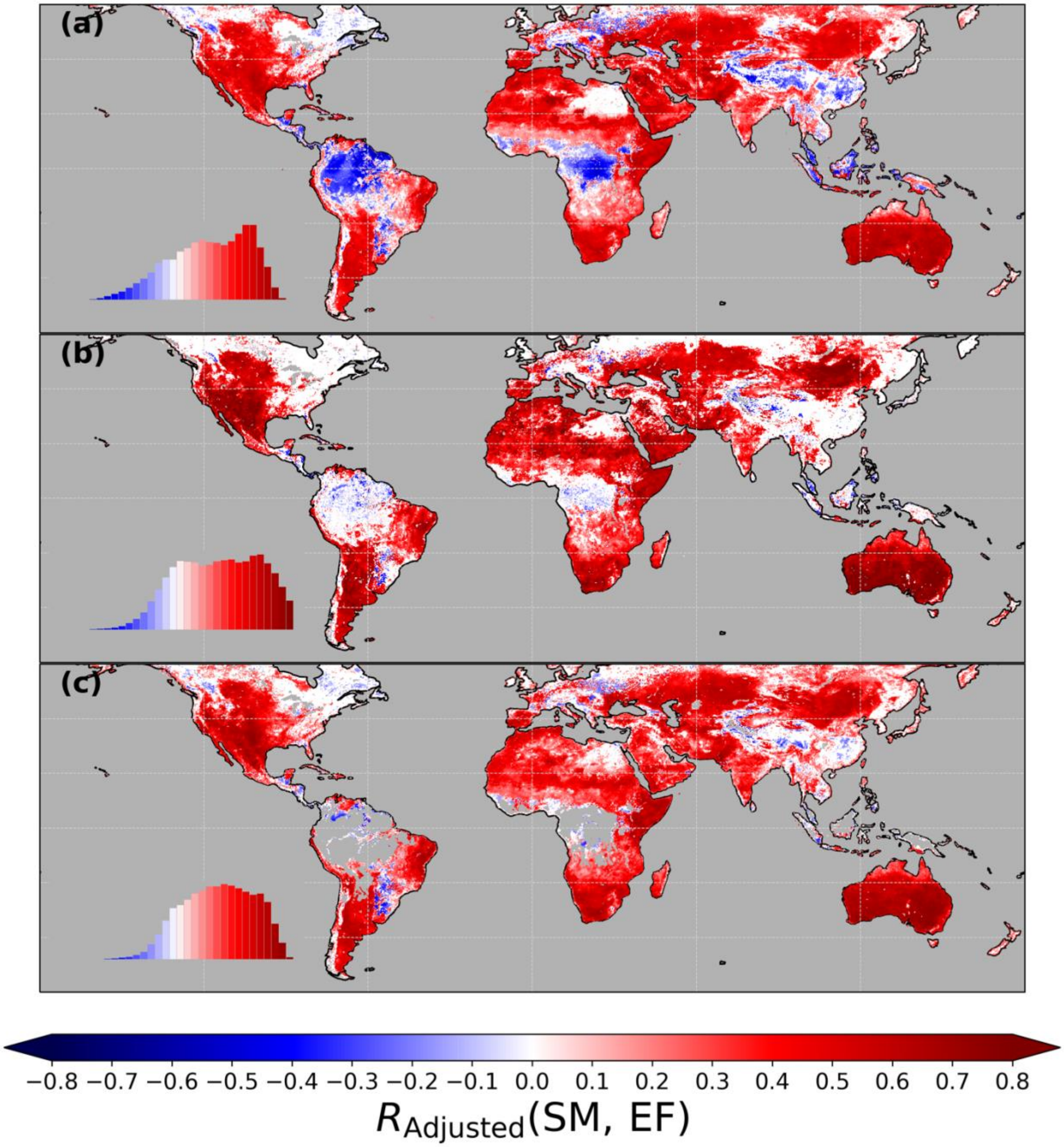

Figure 9. Same as in Figure 8, but for $R_{Adjusted}(SM, EF)$. White areas indicate grid cells where $R_{Adjusted}(SM, EF)$ is not significant at the 5% significance level (two-sided Student's t-test; $p \geq 0.05$).

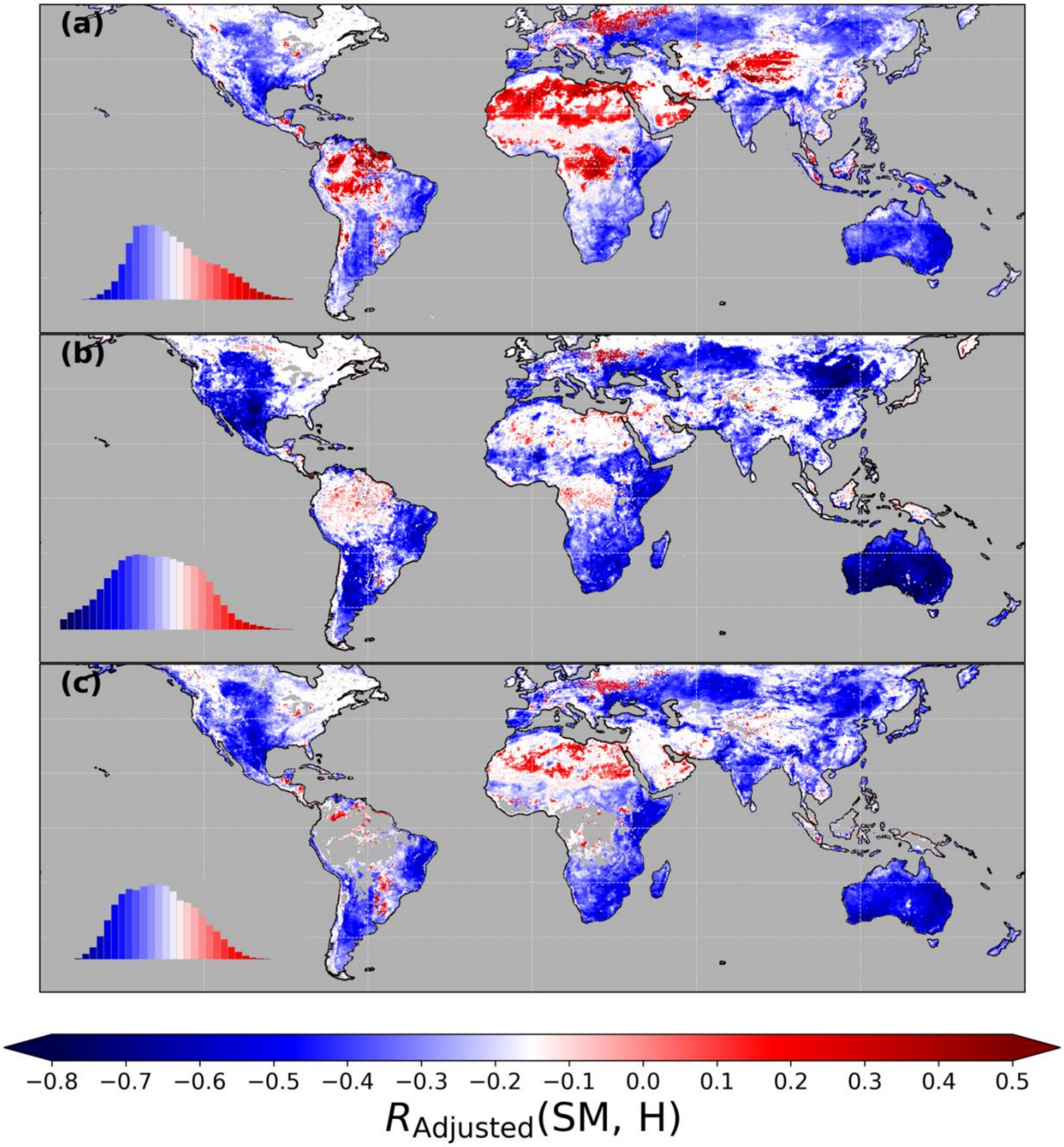

Figure 10. Same as in Figure 8, but for $R_{Adjusted}(SM, H)$. White areas indicate grid cells where $R_{Adjusted}(SM, H)$ is not significant at the 5% significance level (two-sided Student's t-test; $p \geq 0.05$).

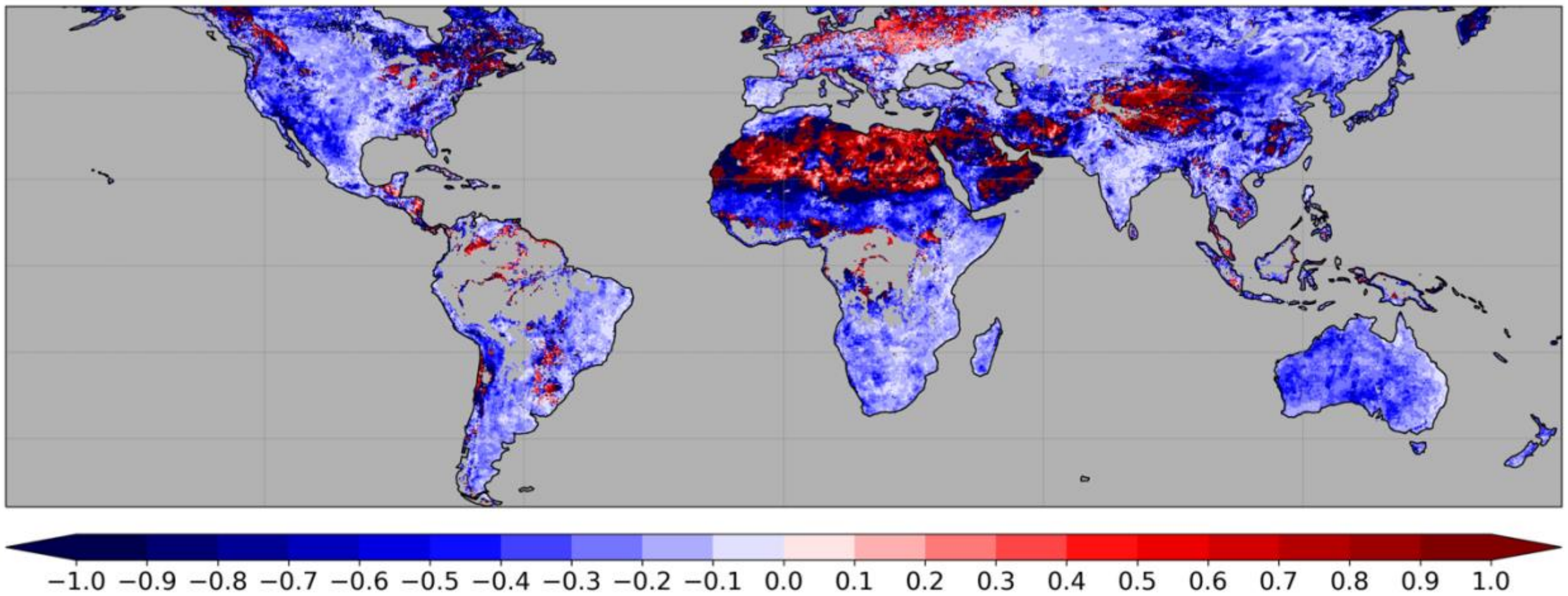

Figure 11. Global distribution of the coefficient of variation (CV) in Fisher z-space among $R_{Adjusted}(SM, H)$ pairs. Gray areas indicate missing values.

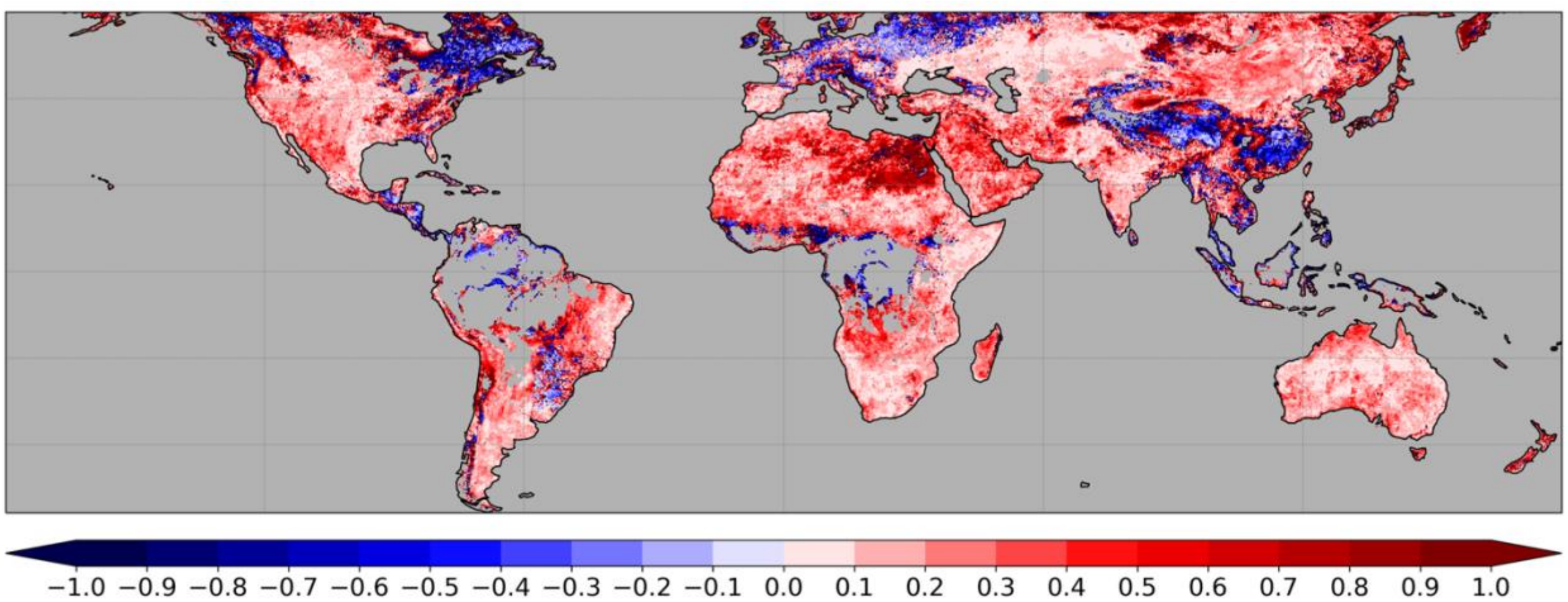

Figure 12. Same as in Figure 11, but among $R_{Adjusted}(SM, EF)$ pairs.

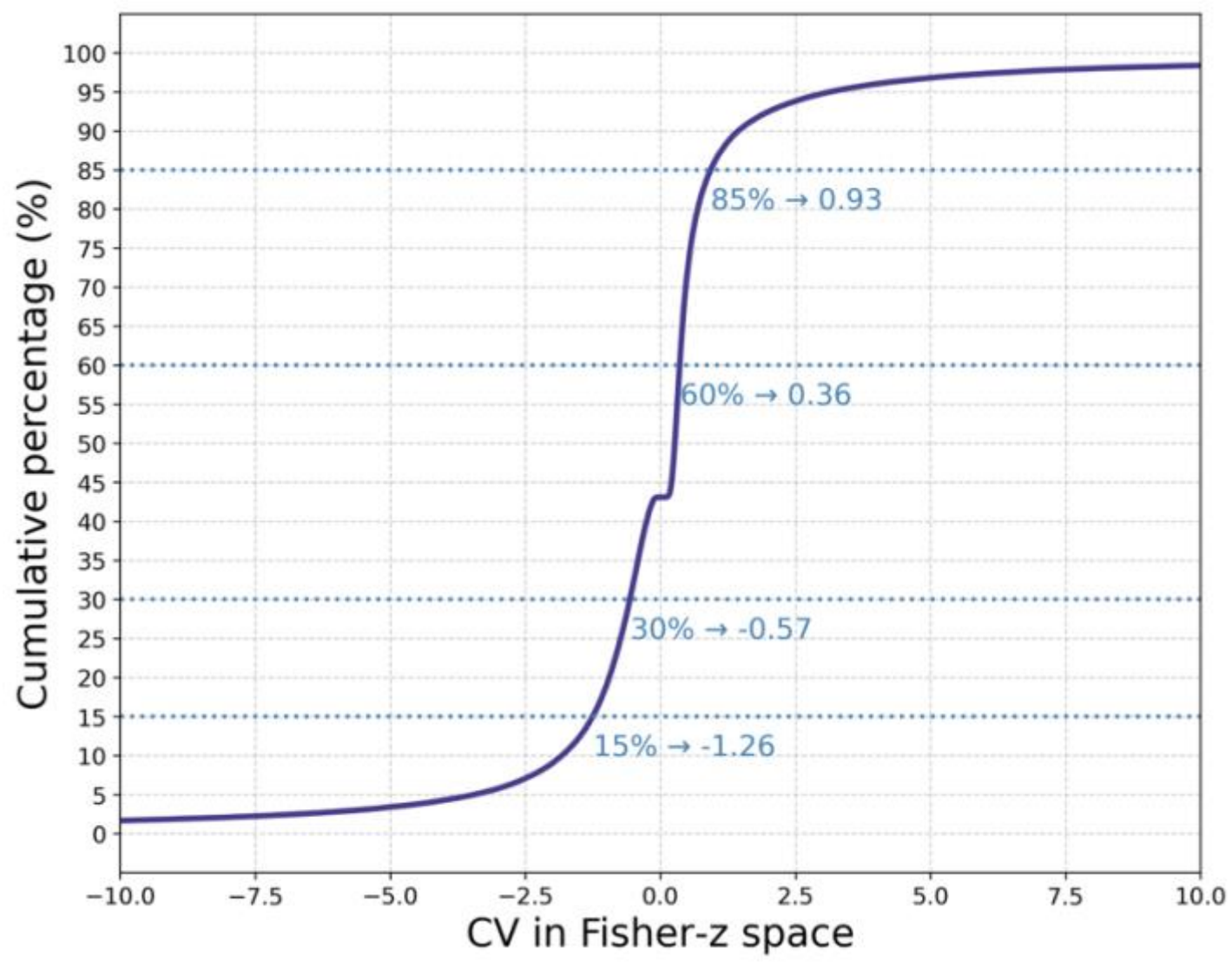

Figure 13. Cumulative distribution of the coefficient of variation (CV) in Fisher z-space among nine $R_{Adjusted}(SM, E)$ pairs. Marked percentiles (15%, 30%, 60%, and 85%) represent empirical thresholds used to classify regions into low, medium, and high dispersion categories for both energy- and water-limited regimes in the global classification.

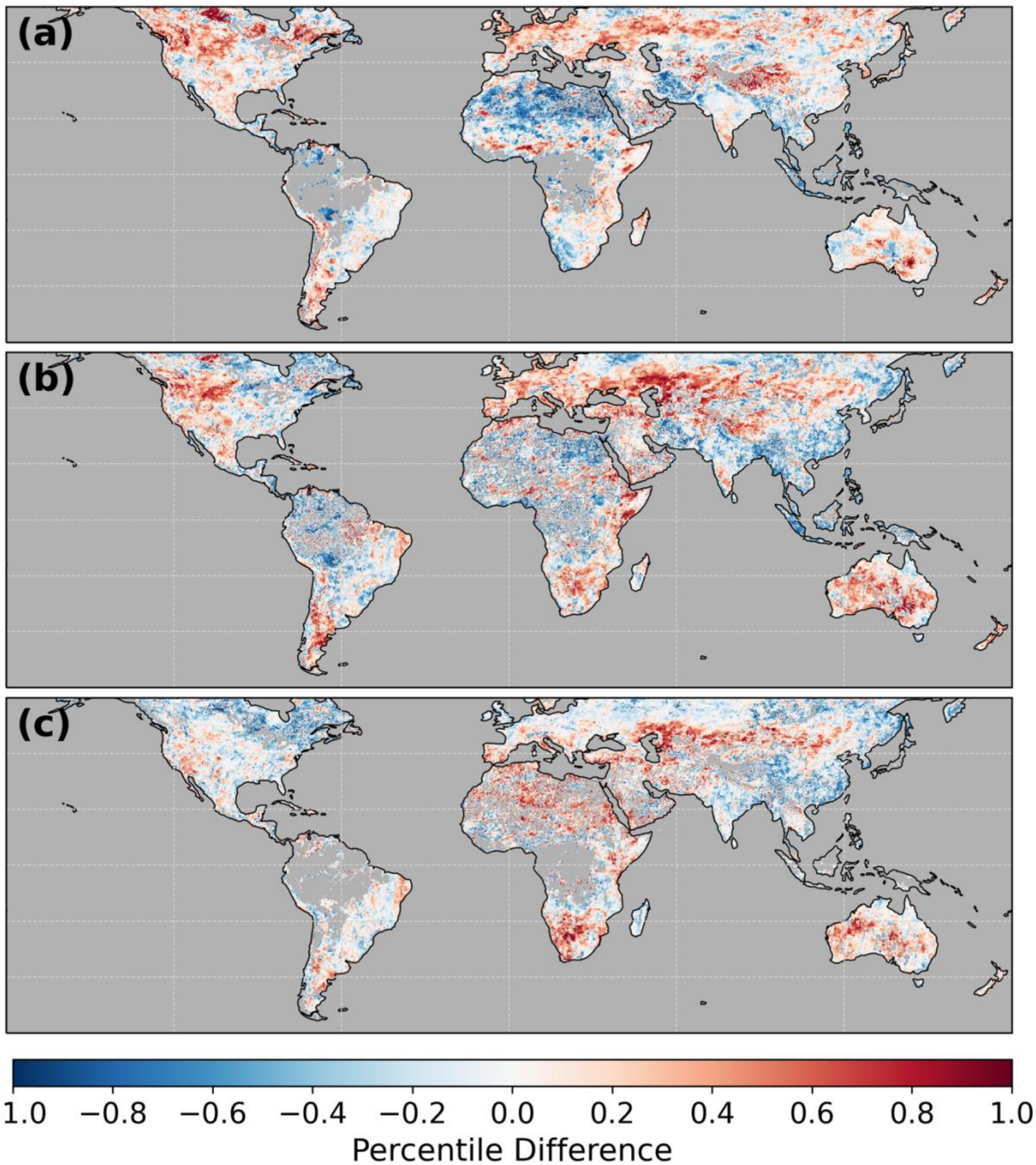

Figure 14. Global maps of percentile differences in adjusted soil moisture memory: (a) SoMo-L1 - CCI, (b) SoMo-L1 - SMAP, and (c) CCI – SMAP. Gray areas indicate missing values.

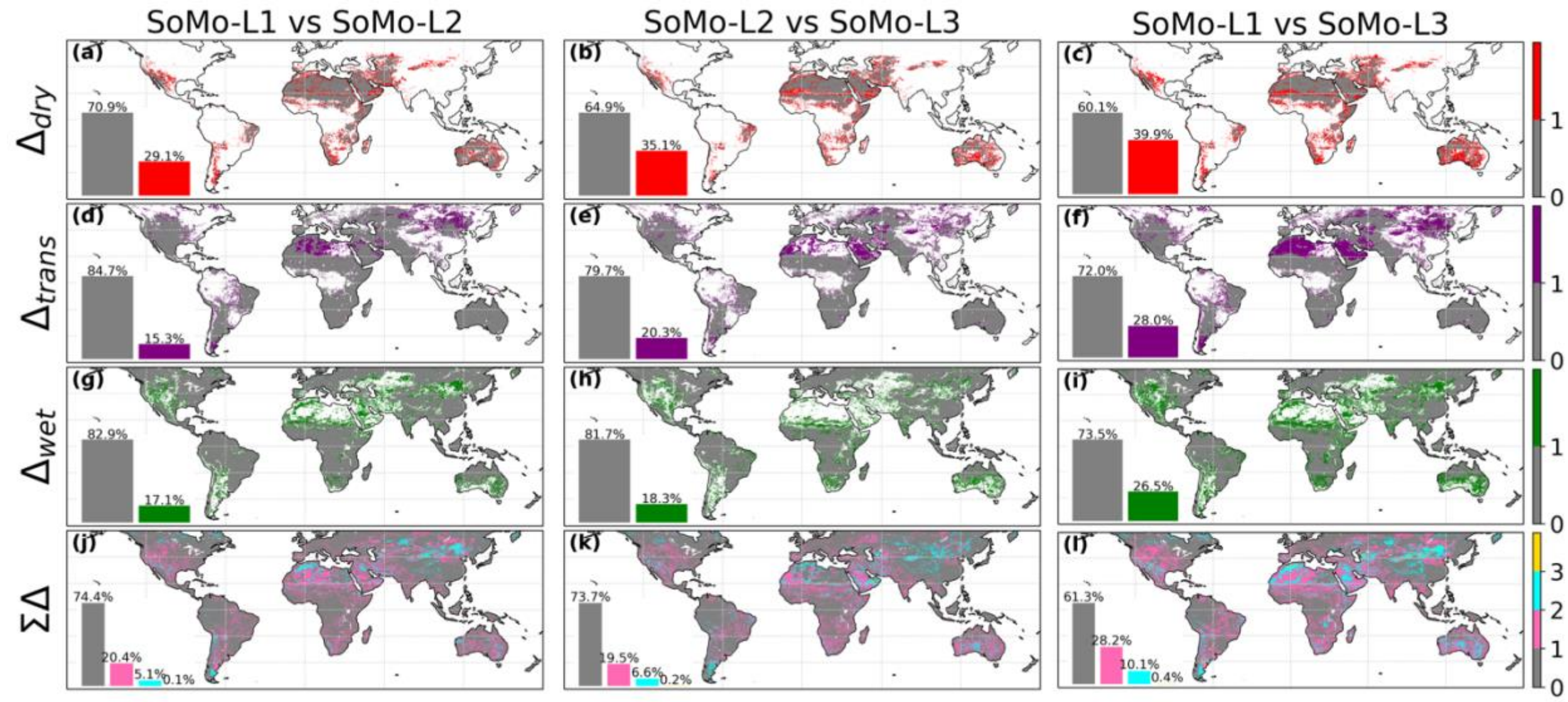

Figure 15. Spatial consistency of subsurface SM regime classifications between different SM layers shown by columns (left to right): SoMo-L1 vs. SoMo-L2, SoMo-L2 vs. SoMo-L3, SoMo-L1 vs. SoMo-L3. Rows show absolute differences for dry-inclusive digit (Δdry), transitional-inclusive digit (Δtrans), wet-inclusive digit (Δwet), and the total sum of disagreement index (ΣΔ). Grey cells indicate complete agreement between products (Δ= 0). Colored pixels denote disagreement: red for Δdry, purple for Δtrans, green for Δwet, and grey, pink, cyan, or gold for ΣΔ values of 0, 1, 2, or 3, respectively. The inserted bar charts display the fraction of land area (in %) occupied by each category for the corresponding map.

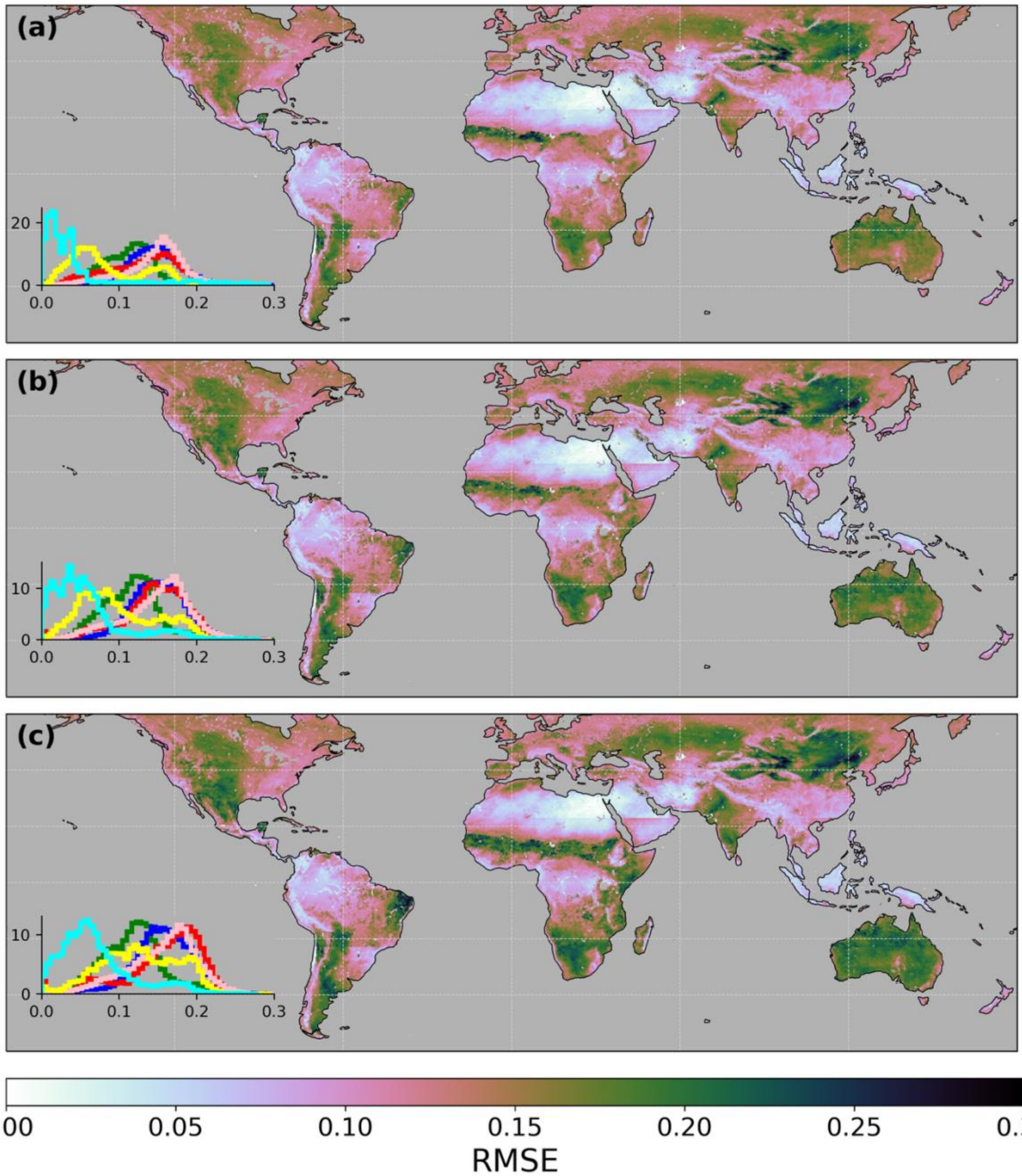

Figure 16. Global distribution of the root mean square error (RMSE) between the most likely SM-EF candidate model and the actual EF values across different SM layers of SoMo.ml: (a) SoMo-L1-GLEAM, (b) SoMo-L2-GLEAM, and (c) SoMo-L3-GLEAM. Each panel includes the PDFs (%) of RMSE across all grid cells identified for each segmented-linear regression model: green (001), blue (011), red (111), pink (010), yellow (110), and cyan (100). Gray areas indicate missing data.

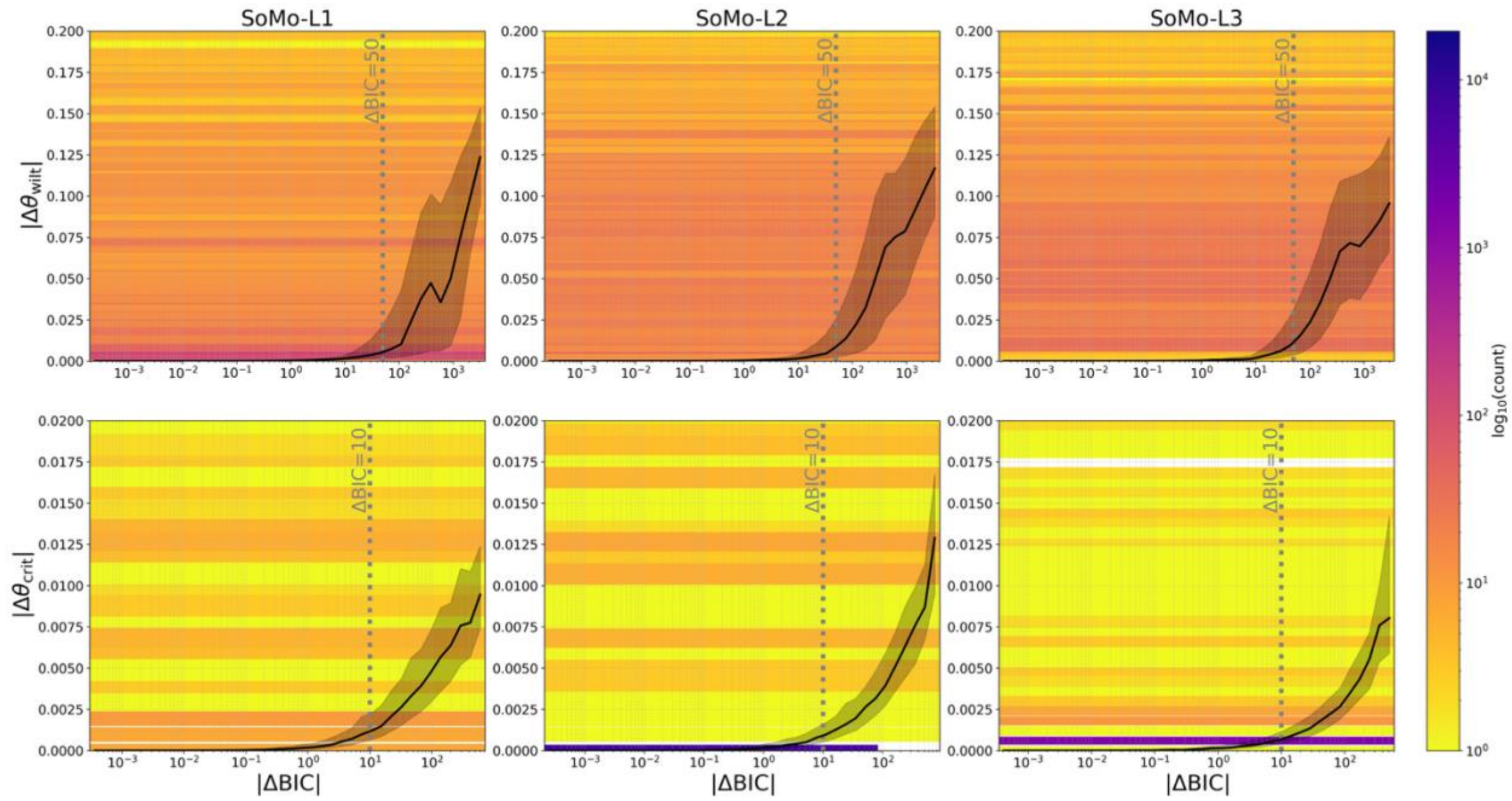

Figure 17. Hexbin plots showing the distribution of absolute differences in first- and second-best model breakpoints ($|\Delta\theta_{wilt}|$: first row, $|\Delta\theta_{crit}|$: second row) as a function of absolute differences in their BICs ($|\Delta BIC|$) for different SM layers of SoMo.ml: SoMo-L1, SoMo-L2, SoMo-L3; arranged from left to right. Black lines represent median values of $|\Delta\theta_{wilt}|$ and $|\Delta\theta_{crit}|$ calculated in logarithmically spaced bins of $|\Delta BIC|$ with shaded areas indicating interquartile ranges (25th-75th percentiles). Vertical dotted lines indicate $|\Delta BIC|$ thresholds ($|\Delta BIC|$ = 50 for θ_{wilt}, $|\Delta BIC|$ = 10 for θ_{crit}) used in breakpoint selection scenarios. The color bar represents the logarithm of point density ($\log_{10}$ count).

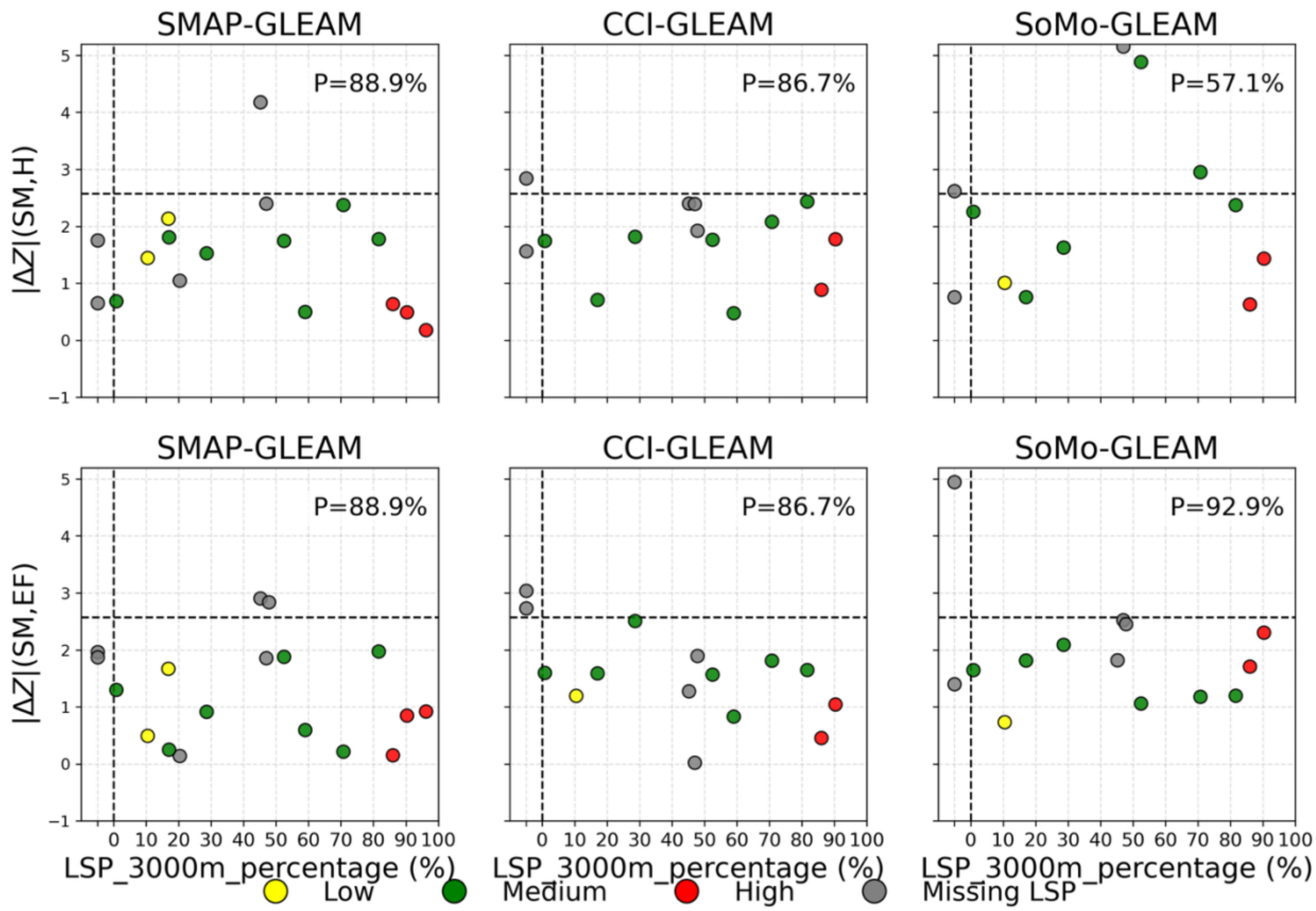

Figure 18. Scatter plots of absolute Z-score differences between the adjusted and AmeriFlux in-situ correlations of daily sensible heat flux–soil moisture anomalies (first row) and daily evaporative fraction–soil moisture anomalies (second row), plotted against the percentage of the dominant land cover type within a 3000 m radius (LSP_3000m_percentage) on the x-axis. Each marker represents a flux-tower site; colors denote the footprint-to-area representativeness index based on daytime daily footprint climatology within a 3000 m radius (yellow = Low, green = Medium, red = High, grey = No reporting for area representativeness index). The dashed horizontal line indicates the two-tailed 1 % p-value threshold $|\Delta Z| = 2.576$; markers below this line indicate that the adjusted and in-situ correlations are not significantly different at $\alpha = 0.01$. The markers on the left of the vertical line are sites where their LSP_3000m_percentage is not reported. The P in each panel shows the percentage of tower sites with correlations that do not significantly differ from adjusted correlations at a p-value of 1%. Panels are organized by SM products (columns: SMAP, CCI, SoMo-L1) coupled with the GLEAM product.

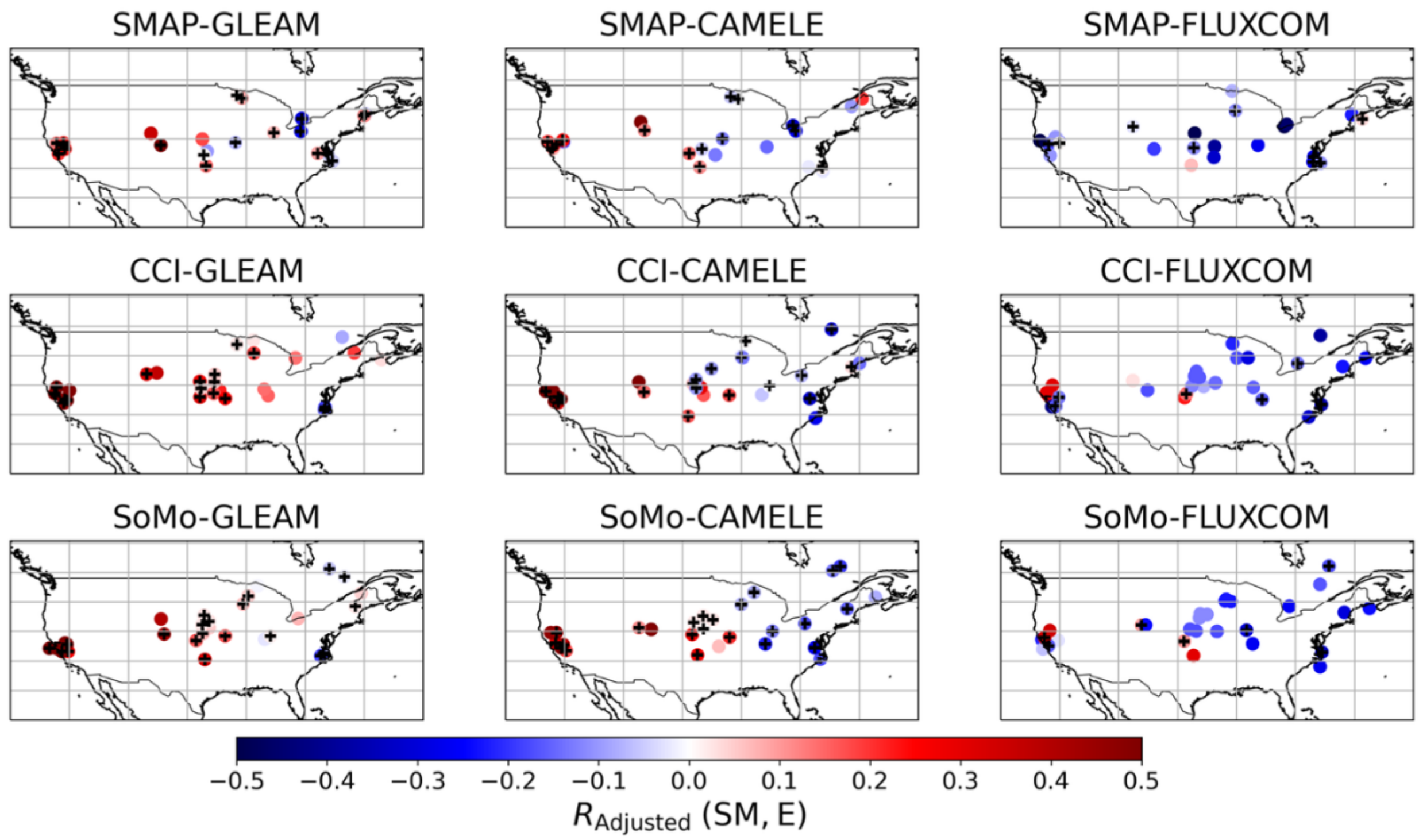

Figure 19. Spatial distribution of the adjusted correlation between daily evaporation and soil moisture anomalies, $R_{Adjusted}(SM, E)$, derived from three soil moisture products (SMAP; CCI; SoMo-L1; arranged top to bottom) paired with three evaporation products (GLEAM, CAMELE, FLUXCOM; arranged left to right) at AmeriFlux sites. In each panel, a cross (+) mark indicates AmeriFlux sites where adjusted correlations are not significantly different from in-situ correlations, with a p-value of 1%.

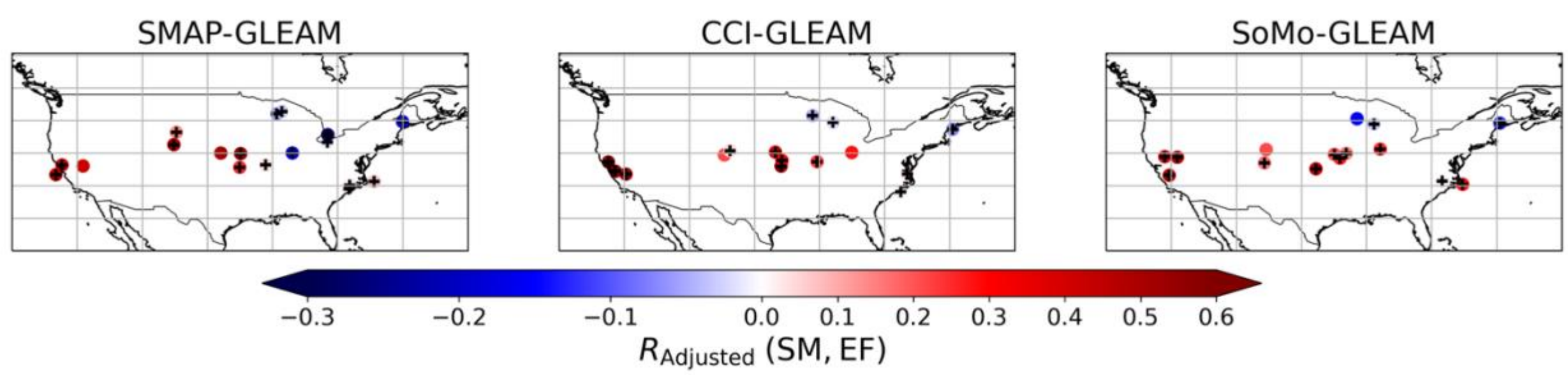

Figure 20. Same layout as Figure 19, but for $R_{Adjusted}(SM, EF)$ at the AmeriFlux sites.

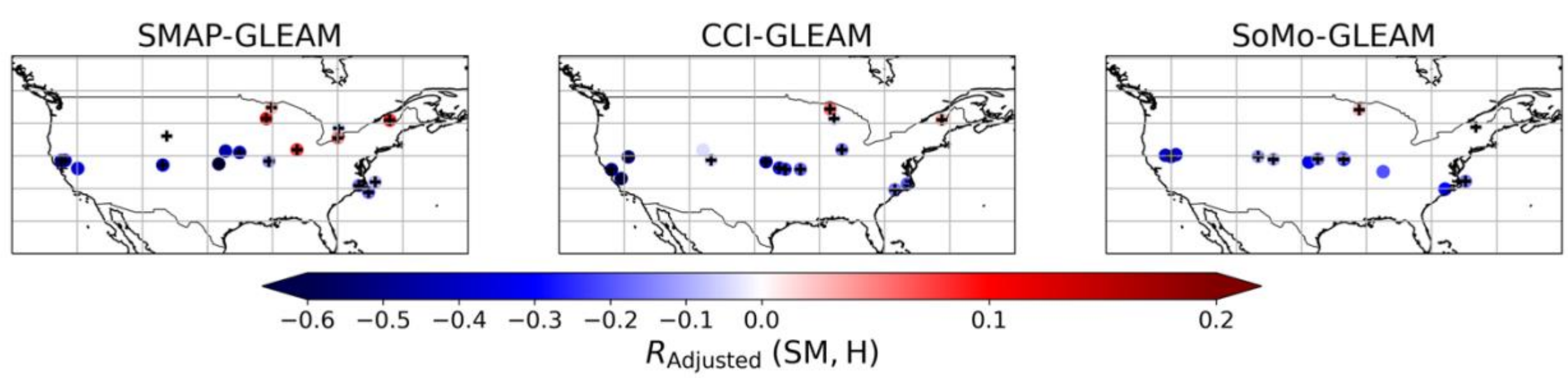

Figure 21. Same layout as Figure 19, but for $R_{Adjusted}(SM, H)$ at the AmeriFlux sites.

Table 1. Information for the AmeriFlux sites used in the study.

Site	Name	Lat.	Lon.	SWC	LE	TS	H	start	end	DOI
**CA-Qc2**	Quebec - 1975 Harvested Black Spruce (HBS75)	49.7598	-74.5711	SWC_PI_1	LE_PI_F	TS_PI_F_2		2007	2010	https://doi.org/10.17190/AMF/1419514
**CA-Qfo**	Quebec - Eastern Boreal, Mature Black Spruce	49.6925	-74.3421	SWC_1_1_1	LE_1_1_1	TS_1_1_1		2003	2010	https://doi.org/10.17190/AMF/1246829
**CA-TP1**	Ontario - Turkey Point 2002 Plantation White Pine	42.6609	-80.5595	SWC_1_1_1	LE_PI_F	TS_PI_F_1	H_PI_F	2002	2023	https://doi.org/10.17190/AMF/1246009
**CA-TP2**	Ontario - Turkey Point 1989 Plantation White Pine	42.7744	-80.4588	SWC_1_1_1	LE_PI_F	TS_PI_F_1		2002	2008	https://doi.org/10.17190/AMF/1246010
**CA-TP3**	Ontario - Turkey Point 1974 Plantation White Pine	42.7068	-80.3483	SWC_1_1_1	LE_PI_F	TS_PI_F_1		2002	2023	https://doi.org/10.17190/AMF/1246011
**CA-TP4**	Ontario - Turkey Point 1939 Plantation White Pine	42.7102	-80.3574	SWC_1_1_1	LE_PI_F	TS_PI_F_1		2002	2023	https://doi.org/10.17190/AMF/1246012
**CA-TPD**	Ontario - Turkey Point Mature Deciduous	42.6353	-80.5577	SWC_1_1_1	LE_PI_F	TS_PI_F_1	H_PI_F	2012	2023	https://doi.org/10.17190/AMF/1246152
**US-ARM**	ARM Southern Great Plains site- Lamont	36.6058	-97.4888	SWC_2_1_1	LE_1_1_1	TS_2_1_1		2003	2025	https://doi.org/10.17190/AMF/1246027
**US-BRG**	Bayles Road Grassland Tower	39.2167	-86.5406	SWC_1_1_1	LE_1_1_1	TS_1_1_1	H_1_1_1	2016	2020	https://doi.org/10.17190/AMF/1756416
**US-Bar**	Bartlett Experimental Forest	44.0646	-71.2881	SWC_1_1_1	LE_1_1_1	TS_1_1_1	H_1_1_1	2004	2024	https://doi.org/10.17190/AMF/1246030
**US-Bi1**	Bouldin Island Alfalfa	38.0992	-121.4993	SWC_PI_1	LE_PI_F	TS_1_1_1	H_PI_F	2016	2025	https://doi.org/10.17190/AMF/1480317
**US-Bi2**	Bouldin Island corn	38.1091	-121.5351	SWC_2_1_1	LE_PI_F	TS_2_1_1		2017	2025	https://doi.org/10.17190/AMF/1419513
**US-GLE**	GLEES	41.3665	-106.2399	SWC_3_1_1	LE_PI_F_1_1_1	TS_3_1_1	H_PI_F_1_1_1	1999	2020	https://doi.org/10.17190/AMF/1246056

				SWC_1_1_1	LE_PI_F_1_1_1	TS_1_1_1				
**US-Ha1**	Harvard Forest EMS Tower (HFR1)	42.5378	-72.1715	SWC_7_1_1	LE_1_1_1	TS_7_1_1		1991	2025	https://doi.org/10.17190/AMF/1246059
**US-Ho1**	Howland Forest (main tower)	45.2041	-68.7402	SWC_3_1_1	LE_1_1_1	TS_3_1_1		1996	2025	https://doi.org/10.17190/AMF/1246061

Site	Name	Lat.	Lon.	SWC	LE	TS	H	start	end	DOI
**US-KFS**	Kansas Field Station	39.0561	-95.1907	SWC_2_1_1	LE_PI_F_1_1_1	TS_2_1_1	H_PI_F_1_1_1	2007	2019	https://doi.org/10.17190/AMF/1246132
**US-KLS**	Kansas Land Institute	38.7745	-97.5684	SWC_2_1_1	LE_PI_F_1_1_1	TS_2_1_1	H_PI_F_1_1_1	2012	2019	https://doi.org/10.17190/AMF/1498745
**US-Kon**	Konza Prairie LTER (KNZ)	39.0824	-96.5603	SWC_2_1_1	LE_1_1_1	TS_2_1_1	H_1_1_1	2004	2019	https://doi.org/10.17190/AMF/1246068
**US-MMS**	Morgan Monroe State Forest	39.3232	-86.4131	SWC_PI_1	LE_1_1_1	TS_2_1_1		1999	2025	https://doi.org/10.17190/AMF/1246080
**US-MOz**	Missouri Ozark Site	38.7441	-92.2	SWC_1_1_1	LE_1_1_1	TS_1_1_1	H_1_1_1	2004	2021	https://doi.org/10.17190/AMF/1246081
**US-NC2**	NC_Loblolly Plantation	35.803	-76.6685	SWC_1_1_1	LE_1_1_1	TS_1_1_1	H_1_1_1	2005	2025	https://doi.org/10.17190/AMF/1246083
**US-NC3**	NC_Clearcut#3	35.799	-76.656	SWC_1_1_1	LE_1_1_1	TS_1_1_1	H_1_1_1	2013	2021	https://doi.org/10.17190/AMF/1419506
**US-NC4**	NC_AlligatorRiver	35.7879	-75.9038	SWC_1_1_1	LE_1_1_1	TS_1_1_1	H_1_1_1	2009	2025	https://doi.org/10.17190/AMF/1480314
**US-NR1**	Niwot Ridge Forest (LTER NWT1)	40.0329	-105.5464	SWC_PI_F_1_1_1	LE_PI_F_1_1_1	TS_PI_F_1_1_1	H_PI_F_1_1_1	1998	2025	https://doi.org/10.17190/AMF/1246088
**US-Ne1**	Mead - irrigated continuous maize site	41.1651	-96.4766	SWC_PI_F_2_1_1	LE_1_1_1	TS_PI_F_2_1_1		2001	2024	https://doi.org/10.17190/AMF/1246084
**US-Ne2**	Mead - irrigated maize-soybean rotation site	41.1649	-96.4701	SWC_PI_F_1_1_1	LE_1_1_1	TS_PI_F_1_1_1		2001	2024	https://doi.org/10.17190/AMF/1246085
**US-Ne3**	Mead - rainfed maize-soybean rotation site	41.1797	-96.4397	SWC_PI_F_1_1_1	LE_1_1_1	TS_PI_F_1_1_1		2001	2024	https://doi.org/10.17190/AMF/1246086
**US-PFa**	Park Falls/WLEF	45.9459	-90.2723	SWC_1_1_1	LE_1_2_1	TS_1_1_1		1995	2025	https://doi.org/10.17190/AMF/1246090
**US-Snf**	Sherman Barn	38.0402	-121.7272	SWC_PI_1	LE_PI_F	TS_PI_1		2018	2020	https://doi.org/10.17190/AMF/1579718
**US-Syv**	Sylvania Wilderness Area	46.242	-89.3477	SWC_1_1_1	LE_1_1_1	TS_PI_F_1_1_1	H_1_1_1	2001	2025	https://doi.org/10.17190/AMF/1246106

**US-Ton**	Tonzi Ranch	38.4309	-120.966	SWC_PI_F_1_1_A	LE_PI_F_1_1_1	TS_PI_F_1_1_A	H_PI_F_1_1_1	2001	2025	https://doi.org/10.17190/AMF/1245971
**US-Tw3**	Twitchell Alfalfa	38.1152	-121.6469	SWC_1_1_1	LE_PI_F	TS_1_1_1		2013	2018	https://doi.org/10.17190/AMF/1246149
**US-Var**	Vaira Ranch- Ione	38.4133	-120.9508	SWC_PI_F_1_1_A	LE_PI_F	TS_PI_F_1_1_A	H_PI_F	2000	2025	https://doi.org/10.17190/AMF/1245984
**US-WCr**	Willow Creek	45.8059	-90.0799	SWC_2_1_1	LE_1_1_1	TS_2_1_1	H_1_1_1	1998	2025	https://doi.org/10.17190/AMF/1246111
